# Comparative efficacy of traditional non-pharmacological add-on treatments in patients with stable chronic obstructive pulmonary disease: a systematic review and network meta-analysis

**DOI:** 10.3389/fpubh.2025.1410342

**Published:** 2025-02-21

**Authors:** Ji-Ae Roh, Jungtae Leem, Beom-Joon Lee, Kwan-Il Kim, Hee-Jae Jung

**Affiliations:** ^1^Department of Clinical Korean Medicine, College of Korean Medicine, Graduate School, Kyung Hee University, Seoul, Republic of Korea; ^2^Division of Allergy, Immune and Respiratory System, Department of Internal Medicine, College of Korean Medicine Kyung Hee University, Kyung Hee University Medical Center, Seoul, Republic of Korea; ^3^Department of Diagnostics, College of Korean Medicine, Wonkwang University, Iksan, Republic of Korea; ^4^Department of Il-won Integrated Medicine, Wonkwang University Korean Medicine Hospital, Iksan, Republic of Korea

**Keywords:** chronic obstructive pulmonary disease, non-pharmacological treatment, AddOn, systematic review, network meta-analysis

## Abstract

Chronic obstructive pulmonary disease (COPD) is a major global public health concern. In this study, we examined the comparative efficacy of non-pharmacological interventions within East Asian traditional medicine (EATM-NPI) for enhancing pulmonary function and exercise capacity in patients with stable COPD. A thorough search of electronic databases conducted until May 22, 2022, identified studies employing EATM-NPI in such patients. The evaluation focused on the impact adjunctive therapies on pulmonary function (forced expiratory volume in 1 s [FEV_1_]) and exercise capacity (6-min walking distance [6MWD]). The qualitative assessment encompassed 142 studies, with 133 studies included in one of three network meta-analyses. Participants, aged 49–76 years, ranged from 9 to 139 per group, predominantly from China (87.7% of studies). Overall study quality was generally low, and reported adverse events were mild. Notably, moxibustion and qigong adjunctive therapies demonstrated significant improvements in FEV_1_ (L) and FEV_1_ (%). Additionally, chuna, acupuncture, qigong and moxibustion adjunctive therapies were associated with significant improvements in 6MWD. In conclusion, EATM-NPI adjunctive therapy, when combined with standard pharmacological treatment, exhibited effects on pulmonary function and exercise capacity in patients with COPD.

**Systematic review registration:** The protocol was registered with PROSPERO (CRD42023389431), https://www.crd.york.ac.uk/prospero/display_record.php?ID=CRD42023389431.

## 1 Introduction

Chronic obstructive pulmonary disease (COPD) is a disease marked by irreversible airway obstruction on the pulmonary function test along with symptoms such as chronic dyspnea, cough, and sputum (1). COPD is a major disease that poses economic and social burdens, and its global burden is consistently increasing (2–4). In 2017, COPD was the third leading cause of death globally (5). In 2019, the global prevalence of COPD was 10.3% with approximately 391.9 million people estimated to be have been affected by the disease (5, 6). Its prevalence in East Asia has been reported to be slightly higher than the global average. The trends in the prevalence of COPD in South Korea were reported within the range of approximately 13.1 to 14.6% during the period from 2010 to 2015 (7), and that in China was 13.6% during 2014–2015 (8). COPD typically manifests in individuals aged 40 years and older; thus, its prevalence is expected to increase in an aging society. Therefore, COPD has been incorporated into the WHO Global Action Plan to prevent and control non-communicable diseases.

To mitigate the mounting burden of respiratory illnesses that have surged since the 20th century, it is important to explore alternative strategies that can more effectively manage COPD in conjunction with the standard pharmacological therapy proposed by the Global Initiative for Chronic Obstructive Lung Disease (GOLD) (9). Pharmacological therapy alone falls short of effectively managing these chronic, irreversible symptoms and respiratory exacerbations, along with systemic repercussions. Therefore, non-pharmacological therapy plays a pivotal role in managing patients with stable COPD (1).

The non-pharmacological intervention of East Asian traditional medicine (EATM-NPI) represents an enticing alternative that can be added to standard pharmacological therapy. The EATM-NPI encompasses various traditional medicine practices. Although acupuncture, which stimulates the surface of the body using needles, is the most well-known method, other EATM-NPIs include moxibustion, which involves thermal stimulation of the body’s surface; cupping therapy, which creates negative pressure on the skin; qigong, which involves breathing regulation and mental focus; and chuna, a manual therapy that alters the body’s function and structure through the practitioner’s hands. These EATM-NPIs have been sporadically studied for their potential to provide additional benefits to patients with COPD.

A network meta-analysis (NMA) comparing traditional exercise therapies revealed that Wuqinxi improved pulmonary function and exercise capacity, whereas Yijinjing improved quality of life (QOL) (10). A meta-analysis of studies on acupuncture-related treatments showed that acupressure enhances QOL, whereas acupuncture improves pulmonary function, exercise capacity, and QOL (11, 12). Furthermore, the 2023 GOLD guidelines presented acupuncture and acupressure as elective adjunctive therapies for dyspnea (1). In a meta-analysis on chuna therapy, the authors could not draw a concrete conclusion on whether chuna improves pulmonary function and exercise capacity, but they found that non-pharmacological therapies generally are effective on COPD, indicating that non-pharmacological therapies could be promising for COPD (13).

There is a dearth of comprehensive evidence for EATM-NPI as a new potential approach for the management and treatment of stable COPD. Nearly all studies on EATM-NPIs for patients with COPD show that they have been ethically designed as add-on therapies in conjunction with standard pharmacological treatments. Additionally, in actual East Asian clinical practice, various combinations of EATM-NPI therapies are used concurrently to treat patients with COPD (14–16), so clinicians face the challenge of determining which EATM-NPI therapy to prioritize for patients with COPD. While traditional pairwise meta-analysis (PMA) is limited to analyzing one intervention at a time, NMA allows for a relative ranking of the therapeutic efficacy of multiple interventions, thereby empowering clinicians and patients to make more informed decisions. This study aimed to investigate the comparative effects of EATM-NPIs added to standard pharmacological therapy for patients with stable COPD by using NMA. Therefore, here we aimed to investigate the therapeutic efficacy and comparative advantages of non-pharmacological therapies and derive treatment strategies for patients with stable COPD in an aging society.

## 2 Methods

This study adheres to the Preferred Reporting Items for Systematic Reviews and Meta-Analyses NMA guideline (Supplement 1) (17), and the protocol was registered with PROSPERO (CRD42023389431).

### 2.1 Criteria for inclusion and exclusion

The eligibility criteria were established based on the participant, intervention, comparison, outcome, and study design (PICOS) framework.

#### 2.1.1 Study types

Studies that made the diagnosis based on the GOLD guideline (1) or “forced expiratory volume in 1 s (FEV_1_)/forced vital capacity (FVC) <0.7” and specified the diagnosis were included. Randomized controlled trials (RCTs) and crossover RCTs were included, whereas non-RCTs, observational studies, case reports, and animal studies were excluded. If a particular arm did not meet the eligibility criteria in a multi-arm trial, that study was still included, but the data from the specific arm were not used.

#### 2.1.2 Participant types

Patients with stable COPD were included, and patients with acute exacerbation of COPD were excluded. There were no limitations on age, sex, and COPD severity. Patients who did not receive the standard pharmacological therapy as specified in the GOLD guideline during the study period were also excluded.

#### 2.1.3 Intervention types and controls

The main intervention was EATM-NPI. Herbal medicine and pharmacoacupuncture therapy were excluded. EATM-NPI was broadly categorized into qigong (unspecified qigong, Wuqinxi, Liuzijue, Yijinjing, Taichi, and Baduanjin), moxibustion, cupping, chuna (fascia chuna therapy, joint mobilization and distraction, and spine and joint manipulation), and acupuncture (traditional stimulation of acupoints without the use of thermal stimulation). The treatment for the control group was not limited, but studies that used additional drugs or an herbal regimen for the control group were excluded. The treatment of the control group was broadly divided into pharmacological treatment (pharmacological therapy specified in the GOLD guidelines), exercise therapy (aerobic exercise prescribed in PR that is not considered an EATM-NPI, e.g., pursed-lip breathing and abdominal respiration), and placebo (with blinded).

#### 2.1.4 Outcomes measures

The outcome measures were pulmonary function and exercise capacity. Pulmonary function was assessed based on FEV_1_ measured through spirometry, and exercise capacity was assessed based on the 6-min walking distance (6MWD). Specifically, FEV_1_ was measured using 2 units: FEV_1_ (L), which represents the actual exhalation volume, and FEV_1_ (%), which indicates the percentage of the predicted value based on the normal range for healthy individuals. Adverse events (AEs) reported in the included studies that reported FEV_1_ and 6MWD as the primary outcomes were collected.

### 2.2 Literature searches

A comprehensive search strategy was formulated using a combination of search terms pertaining to disease and intervention (Supplement 2). The language was not limited. The literature search was conducted in the following electronic databases from the beginning of the database to May 22, 2022: Medline via PubMed, EMBASE via Elsevier, CENTRAL (The Cochrane Central Register of Controlled Trials), China National Knowledge Infrastructure, DBpia, Korean Studies Information Service System, Research Information Service System, and Oriental Medicine Advanced Searching Integrated System.

### 2.3 Data selection

The references of the included studies and systematic reviews of relevant topic were manually searched. Ongoing protocols and conference abstracts were searched in Clinicaltrials.gov and the World Health Organization International Clinical Trials Registry Platform.

### 2.4 Data extraction

After uploading all the search results into EndNote X9.3.3 (Clarivate Analytics), two researchers (JAR and KIK) independently screened the titles and abstracts and then reviewed the full texts to select the studies. The same two researchers exported the title, author, publication. Year, country of origin, study design, enrolled and analyzed sample sizes, population age, disease severity, diagnostic criteria of COPD, treatment modalities and details of each assigned group, total treatment duration, treatment schedule, follow-up, reported AEs, and outcome measures of the studies as a spreadsheet (Microsoft Excel; Microsoft Corp.).

Any disagreements between the two researchers were resolved upon discussion. If an agreement could not be reached, a third researcher (JL) was involved to reach a consensus.

### 2.5 Quality/risk of bias assessment of included studies

Two researchers (JAR and KIK) independently assessed the risk of bias of the included studies using the Cochrane Handbook 5.1.0. assessment tool (18). The following items were rated as low risk, high risk, or unclear risk: random sequence generation, allocation concealment, blinding of participants and personnel, blinding of the outcome assessment, incomplete outcome data, selective reporting, and other bias.

### 2.6 Data analysis

First, PMA was performed for each intervention using Cochrane’s Review Manager (RevMan 5.4). The effect sizes of EATM-NPI were calculated based on all the included studies that could be included in the NMA. The primary outcome measures FEV_1_ (both L and %) and 6MWD were statistically analyzed using mean differences (MDs) and 95% confidence intervals (CIs). Data at the end of treatment were used; a positive MD indicated greater improvement of pulmonary function and exercise capacity.

A network model with a random effects model (19) was setup using the R package “netmeta (20)” for of the free software R-4.2.1 for Windows and RStudio (21) to conduct NMA using the frequentist method (22, 23). FEV_1_ was analyzed separately for each scale, and the results were presented in a forest plot and league table. P-scores, the probability of comparative advantage in non-pharmacological therapy, were calculated using the “netrank” function (21). The Egger’s test was used, and the results were visualized in a funnel plot to assess potential publication bias and overall bias of the included studies (21). Regarding the assumptions of NMA, connectivity was assessed using a network plot, and heterogeneity was assessed using I^2^. Network transitivity and consistency were verified through a global approach using Q-statistics, and consistency between direct and indirect comparisons was verified through a local approach using the node-splitting method.

## 3 Results

### 3.1 Study characteristics

**Figure 1:**
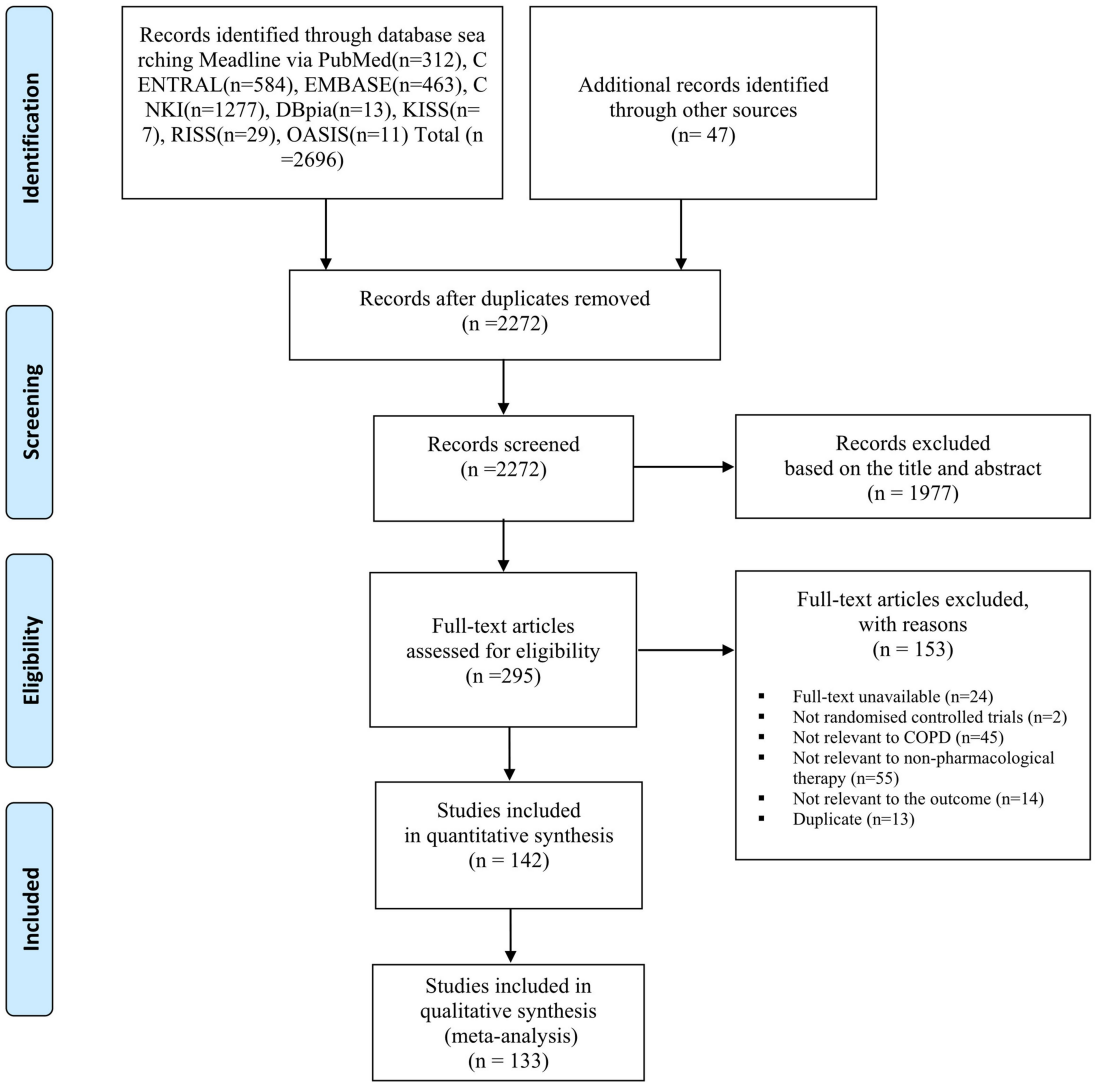
Flowchart of identification and screening for the eligible studies. COPD, chronic obstructive pulmonary disease; CNKI, China National Knowledge Infrastructure; KISS, Korean Studies Information Service System; RISS, Research Information Service System; OASIS, Oriental Medicine Advanced Searching Integrated System.

In total, 2,272 studies were identified, and after removing duplicate results and ineligible studies per PICOS, 142 studies were included (Figure 1) (24–165). With the exception of one cross-over RCT (133), all studies were RCTs. The number of participants assigned to each group was 9–139. Participants’ mean age was 49–76 years, and COPD severity varied. Most studies (87.7%) were conducted in China. No studies used cupping therapy as the intervention. The qigong group included qigong, health qigong integrated for lung health, Wuqinxi, Liujizue, Yijinjing, Taichi, and Baduanjin (24–110). The moxibustion group included unspecified moxibustion, ginger moxibustion, heat-sensitive point’s moxibustion, Yi Fei moxibustion, and fire-dragon moxibustion (111–130). The chuna group included osteopathic manipulative therapy, specialized physiotherapy, and chuna (131–136). The acupuncture group included filiform acupuncture, Acu-TENS, warm needle acupuncture, auricular acupressure, electroacupuncture, pressing needle acupuncture, and acupressure (137–165). Treatment duration ranged from once to 1 year (Table 1). If FVC was also reported in the included study, the data were presented in the table to aid in qualitative interpretation (Supplementary Table S1). Outcome variables presented as medians, mean differences, or points were excluded from the meta-analysis (33, 47, 64, 70, 71, 91, 105, 133, 142). Of 142 studies, 27 studies (27, 58, 64, 66–71, 86, 93, 132, 133, 137, 140, 141, 147, 150–156, 159, 163, 164) reported AEs (18.5%), and AEs requiring treatment were not reported (Table 1).

**Table 1:** Characteristics of the included studies.

Category		Author (year)/Country, City	Design study duration (wk)	Participants’ COPD severity	Group (N *analyzed)	Intervention	Outcome	Adverse event
Qigong	Qigong (气功)	Dong, Wang, Jia, Chen, and Ding (2021)/China, Jinan (24)	RCT	Stable COPD	10	Qigong exercise	6MWD, SGRQ, CAT	n.r
			12	GOLD 1–3	10	Cycle ergometer exercise		
	Health qigong integrated for lung health	Li (2019)/China, Shanghai (25)	Single-blind RCT	Stable COPD	18	Health qigong integrated for lung health	PFT (FEV_1_, FEV_1_%, FVC, FVC%, FEV_1_/FVC), 30-s elbow flexion test, 30-s sit-up test, 6MWD, 6MWD%pred, SGRQ, mMRC score	n.r
			12	GOLD 1–4	19	Usual care		
		Liu, Jin, Ng, Gu, Wu, and Lu (2012)/China, Shanghai (26)	Single-blind RCT	COPD	51	Health qigong integrated for lung health	PFT (FEV_1_%, FEV_1_/FVC), 6MWT, QoL score (“Zhong Shan COPD questionnaire”), immune cytokines (TNF-α, IL-8, IL-6), number of AECOPD	n.r
			24	GOLD 1–2	35	Usual care		
					32	PR		
		Liu (2011)/China, Jiangsu (27)	Single-blind RCT	Stable COPD	26	Health qigong integrated for lung health	PFT (FEV_1_%, FEV_1_/FVC), 6MWD, clinical symptom score, QoL score(“Zhong Shan COPD questionnaire”), ABGA (PaO_2_, PaCO_2_), number of AECOPD, immune cytokines (TNF-α, IL-8, IL-6)	0 “No significant adverse reactions were found in the three groups during the experiment.”
			24	GOLD 2	18	Usual care		
					17	Pursed-lip breathing, walking exercise		
	Wuqinxi (五禽戏)	Wei, Cheng, and He (2015)/China, Bozhou (28)	RCT	Stable COPD	48	Wuquinxi	PFT (FEV_1_%, FEV_1_/FVC)	n.r
			24	GOLD 1–2	45	Usual care		
		Zhao (2015)/China, Zhengzhou (29)	RCT	Stable COPD	30	Wuquinxi	PFT (FEV_1_, FEV_1_%, FEV_1_/FVC), 6MWD, Borg scale, SGRQ	n.r
			12	GOLD 2 (estimated)	30	Usual care		
		Yao (2021)/China, Zhangjiajie (30)	RCT	Stable COPD	31	Wuquinxi	PFT (FEV_1_, FEV_1_%, FEV_1_/FVC), 6MWD, CAT, mMRC dyspnea scale	n.r
			12	GOLD 2	33	Usual care		
	Liuzijue (六字诀)	Jiang (2017)/China, Changsha (31)	RCT	Stable COPD	32	Liuzijue	PFT (FEV_1_, FEV_1_%, FVC, FEV_1_/FVC), 6MWD, CAT	n.r
			12	GOLD 1–2	33	Pursed-lip breathing		
					30	Enhanced liuzijue		
		Hou, and Cui (2017)/China, Xianyang (32)	RCT	Stable COPD	50	Simplified liuzijue	PFT (FEV_1_, FEV_1_/FVC), ABGA (PaO_2_, PaCO_2_), SGRQ	n.r
			24	GOLD 2–3	49	Usual care		
		Zhang, Chen, Zheng, LI, Zheng, and Ge (2009)/China, Fujian (33)	RCT	Stable COPD	21	Liuzijue	6MWD	n.r
			12	n.r	19	Usual care		
		Wu, Liu, Liu, Li al, and Wang (2018)/China, Shanghai (a) (34)	Single-blind RCT	Stable COPD	15	Liuzijue on land	PFT (FEV_1_%, FEV_1_/FVC), respiratory muscle strength(PE_max_, PI_max_), CON-TREX isokinetic muscle function test (elbow extension, elbow flexion, knee extension, and knee flexion)	n.r
			12	GOLD 1–4	16	Usual care		
					14	Liuzijue in the water		
		Zhu (2011)/China, Nanjing (35)	RCT	Stable COPD	20	Liuzijue	PFT (FEV_1_, FEV_1_%, FEV_1_/FVC)	n.r
			12	GOLD 2 (estimated)	22	Usual care		
					19	Walking exercise		
			Single-blind RCT	Stable COPD	59	Liuzijue	6MWD, airway resistance, specific airway conductance, respiratory muscle strength (PE_max_, PI_max_), monitored functional task evaluation, SF-36, CRQ	n.r
		Xiao, and Zhuang (2015)/China, Beijing (36)	24	GOLD 3 (estimated)	60	Pursed-lip breathing and walking exercise		
		Wu, Liu, Li, Li, and Wang (2018)/China, Shanghai(b) (37)	Single-blind RCT	Stable COPD	16	Liuzijue	PFT (FEV_1_, FEV_1_%, FVC%, FEV_1_/FVC, MMEF_25-75%_), 6MWD, 30-s sit-up test, Handgrip Strength Test, SGRQ	n.r
			24	GOLD 2–3	17	Usual care		
					17	Liuzijue and elastic band exercise		
		Li (Li, 2018)/China, Shanghai (38)	Single-blind RCT	Stable COPD	17	Liuzijue	PFT (FEV_1_%, FEV_1_/FVC), 6MWD, 30-s sit-up test, SGRQ	n.r
			24	GOLD 2–3	19	Usual care		
		Chen, Zhang, Zheng, Zheng, Li, and Ge (2008)/China, Fujian (39)	RCT	Stable COPD	21	Liuzijue	PFT (FEV_1_, FEV_1_%, FEV_1_/FVC), mMRC dyspnea scale	n.r
			12	GOLD 3 (estimated)	19	Usual care		
		Yan (2020)/China, Taian (40)	RCT	Stable COPD	30	Enhanced Liuzijue	TER, PFT (FEV_1_%, FEV_1_%pred), 6MWD	n.r
			12	GOLD 2–3	28	Usual care		
					29	Liuzijue and herbal medicine		
					30	Enhanced Liuzijue and herbal medicine		
		Deng, Chen, Chen, Fan, Zhou, and Chen (2020)/China, Fuzhou (41)	RCT	Stable COPD	30	Liuzijue	PFT (FEV_1_, FEV_1_%, FEV_1_/FVC), immune cytokines (TNF-α, IL-8), CAT	n.r
			12	GOLD 2–4	32	Usual care		
		Deng, Zhang, and Chen (2018)/China, Fuzhou (42)	RCT	Stable COPD	28	Liuzijue	mMRC dyspnea scale, 6MWD, SGRQ	n.r
			12	GOLD 2–3	26	Full-body breathing		
		Deng (2009)/China, Fuzhou (43)	RCT	Stable COPD	31	Enhanced Liuzijue	PFT (FEV_1_, FEV_1_%, FEV_1_/FVC), 6MWD, mMRC dyspnea scale, BMI, BODE, SGRQ	n.r
			12	GOLD 2–3	29	Full-body breathing		
		He (2019)/China, Beijing (44)	RCT	Stable COPD	30	Liuzijue	Number of AECOPD, CAT, SGRQ, PFT (FEV_1_%, FVC, FEV_1_/FVC), immunocytes (CD3 + (%), CD4 + (%), CD8 + (%), CD4+/CD8+)	n.r
			24	GOLD 1–3	30	Usual care		
		Lan, Han, Wang, Deng, Liu, and Feng (2016)/China, Luzhou (45)	RCT	Stable COPD	42	Liuzijue	CAT, PFT (FEV_1_%, FEV_1_/FVC)	n.r
			12	GOLD 2 (estimated)	42	Usual care		
		Zhang (2021)/China, Tianjin (46)	RCT (non-blind)	Stable COPD	29	Liuzijue	6MWD, Borg scale, pulse rate, SpO_2_, Brief-BESTest, BMI, middle arm circumference	n.r
			12	GOLD 1–4	30	Usual care		
		Lu, and Wang (2021)/China, Fujian province (47)	RCT	Stable COPD	135	Liuzijue	Number of AECOPD, PFT (FEV_1_, FVC), BODE, CAT, TER, clinical symptom score	n.r
			12	GOLD 2–3	139	Usual care		
		Liu (2017)/China, Shanghai (48)	RCT	Stable COPD	17	Liuzijue on land	Respiratory muscle strength test (MIP, MEP), 6MWD, CON-TREX Isokinetic Strength Test (peak torque)	n.r
			12	GOLD 1–4	19	Usual care		
					14	Liuzijue in the water		
		Hu, Gui, Tu, Yang, Wang, and Ji (2021)/China, Shanghai (49)	RCT	Stable COPD	18	Liuzijue on land	PFT (FEV_1_, FEV_1_%, FEV_1_/FVC), airway resistance, 6MWD, CAT	n.r
			24	GOLD 2–3	20	Usual care		
					18	Liuzijue in the water		
		Shen (2017)/China, Shanghai (50)	RCT	Stable COPD	45	Liuzijue	CAT, PFT (FEV_1_, FVC, FEV_1_/FVC)	n.r
			24	GOLD 1–2	47	Usual care		
		Ji, Luo, Shi, Yang, and Wang (2019)/China, Shanghai(51)	RCT	Stable COPD	28	Liuzijue	Airway resistance (R5_HZ_), PFT (FEV_1_, FVC, FEV_1_/FVC), mMRC dyspnea scale	n.r
			12	GOLD 3 (estimated)	29	Usual care		
		Qu, Huang, and Lu (2019)/China, Taizhou (52)	RCT	Stable COPD	52	Liuzijue	6MWD, mMRC dyspnea scale, SGRQ	n.r
			24	n.r	52	Full-body breathing		
		Li (2011)/China, Fuzhou (53)	RCT	Stable COPD	30	Liuzijue	PFT (FEV_1_, FEV_1_%, FEV_1_/FVC), airway resistance (Raw%, sGaw%), respiratory muscle strength (MIP, MEP)	n.r
			12	GOLD 2–3	30	Full-body breathing		
		Li (2018)/China, Beijing (54)	RCT	Stable COPD	15	Liuzijue	PFT (FEV_1_, FVC, FEV_1_/FVC), CAT	n.r
			12	GOLD 1–3	15	Usual care		
		Yan (2021)/China, Lanzhou (55)	RCT	Stable COPD	17	Liuzijue	PFT (FEV_1_%, FEV_1_/FVC), 6MWD, 30-s sit-up test, SGRQ	n.r
			24	GOLD 1–3	19	Usual care		
		Wang, Yang, and Tang (2013) /China, Shanghai (56)	RCT	Stable COPD	39	Enhanced Liuzijue	Number of AECOPD, PFT (FEV_1_%, FEV_1_/FVC)	n.r
			52	GOLD 2–4	41	Diaphragmatic and pursed-lip breathing		
		Quan (2021)/China, Tianjin (57)	RCT	Stable COPD	30	Enhanced Liuzijue	PFT (FEV_1_, FEV_1_/FVC), CAT, mMRC dyspnea scale, TER, clinical symptom score	n.r
			12	GOLD 2–3	30	Usual care		
					30	Diaphragmatic and pursed-lip breathing		
		Liu, Wu, Li, Li, Wang, and Shan (2021)/China, Shanghai (58)	RCT	Stable COPD	15	Liuzijue on land	Peak exercise capacity (Peak VO_2_, Relative peak VO_2_, Peak VE, Pead W, AT), 6MWD, 30-s sit-up test, SGRQ	0 “No adverse events occurred in any of the groups during the intervention.”
			12	GOLD 1–4	16	Usual care		
					14	Liuzijue in the water		
	Yijinging (易筋经)	Zhang, Xv, Luo, Meng, and Ji (2016)/China, Jiangsu province (59)	Single-blind RCT	Stable COPD	42	Yijinging	6MWD, CAT, Regulatory emotion Self-Efficacy questionnaire, PFT (FEV_1_, FEV_1_%, FEV_1_/FVC)	n.r
			24	GOLD 1–3	45	Usual care		
					43	Self-management exercise intervention		
		Gao (2015)/China, Suzhou (60)	RCT	Stable COPD	55	Yijinjing	PFT (FEV_1_, FEV_1_%, FEV_1_/FVC), 6MWD, CAT, ESCA (exercise of self-care agency)	n.r
			24	GOLD 0–4	57	Diaphragmatic breathing		
	Tai chi (太极)	Zhu, Shi, Yan, He, Wang, Yi, and Huang (2018)/China, Changsha (61)	Single-blind RCT	Stable COPD	30	Tai chi	6MWD, mMRC dyspnea scale, CAT	n.r
			12	GOLD 2–4	30	Usual care		
		Zhang, Cai, Zhang, Chen, Jia, Zhang, and Wang (2014)/China, Jinan (62)	RCT	Stable COPD	18	Tai chi	PFT (FEV_1_, FEV_1_%, FEV_1_/FVC), 6MWD, CAT, number of AECOPD	n.r
			52	GOLD 1–3	18	Usual care		
					18	Diaphragmatic breathing		
					18	Tai chi + diaphragmatic breathing		
		Zhang, Wu, and Wang (2012)/China, Beijing (63)	RCT	Stable COPD	30	Tai chi + pursed-lip breathing	PFT (FEV_1_%, FEV_1_/FVC), 6MWD, SGRQ	n.r
			52	GOLD 1–3	30	Usual care		
					30	Pursed-lip breathing		
		Yeh, Litrownik, Wayne, Beach, Klings, Reyes Nieva, Pinheiro, Davis, and Moy (2020)/USA, Boston (64)	RCT	Stable COPD	52	Tai chi	HRQL (CRQ, UCSDSOB), CSES, CES-D, Multidimensional Scale of Perceived Social Support, 6MWD, Chair Stand test, Chair sit and reach test, PROMIS Fatigue Short Form 7a, CHAMPS	19 “A total of 19 reportable adverse events occurred during the 12- week intervention period (16 in TC, 3 in education).” [9 COPD exacerbations, 4 musculoskeletal flares]
			12	GOLD 2–4	29	Usual care		
		Wang, Mo, Cheng, Liu, and Hu (2014)/China, Yingde & Zhengzhou (65)	RCT	Stable COPD	11	Tai chi	SGRQ, BODE	n.r
			8	GOLD 2–3	11	Usual care		
		Wang, Wu, Chen, and Liu (2019)/China, Zunyi (66)	Single-blind RCT	Stable COPD	26	Tai chi	PFT (FEV_1_, FEV_1_%, FVC), 6MWD, CAT	0 “No adverse events related to intervention during the Tai Chi training, which is consistent with an earlier report.”
			12	GOLD 2–4	24	Usual care		
		Polkey, Qiu, Zhou, Zhu, Wu, Chen, Ye, He, Jiang, He, Mehta, Zhong, and Luo (2018)/China, Meizhou (67)	RCT	Stable COPD	55	Tai chi	SGRQ, PFT (FEV_1_%), 6MWD	0 “No difference in adverse events was observed between the groups.”
			12	GOLD 3 (estimated)	55	PR		
		Niu, He, Luo, and Hu (2014)/China, Changsha (68)	Single-blind RCT	Stable COPD	20	Tai chi: Tai chi + pursed-lip breathing and walking exercise	PFT (FEV_1_, FEV_1_%), 6MWD, blood gas parameters (PaO_2_, PaCO_2_), diaphragm strength parameters (TwPes, TwPga, TwPdi)	0 “However, in this study, we did not observe serious adverse events.”
			24	GOLD 2–3	19	Usual care		
		Ng, Chiang, Tang, Siu, Fung, Lee, and Tam (2014)/China, Hong Kong (69)	RCT	Stable COPD	94	Tai chi	CSES, self-efficacy for managing shortness of breath, SGRQ-HKC (Hong Kong Chinese version), 6MWD, PFT (FEV_1_, FEV, FEV_1_/FVC)	0 “There were no adverse events reported in both control and intervention groups.”
			6	GOLD 1–4	98	PR		
		Yeh, Roberts, Wayne, Davis, Quilty, and Phillips (2010)/USA, Boston (70)	RCT	Stable COPD	5	(G1) Tai chi	CRQ, 6MWD, Peak oxygen uptake, Exercise duration, PFT (FEV_1_/FVC, FRC), Timed Up-And-Go assessment, CSES, CHAMPS	0 “No adverse events occurred during the class sessions. No patients in either group were hospitalized during the study period for COPD exacerbation, and there were no deaths.”
			12	GOLD G1 2.4 ± 0.5 G2 2.6 ± 0.5	5	(G2) Usual care		
		Moy, Wayne, Litrownik, Beach, Klings, Davis, Pinheiro, and Yeh (2021)/USA, Boston (71)	RCT	Stable COPD	34	Tai chi	6MWD, CRQ, UCSDSOB, CES-D, Perceived Stress Scale, Multidimensional Scale of Perceived Social Support, CSES, CHAMPS	0 “There was no difference in adherence and adverse events between groups.”
			24	GOLD 1–4	35	Usual care		
		Kantatong, Panpanich, Deesomchok, Sungkarat, and Siviroj (2020)/Thailand, Chiang Mai (72)	RCT	Stable COPD	25	Tai chi	6MWD, PFT (FEV_1_, FVC), mMRC dyspnea scale, SGRQ	n.r
			24	GOLD 1–2	25	Usual care		
		Du, Ding, Wang, Yang, Xing, Liu, and Zhu (2013)/China, Zhangjiakou (73)	RCT	Stable COPD	36	Tai chi	PFT (FEV_1_%, FEV_1_/FVC), MVV, SaO_2_%, 6MWD, CAT	n.r
			12	GOLD 1–2	38	Usual care		
					38	Diaphragmatic, pursed-lip breathing and walking exercise		
		Chan, Lee, Lee, Suen, and Tam, Chair, and Griffiths (2013)/China, Hong Kong (74)	RCT	stable COPD	70	Tai chi	PFT (FEV_1_, FVC), 6MWD, Borg scale, number of AECOPD	n.r
			24		67	Usual care		
				n.r	69	Diaphragmatic and pursed-lip breathing + Walking exercise		
		Zhang, and Liu (2019)/China, Chongqing (75)	RCT	Stable COPD	29	Tai chi	PFT (FEV_1_, FEV_1_%, FVC), CAT	n.r
			104	GOLD 2–3	29	Pursed-lip breathing		
		Cui, Xing, Yang, and Li (2016)/China, Zhangjiakou (76)	RCT	Stable COPD	n.r	Tai chi + non-invasive ventilators	PFT (FEV_1_, FEV_1_%, FEV_1_/FVC), ABGA (PaO_2_, PaCO_2_), CAT, 6MWD, SGRQ	n.r
			52	GOLD 1–3		Non-invasive ventilators		
		Li (Li, 2019)/China, Fenyang (77)	RCT	Stable COPD	26	Tai chi + diaphragmatic and pursed-lip breathing	PFT (FEV_1_, FVC, FEV_1_/FVC), SCL-90(症状自评量表), SGRQ	n.r
			12	n.r	23	Diaphragmatic and pursed-lip breathing		
		Li, Li, Sun, and Wang (2016)/China, Yantai (78)	RCT	Stable COPD	20	Tai chi + diaphragmatic and pursed-lip breathing	PFT (FEV_1_, FEV_1_%, FEV_1_/FVC), 6MWD, immune cytokines (TNF-α, IL-8, IL-6)	n.r
			12	GOLD 3 (estimated)	20	Diaphragmatic and pursed-lip breathing		
		Li, Fang, and Liu (2012)/China, Jinan (79)	RCT	Stable COPD	30	Tai chi + diaphragmatic and pursed-lip breathing	PFT (FEV_1_, FVC, FEV_1_/FVC), SGRQ, SCL-90(症状自评量表), bone mineral density	n.r
			24	GOLD 2–3	30	Diaphragmatic and pursed-lip breathing		
		Pan, Wang, Min, Xiao, Huang, Mao, Peng, and Wang (2018)/China, Chengdu (80)	RCT	Stable COPD	20	Tai chi + health education	PFT (FEV_1_, FEV_1_%, FVC, FVC%), SGRQ, 6MWD, CAT, HAD	n.r
			8	GOLD 2 (estimated)	21	Usual care		
		Ren, Zhang, Hou, Yang, Qian, Wang, Li, Bian, Liu, Wang, and Ding (2017)/China, Beijing (81)	RCT	Stable COPD	30	Tai chi + health education	CPET, 6MWD, PFT (FEV_1_, FVC, FEV_1_/FVC, MMEF, MVV), CAT, mMRC dyspnea scale, SAS(self-rating anxiety scale), SDS(self-rating depression scale)	n.r
			12	GOLD 2–3	30	PR+ health education		
		He (He, 2019a; He, 2019b)/China, Jinan (82)	RCT	Stable COPD	39	Tai chi + health education	CAT, mMRC score, PFT (FEV_1_, FEV_1_%, FVC), 6MWD, BMI, BODE	n.r
			12	GOLD 1–3	37	Usual care		
	Baduanjin (八段锦)	Lu, Li, Zhang, Guo, and Liu (2015)/China, Beijing (83)	RCT	Stable COPD	80	Baduanjin	mMRC dyspnea scale, CAT, 6MWD	n.r
			2	n.r	80	Usual care		
		Feng, Pan, Wen, Che, and Jiao (2009)/China, Guangzhou (84)	RCT	Stable COPD	30	Baduanjin	PFT (FEV_1_, FEV_1_%, FVC, FEV_1_/FVC), ABGA (pH, PaO_2_, PaCO_2_), 6MWD	n.r
			24	GOLD 2 (estimated)	30	Usual care		
		Huang, Yao, and Zhu (2017)/China, Nanjing (85)	RCT	Stable COPD	31	Baduanjin	TER, PFT (FEV_1_, FEV_1_%, FEV_1_/FVC), CAT	n.r
			24	GOLD 2–3 (estimated)	31	Usual care		
		Yin (2013)/China, Guangzhou (86)	RCT	Stable COPD	10	Baduanjin	SGRQ, 6MWD, COPD-Quality Of Life, number of AECOPD and rehospitalizations	0 “Both groups maintained routine inhalation and oral medication during the intervention. No acute cardio-cerebrovascular events, no training-induced falls, fractures and other adverse events occurred.”
			24	GOLD 2–3	12	Diaphragmatic breathing		
		Chen, Deng, Chen, Zhang, and Deng (2015)/China, Fuzhou (87)	RCT	Stable COPD	31	Baduanjin + health education	CAT, SGRQ, 6MWD, clinical symptom score	n.r
			12	GOLD 2–4	30	Usual care		
		Zhu, and Chen (2014)/China, Changsha (88)	RCT	Stable COPD	63	Baduanjin	PFT (FEV_1_, FVC, FEV_1_%, FEV_1_/FVC), 6MWD	n.r
			24	GOLD 2–3	60	Usual care		
		Ng, Tsang, Jones, So, and Mok (2011)/China, Hong Kong (89)	single-blind RCT	Stable COPD	23	Baduanjin	6MWD, Monitored Functional Task Evaluation, SF-36, Chinese Chronic Respiratory Questionnaire	n.r
			24	GOLD 3 (estimated)	29	Pursed-lip breathing + walking exercise		
		Xu, Wang, Li, Zhu, and Wang (2010)/China, Yunyang (90)	RCT	Stable COPD	20	Baduanjin	CRQ, 6MWD, Borg scale	n.r
			52	GOLD 1–2	20	Usual care		
					20	PR		
					20	Baduanjin + PR		
		Huang, and Gao (2016)/China, Dongguan (91)	RCT	Stable COPD	31	Baduanjin + diaphragmatic and pursed-lip breathing	PFT (FEV_1_, FEV_1_%, FVC, FEV_1_/FVC), 6MWD	n.r
			12	GOLD 3–4	30	Diaphragmatic and pursed-lip breathing		
		Chen (2017)/China, Shenyang (92)	RCT	Stable COPD	60	Baduanjin + health education	PFT (FEV_1_, FVC), BDI (baseline dyspnea index), SF-36	n.r
			24	GOLD 2	60	Usual care		
		Chen, Liu, Li, Zhang, Zhou, Yang, and Chen (2015)/China, Chengdu (93)	RCT	Stable COPD	117	Baduanjin	PFT (FEV_1_, FEV_1_%, FVC, FEV_1_/FVC)	n.r
			12	GOLD 1–3	115	Usual care		
		Zheng (2019)/China, Guangzhou (94)	RCT	Stable COPD	16	Baduanjin	CAT, mMRC dyspnea scale, 6MWD, BODE, BMI, PFT (FEV_1_, FEV_1_%, PEF), respiratory muscle function test (PImax, PEmax)	1 “One patient in Baduanjin group had mild chest tightness, which relieved itself after a few min of rest. There were no adverse reactions in the other two groups.”
			12	GOLD 3–4	15	Usual care		
					11	Pursed-lip breathing		
		Deng (2014)/China, Fuzhou (95)	RCT	Stable COPD	31	Baduanjin + health education	CAT, 6MWD, PFT (FEV_1_, FEV_1_%, FVC, FEV_1_/FVC), mMRC dyspnea scale, clinical symptom score	n.r
			12	GOLD 2–4	30	Usual care		
		Deng, Yang, Dong, and Zhang (2020)/China, Guangzhou (96)	RCT	Stable COPD	27	Baduanjin	SGRQ, 6MWD, PFT (FEV_1_, FEV_1_%, FVC, FEV_1_/FVC), number of AECOPD	n.r
			24	GOLD 1–4 (B, C, D group)	27	Usual care		
		Zhang, Wang, Shi, Zou, Zhu, Sun, Zhang, Liu, and Yang (2017)/China, Changchun (97)	RCT	Stable COPD	30	Baduanjin	Thickness of deltoid fold, BMI, 6MWD, PFT (FEV_1_, FEV_1_%, FVC%)	n.r
			8	GOLD 2–3	30	Usual care		
		Sun (2014)/China, Changchun (98)	RCT	Stable COPD	40	Baduanjin	PFT (FEV_1_%), 6MWD, CAT	n.r
			52	GOLD 2	40	Usual care		
		Liu, and Chen (2013)/China, Chengdu (99)	RCT	Stable COPD	40	Baduanjin	6MWD, PFT (FEV_1_%, FEV_1_/FVC), mMRC dyspnea scale	n.r
			12	GOLD 1–4	40	Usual care		
		Ye (2016)/China, Changsha (100)	RCT	Stable COPD	40	Baduanjin	6MWD, SF-36	n.r
			4	n.r	40	Usual care		
		Guo, Gao, Xie, Fang, and Chen (2016)/China, Qingdao (101)	RCT	Stable COPD	30	Baduanjin	TER, PFT (FEV_1_, FEV_1_%, FVC, FEV_1_/ FCV), ABGA (pH, PaO2, PaCO_2_)	n.r
			24	GOLD 2 (estimated)	30	Usual care		
		Liang (2016)/China, Dongguan (102)	RCT	Stable COPD	41	Baduanjin	PFT (FEV_1_, FEV_1_%, FVC, FEV_1_/FVC), rehabilitation effects	n.r
			12	GOLD 3 (estimated)	41	Usual care		
		Pan, Luo, and Yang (2016)/China, Dazhou (103)	RCT	Stable COPD	42	Baduanjin	PFT (FEV_1_, FVC, FEV_1_%, FEV_1_/FVC), 6MWD, SGRQ	n.r
			24	GOLD 1–4	42	Usual care		
		Pan (2019)/China, Foshan (104)	RCT	Stable COPD	32	Baduanjin + PR	6MWD, CAT, SGRQ, mMRC dyspnea scale, Borg scale, Beck anxiety index, Beck depression index	n.r
			12	GOLD 2–4	32	Usual care		
					32	PR		
		Xue, Feng, Yao, Qi, and Wang al (2015)/China, Beijing (105)	RCT	Stable COPD	31	Baduanjin	PFT (FEV_1_, FEV_1_%, FVC, FEV_1_/FVC), CAT, number of AECOPD	n.r
			24	n.r	28	Usual care		
		Yu (2019)/China, Shiyan (106)	RCT	Stable COPD	41	Baduanjin	TER, PFT (FEV_1_, FEV_1_%, FVC, FVC%)	n.r
			12	GOLD 2 (estimated)	41	Usual care		
		Wang, and Fang (2018)/China, Dalian (107)	RCT	Stable COPD	37	Baduanjin + health education	PFT (FEV_1_, FEV_1_%, FVC, FEV_1_/FVC), 6MWD, SGRQ, number of AECOPD	n.r
			12	n.r	36	Usual care		
		Wang, Wang, and Zheng (2022)/China, Hangzhou (108)	RCT	Stable COPD	40	Baduanjin + PR	PFT (FEV_1_,FVC, FEV_1_/FVC), 6MWD, mMRC dyspnea scale, SGRQ	n.r
			12	n.r	40	PR		
		Yu (Yu, 2019)/China, Wuxi (109)	RCT	Stable COPD	80	Baduanjin + health education	mMRC dyspnea scale, 6MWD, TER	n.r
			24	GOLD 1–4	80	Usual care		
		Cao, Guo, Chen, Yan, and Zhang (2016)/China, Nanjing (110)	RCT	Stable COPD	52	Baduanjin + health education	Self-Rating Anxiety Scale, Self-Rating Depression Scale, PFT (FEV_1_, FEV_1_%, FEV_1_/FVC)	n.r
			24	GOLD 2–3	50	Walking exercise + health education		
Moxibustion	Moxibustion - unspecified	Wang, Qiu, Huang, and Chen (2016)/China, Nanning (111)	RCT	Stable COPD	35	Moxibustion	TER, clinical symptom score, PFT (FEV_1_%, FEV_1_/FVC)	n.r
			6	GOLD 3 (estimated)	35	Usual care		
		Li (Li, 2011)/China, Guangzhou (112)	RCT	Stable COPD	44	Moxibustion	PFT (FEV_1_%, FEV_1_/FVC), mMRC dyspnea scale, 6MWD, BMI	n.r
			12	GOLD 2–3	40	Usual care		
	Ginger moxibustion (隔姜灸)	He, He, and Mai (2013)/China, Shanghai (113)	RCT	Stable COPD	46	Ginger moxibustion	Clinical symptom score, PFT (FEV_1_, FEV_1_%, FVC, FEV_1_/FVC)	n.r
			6	GOLD 3 (estimated)	47	Usual care		
		Cui, Wang, Han, Hou, Wang, and Cao (2017)/China, Changzhi (114)	RCT	Stable COPD	15	Ginger moxibustion	TER, PFT (FEV_1_, FVC), CAT	n.r
			14	n.r	15	Usual care		
					15	Ginger moxibustion		
	Heat-sensitive point’s moxibustion (热敏灸)	Liang (2018)/China, Foshan (115)	RCT	Stable COPD	44	Heat-sensitive points’ moxibustion	PFT (FEV_1_, FEV_1_/FVC, PEF (peak expiratory flow), FEF_25%_, FEF_50%_, FEF_75%_, TNF-α, HMGB1	n.r
			8	GOLD 2–3	44	Usual care		
		Chen (Chen, 2017)/China, Nanchang (116)	Single-blind RCT	Stable COPD	30	(G1) Heat-sensitive points’ moxibustion	TER, clinical symptom score, PFT (FEV_1_%, FEV/FVC), ABGA(PaO_2_, PaCO_2_)	n.r
					30	(G1) Usual care		
			12	GOLD G1 1–2 G2 3	30	(G2) Heat-sensitive points’ moxibustion		
					30	(G2) Usual care		
			RCT	Stable COPD	40	Heat-sensitive points’ moxibustion	CAT, 6MWD, PFT (FEV_1_%, FEV_1_/FVC), immune cytokines (TNF-*α*, IL-8, IL-6, IgA, IgM, IgG, CRP), number of AECOPD, TER	n.r
		Fan, Lu, Chen, Zhang, Pan, and Zhou (2021)/China, Haikou (117)	24	GOLD 2 (estimated)	40	Usual care		
		Wang (2011)/China, Zhengzhou (118)	RCT	Stable COPD	60	Heat-sensitive point’s moxibustion	Clinical symptom score, PFT (FEV_1_%, FEV_1_/FVC), ABGA (pH, PaO2, PaCO2)	n.r
			12	GOLD 1–3	60	Usual care		
		Zhe, Xue, and Ni (2017)/China, Yan’an (119)	RCT	Stable COPD	40	Heat-sensitive point’s moxibustion + pursed-lip breathing	PFT (FEV_1_, FEV_1_%, FEV_1_/FVC), SGRQ	n.r
			12	GOLD 1–3	40	Usual care + pursed-lip breathing		
		Cheng, and Shu (2011)/China, Jiujiang (120)	RCT	Stable COPD	30	Heat-sensitive points’ moxibustion	PFT (FEV_1_, FEV_1_%, FEV_1_/FVC), TER	n.r
			4	GOLD 2 (estimated)	30	Usual care		
					30	Traditional moxibustion		
	Yi Fei moxibustion (益肺灸)	Zhao, Huang, and Zhu (2018)/China, Nanjing (121)	RCT	Stable COPD	24	Yi Fei moxibustion	TER, PFT (FEV_1_, FEV_1_%, FEV_1_/FVC), number of AECOPD, immunocytes (CD4+, CD8+)	n.r
			24	GOLD 2 (estimated)	24	Usual care		
		Li (Li, 2015)/China, Zhengzhou (122)	RCT	Stable COPD	20	Yi Fei moxibustion	Number of AECOPD, PFT (FEV_1_, FEV_1_%, FEV_1_/FVC), mMRC dyspnea scale, the clinical symptom score, 6MWD, CAT, immune function (CD3, CD4/CD8, IgG, IgM)	n.r
			12	GOLD 1–4	27	Usual care		
		Huang (2021)/China, Zhengzhou (123)	RCT	Stable COPD	63	Yi Fei moxibustion	6MWD, clinical symptom score, PFT (FEV_1_, FVC, FEV_1_/FVC), SGRQ	n.r
			12	GOLD 1–3	63	PR		
		Han (2017)/China, Yue Pu Hu Xian (124)	RCT	Stable COPD	27	Yi Fei moxibustion	ABGA (PaO_2_, PaCO_2_), PFT (FEV_1_%, FEV_1_/FVC)	n.r
			6–12	GOLD 1–4	33	Usual care		
		Qian (2014)/China, Zhengzhou (125)	RCT	Stable COPD	30	Yi Fei moxibustion	TER, PFT (FEV_1_, FEV_1_%), 6MWD, mMRC dyspnea scale, immunocytes (CD3+, CD4+, CD8+, CD4+/CD8+)	n.r
			12	GOLD 1–2	30	Usual care		
		Yang (Yang, 2016)/China, Puyang (126)	RCT	Stable COPD	30	Yi Fei moxibustion	PFT (FEV_1_%, FEV_1_/FVC), TER	n.r
			12	GOLD 2 (estimated)	30	Usual care		
		Cui, and Liang (2015)/China, Zhengzhou (127)	RCT	Stable COPD	30	Yi Fei moxibustion	PFT (FEV_1_%, FEV_1_/FVC), ABGA (PaO_2_, PaCO_2_), clinical symptom score	n.r
			12	GOLD 2 (estimated)	30	Usual care		
		Zhou, and Yang (2011)/China, Zhengzhou (128)	RCT	Stable COPD	108	Yi Fei Moxibustion	TER, clinical symptom score, PFT (FEV_1_, FEV_1_%, FVC, PEF)	n.r
			12	GOLD 2 (estimated)	102	Usual care		
		Zhang, and Zhong (2012) /China, Zhengzhou (129)	RCT	Stable COPD	45	Yi Fei moxibustion	Clinical symptom score, PFT (FEV_1_, FEV_1_%)	n.r
			12	GOLD 1–2	44	Usual care		
	Fire-dragon moxibustion (火龙灸)	Xu, Wu, Liu, Chen, Lin, and Zhang (2022)/China, Guangzhou (130)	RCT	Stable COPD	44	Fire-dragon moxibustion	PFT (FEV_1_%, FVC%, FEV_1_/FVC), mMRC dyspnea scale, CCQ, CAT, BODE	n.r
			4	GOLD 2–3 (estimated)	47	Usual care		
Chuna		Rocha, Souza, Brandão, Rattes, Ribeiro, Campos, Aliverti, and deAndrade (2015)/Brazil (131)	RCT	Stable COPD	10	Manual diaphragm release technique	Diaphragm mobility, compartmental chest wall volume, 6MWD, respiratory muscle strength	n.r
			2	GOLD 3 (estimated)	9	Sham		
		Noll, Degenhardt, Johnson, and Burt (2008)/United States (132)	RCT	Stable COPD	18	Osteopathic manipulative treatment (OMT)	PFT (FEV_1_, FVC, FEV_1_/FVC, FEF_25%_, FEF_50%_, FEF_75%_, FEF_25-75%_, FEF_Max_, FIVC, FIF_50%_, FIF_Max_, ERV, IC, MVV, SVC, TGV, RV, TLC, RV/TLC), airway resistance, airway conductance	6 “None of the reported side effects in either study group were judged to be severe. (6 cases just like muscle soreness).”
			<1	GOLD 3 (estimated)	17	Sham		
		Maskey-Warzechowska, Mierzejewski, Gorska, Golowicz, Jesien, and Krenke (2019)/Poland (133)	Cross-over RCT (wash-out 2 wks)	Stable COPD	19	OMT	PFT (FEV_1_, FEV_1_%, FVC, FVC%, FEV_1_/FVC, TLC, TLC%, RV, RV%, RV/TLC, Raw (airway resistance), Raw%, IC, IC%, FRC, FRC%), DVAS	0 “No adverse effects associated with the OMT and sham intervention were observed in any of the participating patients.”
			<1	GOLD 3–4		Sham		
		Kurzaj, Wierzejski, Dor, Stawska, and Rożek (2013)/Poland (134)	RCT	Stable COPD	20	6 specialized physiotherapy + basic physiotherapy	BODE (FEV_1_, FEV_1_%, 6MWD, MRC, BMI)	n.r
			1	GOLD 2–3	10	basic physiotherapy		
		Buran Cirak, Yilmaz Yelvar, and Durustkan Elbasi (2022)/Turkey (135)	Single-blind RCT	Stable COPD	30	Manual therapy + Inspiratory muscle training (IMT)	PFT (FEV_1_%, FVC%, FEV_1_/FVC, PEF%, FEF_25-75%_), respiratory muscle strength (MIP, MIP%, MEP, MEP%), 6MWD, mMRC dyspnea scale, fatigue severity scale, SGRQ,	n.r
			12	GOLD 3–4	30	IMT		
		Chen, Zhong, Liu, Zhang, Xie, Jin, and Zhou (2006)/China, Shanghai (136)	RCT	Stable COPD	15	Chuna	PFT (FEV_1_, FEV_1_%, FVC), 6MWD, clinical symptom score	n.r
			8	GOLD 2	15	Usual care		
Acupuncture	Acupuncture (针刺)	Suzuki, Namura, Ohno, Tanaka, Egawa, Yokoyama, Akao, Fujiwara, and Yano (2008)/Japan (137)	RCT	Stable COPD	15	Acupuncture	Borg scale, 6MWD, SpO_2_, PFT (FEV_1_, FEV_1_%, FVC, FEV_1_/FVC, VC, FEV_25%_), ventilatory muscle strength and endurance (MIP, MEP), Fletcher Hugh-Jones category	3 “Three patients reported some minor transitory bruising and pain after acupuncture treatment, but there were no major adverse reactions.”
			10	GOLD 2–4	15	Usual care		
		Liu, Shi, Song, Zhang, and Jiang (2015)/China, Shanghai (138)	RCT	Stable COPD	40	Acupuncture	TER, clinical symptom score, 6MWD, PFT (FEV_1_%, FEV_1_/FVC)	n.r
			12	GOLD 3–4	40	Usual care		
		Deering, Fullen, Egan, McCormack, Kelly, Pender, and Costello (2011)/Ireland (139)	RCT	Stable COPD	16	Acupuncture + PR	Immune cytokines (IL-8, IL-6), PFT (FEV_1_%, FVC%, IVC%, Pi_Max_), free living physical activity, incremental shuttle walk test, SGRQ, EQ-5D	n.r
			7	GOLD 1–4	25	PR		
					19	Usual care		
		Suzuki, Muro, Ando, Omori, Shiota, Endo, Sato, Aihara, Matsumoto, Suzuki, Itotani, Ishitoko, Hara, Takemura, Ueda, Kagioka, Hirabayashi, Fukui, and Mishima (2012)/Japan (140)	Single-blind RCT	Stable COPD	30	Acupuncture	6MWD, PFT (FEV_1_, FEV_1_%, VC, IC, FVC, FRC, TLC, RV/TLC, D_Lco_, D_Lco_/VA), respiratory muscle strength (MEP, MIP), Borg scale, SpO_2_, SGRQ, mMRC dyspnea scale, BMI, ABGA (pH, PaO_2_, PaCO_2_), rib cage ROM, bicarbonate	22 “All events were minor reactions and patients recovered in a short time (fatigue, subcutaneous hemorrhage, dizziness, needle site pain). No serious events due to acupuncture treatment were reported.”
			12	GOLD 2–4	32	Sham		
		Li (Li, 2019)/China, Chengdu (141)	Single-blind RCT	Stable COPD	45	Acupuncture	Borg scale, 6MWD, PFT (FEV_1_, FEV_1_%, FVC), SpO_2_	0 “there were no adverse effects directly related to acupuncture treatment.”
			6	GOLD 2 (estimated)	46	Usual care		
		Feng, Wang, Li, Zhao, and Xu (2016) /China, Fuyang & Harbin (142)	RCT	Stable COPD	32	Acupuncture	6MWD, Borg scale, SpO_2_, SGRQ, PFT (FEV_1_%, FEV_1_/FVC)	n.r
			8	GOLD 2–4	32	Sham		
		Zhong (2018)/China, Maoming (143)	RCT	Stable COPD	30	Acupuncture	PFT (FEV_1_, FEV_1_/FVC), CAT, ABGA (PaO_2_, SPaO_2_, PaCO_2_)	n.r
			1	GOLD 2–3 (estimated)	30	Usual care		
		Deng, Zhang, and Wang (2016)/China, Guangzhou (144)	RCT	Stable COPD	22	Acupuncture	Number of AECOPD and hospitalizations, SpO_2_, PFT (FEV_1_%, FEV_1_/FVC), 6MWD	n.r
			24	n.r	22	Usual care		
		Yang, and Li (2018)/China, Wuhan (145)	RCT	Stable COPD	30	Acupuncture	PFT (FEV_1_, FEV_1_%, FEV_1_/FVC, PEF), clinical symptom score, SGRQ	n.r
			8	GOLD 3 (estimated)	30	Usual care		
		Jiao (2020)/China, Zhengzhou (146)	RCT	Stable COPD	30	Acupuncture + health education	TER, CAT, 6MWD, number of AECOPD	n.r
			12	n.r	29	Usual care		
		Tang (2017)/China, Fuzhou (147)	RCT	Stable COPD	30	Acupuncture	TER, clinical symptom score, 6MWD	0 “There were no significant abnormalities in the three major heart, liver and kidney function in the two groups.”
			8	n.r	29	Usual care		
		Li, and Liu (2020)/China, Shenyang (148)	RCT	Stable COPD	40	Acupuncture	PFT (FEV_1_, FVC, PEF), 6MWD, SGRQ, TER	n.r
			12	n.r	40	Usual care		
		Wang, and Wang (2021)/China, Chongqing (149)	RCT	Stable COPD	41	Acupuncture	TER, PFT (FEV_1_, FVC, FEV_1_/FVC, MVV, MIP), ABGA (pH, PaO_2_, PaCO_2_), immunocytes (CD4+, CD8+, CD4+/CD8+)	n.r
			6	GOLD 1–3	41	Usual care		
	Acu-TENS	Ngai, Jones, Hui-Chan, Ko, and Hui (2010)/China, Hong Kong (150)	RCT	Stable COPD	10	Acu-TENS	PFT (FEV_1_, FVC), DVAS, 6MWD, SGRQ, Blood test (IL-8, TNF-α, CRP, b-Endorphin)	0 “No adverse effect was reported during the study.”
			4	n.r	8	Placebo-TENS		
					10	Sham-TENS		
		Liu, Fan, Lan, Dong, Fu, and Mao (2015)/China, Chengdu(151)	Single-blind RCT	Stable COPD	25	Acu-TENS	PFT (FEV_1_%, FVC%), 6MWD, SpO_2_, CAT, DVAS	0 “During the trial, no adverse events occurred.”
			4	GOLD 2–4	25	Placebo-TENS		
		Lau, and Jones (2008)/China, Hong Kong (152)	RCT	Stable COPD	23	Acu-TENS	PFT (FEV_1_, FVC), DVAS	0 “There was no report of adverse effects.”
			<1	GOLD 1–2	23	Placebo-TENS		
		Jones, Ngai, Hui-Chan, and Yu (2011)/China, Sichuan province (153)	RCT	COPD	22	Acu-TENS	PFT (FEV_1_, FVC, PEFR), DVAS, respiratory rate, blood ß-endorphin levels	0 “No adverse effects associated with the study were reported.”
			<1	n.r	22	Placebo-TENS		
	Warm acupuncture (温针)	Yang (2016)/China, Xiamen (154)	RCT	Stable COPD	30	Warm acupuncture	TER, clinical symptom score, PFT (FEV_1_%, FEV_1_/FVC), CAT	0 “There were no significant abnormalities in the three major heart, liver and kidney function in the two groups.”
			4	GOLD 1–2	31	Usual care		
		Li (2015)/China, Xiamen (155)	RCT	Stable COPD	29	Warm acupuncture	TER, clinical symptom score, CAT, PFT (FEV_1_%, FEV_1_/FVC)	0 “There was no acute exacerbation of COPD, and no significant abnormalities in the three major heart, liver and kidney function in the two groups.”
			4	GOLD 1–2	30	Usual care		
		Shi, Ni, and Wang (2021)/China, Shanghai (156)	RCT	Stable COPD	49	Warm acupuncture	TER, clinical symptom score, heart function (left ventricular end diastolic diameter and left ventricular ejection fraction), 6MWD, PFT (FEV_1_, FEV_1_%, FEV_1_/FVC), CAT	0 “During treatment, no local bruise, burn, infection and other adverse events occurred in both groups.”
			8	GOLD 2 (estimated)	48	Usual care		
	Auricular acupressure (耳穴贴压)	Pang, and Lai (2014)/China, Weifang (157)	RCT	Stable COPD	24	Auricular acupressure	PFT (FEV_1_, FEV_1_%, FEV_1_/FVC), Nutrition (IBM%, Hb, serum albumin, prealbumin, TSF(头三肌皮皱厚度))	n.r
			12	GOLD 2–4	23	Usual care		
		Li, Hong, and Huang (2017)/China, Fuzhou (158)	RCT	Stable COPD	28	Auricular acupressure + health education	PFT (FEV_1_, FEV_1_%, FVC, FEV_1_/FVC), mMRC dyspnea scale	n.r
			24	GOLD 2 (estimated)	28	Usual care		
					26	Auricular acupressure of “Midnight-noon Ebb-flow”		
		Jin, Lu, and Liu (2009)/China, Wenzhou (159)	RCT	Stable COPD	30	Auricular acupressure	TER, SGRQ, PFT (FEV_1_, FEV_1_%, FVC, FEV_1_/FVC)	0 “There was no significant abnormality in the blood, urine, liver, kidney and electrocardiogram before and after treatment.”
			2	GOLD 3 (estimated)	30	Usual care		
	Electroacupuncture (电针)	He, Li, Zheng, Gao, Pan, Wang, Huang, Ge, Zhong, and Tong (2021)/China, Guangzhou (160)	Single-blind RCT	Stable COPD	30	Electroacupuncture + bicycle exercise	PFT (FEV_1_%, FVC%, FEV_1_/FVC, MVV%, MEF_25%_, MEF_50%_, MEF_75%_), CPET(METs%, VO_2_/kg%, VE%, VO_2_/HR, VEmax, VE/VO_2_, VE/VCO_2_), 6MWD, CAT, mMRC dyspnea scale	n.r
			5	GOLD 1–4	26	Placebo needling + bicycle exercise		
		Ge, Yao, Tong, He, Li, and Kong (2017)/China, Guangzhou (161)	RCT	Stable COPD	22	Electroacupuncture + bicycle exercise	BMI, 6MWD, PFT (FEV_1_%, FVC%, FEV_1_/FVC, MVV%), 平均运动里程, 平均最大心率	n.r
			4	GOLD 2–4	19	Placebo needling + bicycle exercise		
	Pressing needle (皮内针)	Chen (2018)/China, Deyang (162)	RCT	Stable COPD	50	Pressing needle + diaphragmatic and pursed-lip breathing	PFT (FEV_1_%, FVC, FEV_1_/FVC), Treatment satisfaction	n.r
			3	n.r	50	Diaphragmatic and pursed-lip breathing		
		Peng (2015)/China, Chengdu (163)	RCT	Stable COPD	29	Pressing needle + health education	TER, the clinical symptom score, CAT, PFT (FEV_1_%, FVC%, FEV_1_/FVC)	1 “In this study group, one patient showed mild swelling of the needle site during needle retention. After rest, it was relieved and relieved by itself. No other abnormalities were observed.”
			8	GOLD 1–3	30	Usual care		
		Wang, Wang, and Pang (2019)/China, Shanghai (164)	RCT	Stable COPD	48	Pressing needle	TER, the clinical symptom score, 6MWD, mMRC dyspnea scale, SGRQ	0 “There were no adverse events during the treatment.”
			12	GOLD 2–4	49	Usual care		
	Acupressure (穴位按揉)	Tuo, Nong, Guo, Wu, Lai, and Huang (2018)/China, Wuzhou (165)	RCT	Stable COPD	40	Acupressure	BMI, PFT (FEV_1_%), 6MWD, mMRC dyspnea scale, BODE, Number of rehospitalization	n.r
			52	GOLD 3 (estimated)	39	Usual care		

### 3.2 Quality/risk of bias of included studies

The studies had a low risk of bias in terms of random sequence generation (61.3%) (24–26, 31, 32, 34, 37, 38, 40–44, 46–48, 50, 52–54, 56–58, 60–64, 66–, 68–72, 78, 80–82, 85, 86, 88, 89, 92–100, 110, 117–119, 121, 125, 127, 128, 130–133, 135, 139–142, 145–147, 149–153, 155, 156, 158, 160, 161, 163–165) and incomplete outcome data (83.8%) (24, 25, 28, 30–38, 40–44, 46–52, 54–72, 74, 75, 78–81, 83–85, 87, 93, 95–139, 141–153, 156, 158, 159, 161, 162, 164, 165), but there was unclear risk of bias in terms of allocation concealment (80.9%) (26–33, 35, 36, 38–40, 42–45, 47, 49, 51–57, 59–67, 70, 73–85, 87, 88, 90–93, 96–117, 119–130, 132–134, 136–141, 143–149, 154–159, 161, 162, 164, 165) and blinding of the outcome assessment (78.2%) (27–33, 35, 39–45, 47–49, 51–57, 59–63, 65, 67, 69, 73–88, 90–94, 96–130, 133–136, 138, 143–149, 154–159, 161, 162, 164, 165). Because of the nature of non-pharmacological treatment, there were no studies with a low risk of bias in terms of blinding of participants and personnel, and 14.8% of the studies (25–27, 34, 36–38, 46, 66, 68, 89, 131, 133, 140–142, 150–153, 160) had a high risk of bias in this aspect. One study published the protocol (64), and the remaining studies had an unclear risk of bias in terms of selective reporting, as shown in Supplementary Table S2.

### 3.3 Intervention effects

**Figure 2:**
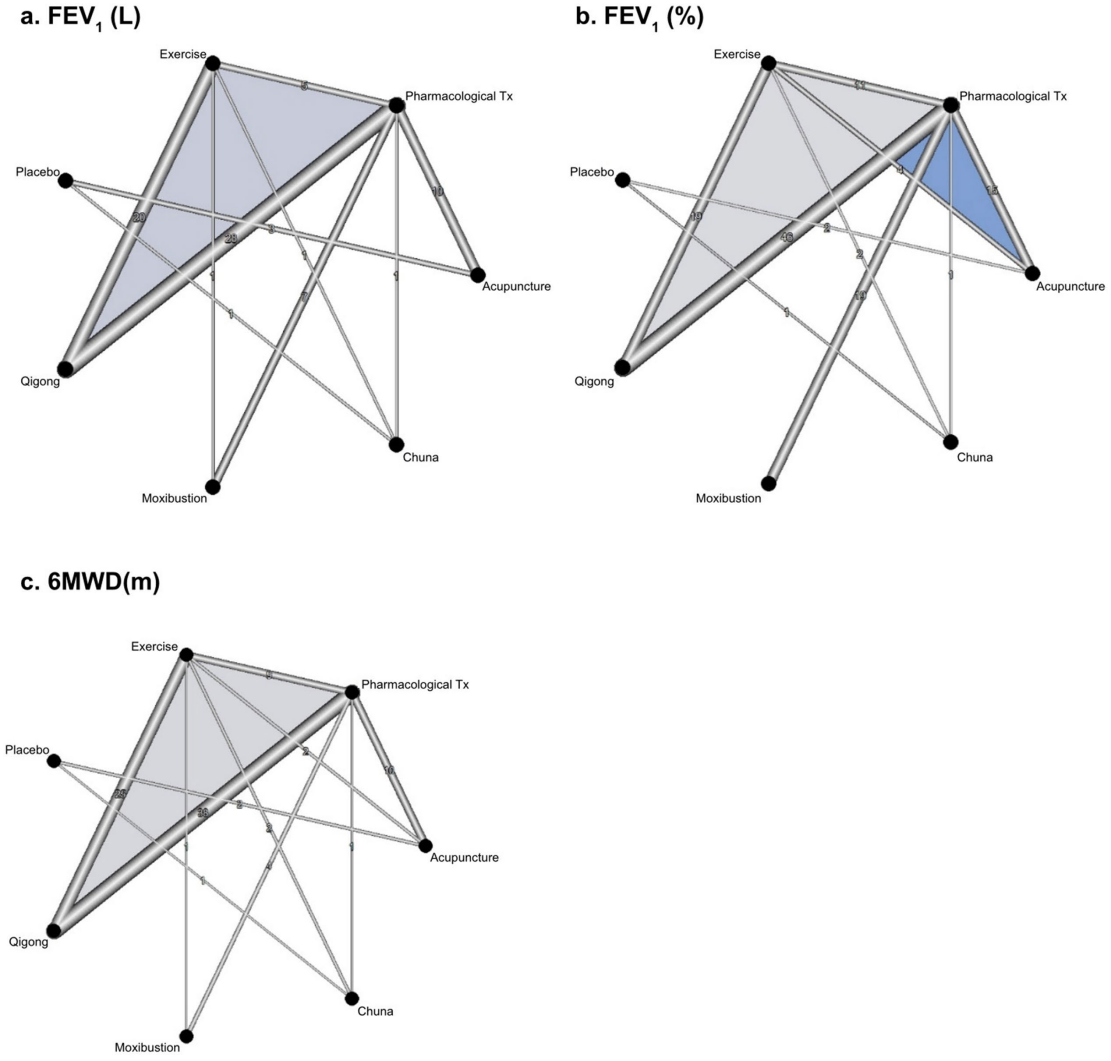
Network evidence map of eligible comparisons: **(A)** FEV_1_(L); **(B)** FEV_1_(%); **(C)** 6MWD(m). FEV_1_: forced expiratory volume in a 1 s; 6MWD, 6-min walking distance; Tx, treatment.

PMA of 133 RCTs (24–32, 34–46, 48–63, 65–69, 72–75, 77–104, 106–132, 134–141, 143–165) revealed that qigong, moxibustion, and acupuncture adjunctive therapies improved pulmonary function and exercise capacity, whereas chuna adjunctive therapy only improved exercise capacity (Supplement 4). In NMA, there were no issues with network connectivity and inconsistency in each of the three outcome measures (Figures 2A–C and Supplementary Figures S1–S3). NMA of the same 133 RCTs included in the PMA (24–32, 34–46, 48–63, 65–69, 72–75, 77–104, 106–132, 134–141, 143–165) showed that all types of EATM-NPI added to standard pharmacological treatment led to improvements in exercise capacity compared to standard pharmacological treatment alone, and qigong and moxibustion adjunctive therapies led to improvements in pulmonary functions.

**Figure 3:**
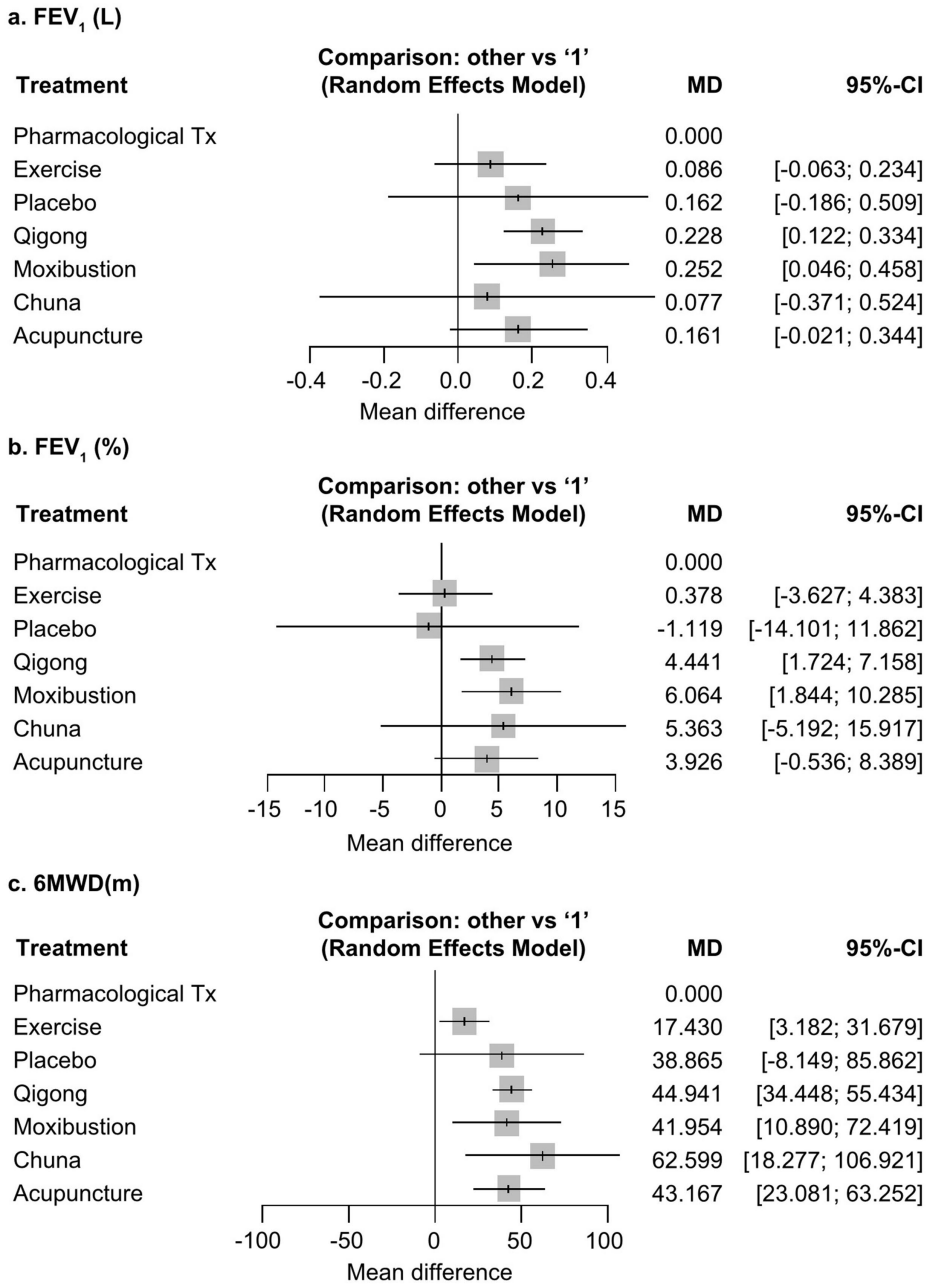
Network forest plots. All non-pharmacological interventions of East Asian Traditional Medicine (EATM-NPIs) were compared with pharmacological Tx (“1”) using a random-effects model: **(A)** FEV_1_(L); **(B)** FEV_1_(%); **(C)** 6MWD(m). FEV_1_: forced expiratory volume in a 1 s; 6MWD, 6-min walking distance; Tx, treatment; MD, mean difference; CI, confidence interval.

NMA results were as follows. FEV_1_ (L) was most improved by moxibustion (113, 114, 121–125, 128, 129), followed by qigong (25, 30–32, 35, 37, 39, 41, 43, 49, 50, 53, 56, 59, 60, 62, 63, 66–69, 72, 74, 77–82, 84, 85, 88, 91, 92, 94–96, 101–103, 107, 108, 110), acupuncture (137, 140, 141, 145, 148, 149, 152, 153, 156–159), placebo, chuna (132, 134, 136), exercise therapy, and standard pharmacological treatment (Table 2A). P-scores were as follows: moxibustion, B: 0.7752; qigong, A: 0.7537; acupuncture, D: 0.5603; placebo, 3: 0.5510; chuna, C: 0.3894; exercise therapy, 2: 0.3490; and standard pharmacological treatment 1: 0.1213. Compared to the reference (standard pharmacological treatment), chuna and acupuncture adjunctive therapies had overlapping CIs, and qigong (A: MD 0.228, 95% CI 0.122–0.334) and moxibustion adjunctive therapies (B: MD 0.252, 95% CI 0.046–0.458) were statistically significant (Figure 3A and Supplementary Table S3).

**Table 2:** Ranking test results.

(a) FEV_1_(L) P-score		(b) FEV_1_(%) P-score		(c) 6MWD(m) P-score	
B	0.7752	B	0.8094	C	0.8539
A	0.7537	C	0.6825	A	0.6599
D	0.5603	A	0.6753	D	0.6229
3	0.551	D	0.6181	B	0.5954
C	0.3894	3	0.2677	3	0.5408
2	0.349	2	0.2472	2	0.2157
1	0.1213	1	0.1998	1	0.0113

1, pharmacological treatment; 2, exercise; 3, placebo group; 6MWD, 6-min walking distance; A, Qigong; B, moxibustion; C, chuna; D, acupuncture; FEV_1_: forced expiratory volume in a 1 s.

FEV_1_ (%) was most improved by moxibustion (111–113, 115–122, 124–130), followed by chuna (132, 134–136), qigong (25–31, 34, 35, 37–41, 43–45, 49, 51, 53–55, 57, 59–63, 65, 66, 68, 69, 73, 75, 78, 80, 82, 84, 85, 88, 91, 93–99, 101–107, 110), acupuncture (137–141, 143–145, 151, 154–163, 165), placebo, exercise therapy, and standard pharmacological treatment (Table 2B). P-scores were as follows: moxibustion, B: 0.8094; chuna, C: 0.6825; qigong, A: 0.6753; acupuncture, D: 0.6181; placebo, 3: 0.2677; exercise therapy, 2: 0.2472; and standard pharmacological treatment, 1: 0.1998. Compared to the reference, chuna and acupuncture adjunctive therapies had overlapping CIs, and moxibustion (B: MD 6.064, 95% CI 1.844–10.285) and qigong adjunctive therapies (A: MD 4.441, 95% CI 1.724–7.158) were statistically significant (Figure 3B and Supplementary Table S3).

6MWD (m) was most improved by chuna (131, 134–136), followed by qigong (24–27, 29–31, 34, 36, 38, 40, 42, 43, 46, 48, 49, 52, 55, 58–63, 65–69, 72–74, 78, 80–84, 86–90, 94–100, 103, 107–109), acupuncture (137, 138, 141, 144, 146–148, 150, 151, 156, 160, 161, 164, 165), moxibustion (112, 117, 122, 123, 125), placebo, exercise therapy, and standard pharmacological treatment (Table 2C). P-scores were as follows: chuna, C: 0.8539; qigong, A: 0.6599; acupuncture, D: 0.6229; moxibustion, B: 0.5954; placebo, 3: 0.5408; exercise therapy, 2: 0.2157; and standard pharmacological treatment, 1: 0.0113. Compared to the reference, qigong adjunctive therapy (A: MD 44.941, 95% CI 34.448–55.434), moxibustion adjunctive therapy (B: MD 41.654, 95% CI 10.890–72.419), chuna adjunctive therapy (C: MD 62.599, 95% CI 18.277–106.921), and acupuncture adjunctive therapy (D: MD 43.167, 95% CI 23.081–63.252) were statistically significant (Figure 3C and Supplementary Table S3).

Regarding publication bias, the distribution was symmetrical overall, but studies with a large sample size had a greater impact on the results. Further, the Egger’s test result suggested a possibility of publication bias in FEV_1_; thus, caution is needed when interpreting the findings (*p* < 0.01) (Supplementary Figure S4).

## 4 Discussion

### 4.1 Summary of evidence

The present NMA sheds light on the comparative effects of EATM-NPI adjunctive therapy on pulmonary function and exercise capacity in patients with stable COPD. Compared to standard pharmacological treatment alone, qigong and moxibustion add-on therapies were more effective in improving FEV_1_, whereas qigong, moxibustion, chuna, and acupuncture add-on therapies were more effective in improving 6MWD. Hence, moxibustion may be considered over other adjunctive therapies when attempting to improve pulmonary function, whereas chuna may be considered first when the goal is to improve exercise capacity. AEs reported across the 27 studies (27, 58, 64, 66–71, 86, 93, 132, 133, 137, 140, 141, 147, 150–156, 159, 163, 164) were mostly mild. Some studies showed methodological issues related to random sequence generation, allocation concealment, and blinding of personnel.

### 4.2 Agreements and disagreements with other reviews

Dyspnea and decline in physical activities among patients with COPD are linked to secondary musculoskeletal problems (166). Therefore, it is possible that controlling these musculoskeletal problems could improve respiratory symptoms even without directly improving pulmonary function. Additionally, exacerbation of pulmonary functions (e.g., ventilatory limitation, gas exchange limitation, and cardiac limitation) can limit exercise capacity, and if musculoskeletal limitations, particularly limitation due to lower limb muscle dysfunction becomes chronic (167, 168), respiratory functions may weaken due to muscle dysfunction (169).

There is extensive research on the treatment of musculoskeletal disorders with the following modalities: chuna that is performed using hands, (12, 170, 171), qigong that trains the mind for meditation and places emphasis on smooth respiration and motions (172–174), acupuncture that stimulates soft tissues at acupoints using acupuncture needle (175, 176), and moxibustion that uses mugwort instead of needles (177). Our study is significant in that it sheds light on the potential of treating respiratory disorders by improving reversible and modifiable musculoskeletal issues.

Moxibustion has positive effects on respiratory diseases, such as lung cancer (178), pulmonary fibrosis (179), asthma (180), allergic rhinitis (181), and joint and spinal disorders (182, 183) through the regulation of inflammatory responses. Moxibustion regulates the body’s immune system (184) by improving the CD4+/CD8+ ratio, tumor necrosis factor-*α* levels, interleukin (IL)-10 levels, and IL-10/IL-6 ratio (185–187), thus effectively reducing inflammation (188). Our finding that moxibustion more effectively improved FEV_1_ than 6MWD did might have been attributable its use in treating chronic inflammation of the lungs.

Acupuncture may be effective in patients with COPD, as it reduces the chronic inflammatory state and concurrently improves chest wall stiffness. Acupuncture provides anti-inflammatory effects by regulating vascular responses and cytokines (142, 189–191) and clinically reduces inflammatory conditions, e.g., asthma (192), allergic rhinitis (193), and inflammatory bowel disease (194). Additionally, acupuncture mechanically stimulates connective tissues (195–198) and improves musculoskeletal flexibility (199), exhibiting effectiveness in various musculoskeletal disorders, e.g., low back pain (200, 201), neck pain (202), and frozen shoulder (203). However, in our NMA, acupuncture was effective in improving only 6MWD. Improving the mobility of the thorax and back may be a more beneficial pathway for patients with COPD, and further research on this is required.

Qigong involves following a step-by-step movement while regulating breathing, which promotes a sense of self-confidence, enhances flexibility and overall body balance, and activates bodily functions (59, 173, 174, 204–206). Although the exact mechanism underlying Qigong’s effects remains unclear, it has been reported to improve the 6MWD in patients with ischemic heart failure (207) and chronic heart failure (208), enhance FEV1 (%) in patients with non-small cell lung cancer (209), and manage pain in patients with chronic pain (210, 211). Qigong has demonstrated benefits in regulating the movement of the respiratory muscles and the overall musculoskeletal system. Meta-analyses conducted in 2014 (212) and 2019 (213) on the use of qigong in COPD reported statistically significant improvements in 6MWD, FEV1 (L), and FEV1 (%). Consistent with these previous meta-analyses, our study confirmed that qigong improves both FEV1 and 6MWD.

Chuna therapy may help improve exercise capacity in patients with COPD by alleviating chest stiffness through manual manipulation. Although the exact mechanism remains unclear, previous studies (10, 214) have suggested that Chuna therapy can improve both pulmonary function and exercise capacity. However, in this study’s NMA, while Chuna therapy demonstrated the most prominent improvement in 6MWD, significant effects were observed only in exercise capacity. Given the limited number of studies on Chuna therapy, large-scale follow-up studies are warranted.

### 4.3 Strengths and limitations of review

This is the first study to conduct the latest meta-analysis to compare the effectiveness of various EATM-NPIs in patients with COPD. We provided decisive evidence for the clinical prescription of EATM-NPI through NMA, and in addition to prior research, we also showed that EATM-NPI is a promising treatment approach for COPD.

A previous NMA on qigong (10) used FEV_1_/FVC (%) as the index for pulmonary function, but we used FEV_1_ instead. For COPD patients, the impairment of FEV_1_ is distinct and more crucial compared to FVC. Moreover, the GOLD guidelines categorize the severity of COPD based on FEV_1_, emphasizing the significance of FEV_1_ variability in COPD. In this study, we employed raw values of FEV_1_ rather than the FEV_1_/FVC ratio, distinguishing between absolute FEV_1_ in liters and the FEV_1_%. We conducted separate meta-analyses to examine the respective effects of each. There was no closed loop in the network diagram of Li et al.’s study, but we used a closed loop for a mixed comparison (10). Li et al. reported that Wuqinxi is the most effective traditional exercise modality for improving FEV_1_/FVC (%) and 6MWD (10). Herein, we examined broad categories of traditional interventions and showed that qigong is significantly more effective than other EATM-NPIs in improving FEV_1_ and 6MWD. Previous studies on acupuncture as an adjunctive therapy (11, 12) conducted PMA for acupuncture, Acu-TENS, moxibustion, acupressure, auricular acupuncture, and cupping, but we included acupuncture, Acu-TENS, warm needle acupuncture, auricular acupressure, electroacupuncture, pressing needle acupuncture, and acupressure in the acupuncture group and conducted NMA to compare it with other EATM-NPIs. One benefit of this analysis is that it provides the therapeutic efficacy estimates of each EATM-NPI as add-on treatment options to clinicians to improve motor and respiratory functions in patients with COPD who are refractory to standard treatment.

This study has several limitations. The scope of the included studies was broad, so it was not possible to control for all sources of heterogeneity. To conduct macroscopic analysis, we had to include heterogeneous treatments in each EATM-NPI, and the treatment schedules and severity of COPD varied across individual studies. Future studies should narrow the research question to focus on specific severities of COPD or capacities of intervention. Furthermore, our study was affected by the essential limitation of research on EATM-NPI; because of the intrinsic nature of non-pharmacological therapies, therapists could not be blinded, which led to low overall quality of the included studies and a lack of robustness in the NMA results. Moreover, we conducted a mixed comparison in macroscopic analysis, but the number of studies on each type of EATM-NPI varied, and few studies directly compared different EATM-NPIs. Finally, this study was limited by the lack of an evaluation of GRADE. Consequently, a constraint affects clearly determining the recommendation level. However, when RCTs conducted in China were assessed using GRADE, the recommendation levels were generally low, with little variation among different interventions. Considering these circumstances, the primary significance of this meta-analysis lies in its identification of information that can assist Traditional Chinese Medicine practitioners in clinical decision-making when faced with the challenge of selecting interventions under conditions of low research quality and evidence levels, i.e., in situations where information is limited. However, our evaluation of the evidence level for individual interventions was incomplete. Therefore, future research should involve subsequent studies applying the GRADE methodology to individual interventions through PMA rather than NMA.

### 4.4 Clinical and research implications

For patients with COPD, pharmacological treatment alone has limitations in terms of adverse drug reactions, compliance, and satisfaction. Non-pharmacological treatments for patients with COPD have been established since the mid-2000s, and evidence has been accumulating for treatment modalities such as qigong, moxibustion, chuna, and acupuncture. This study provides clinical practitioners with additional therapeutic options for managing stable COPD that does not respond to standard therapy. Particularly, various types of exercises are recommended for patients who still have diminished exercise capacity even with improved parameters on respiratory function tests. Subsequent studies should investigate various exercise therapies and indications for qigong, an EATM intervention, to establish evidence-based personalized treatment for patient symptoms. Future studies should also comply with Standards for Reporting Interventions in Clinical Trials of Acupuncture (215) and Consolidated Standards of Reporting Trials extension for non-pharmacologic treatment (216) to improve methodological quality. Moreover, the severity of COPD is an important consideration in the treatment selection and subsequent studies should further analyze this.

We did not investigate pharmacological treatments, such as herbal medicine, pharmacopuncture, and aromatherapy. In the future, studies should also investigate EATM-pharmacological interventions and conduct NMA of the synergistic effects with herbal medicine. Since head-to-head trials are difficult for EATM interventions because of a lack of resources, the findings of such studies would help present the optimal EATM intervention for each symptom and present implications for clinical practice and clinical practice guidelines. The search in this study was conducted until May 2022, so caution should be exercised when interpreting its results. If further research is conducted, a GRADE assessment should be incorporated for a more comprehensive evaluation.

## 5 Conclusion

Our study confirmed that the addition of EATM-NPI to standard therapy can aid symptom management in patients with COPD. We confirmed that moxibustion or qigong therapies as adjunctive therapies to standard treatment improve pulmonary function and exercise capacity, whereas chuna and acupuncture adjunctive therapies improve exercise capacity in patients with stable COPD. Clinically, we provide evidence supporting the use of moxibustion add-on therapy to improve FEV_1_ and chuna add-on therapy to improve 6MWD in patients who do not respond well to standard treatment. We look forward to future research efforts aimed at identifying responders to each NPI among patients with COPD, thereby optimizing NPI methods for this population.

## Data Availability

The raw data supporting the conclusions of this article will be made available by the authors, without undue reservation.
