# Supplementary material for: Comparative efficacy of traditional non-pharmacological add-on treatments in patients with stable chronic obstructive pulmonary disease: a systematic review and network meta-analysis
Source: Front Public Health. 2025 Feb 21;13:1410342. doi: 10.3389/fpubh.2025.1410342 (PMC11885152; doi:10.3389/fpubh.2025.1410342)

Supplementary Material

**Supplement 1. PRISMA NMA checklist**

**Supplement 2. Search strategies**

**Supplement 3. Tables**

**Table S1.** Summary of the primary outcomes

**Table S2.** Summary of risk of bias based on the Cochrane risk of bias tool

**Table S3.** League table

**Supplement 4. PMA results**

**Supplement 5. Figures**

**Figure S1.** Network summary and summary of inconsistency test results (global approach)

**Figure S2.** Summary of inconsistency test results (local approach)

**Figure S3.** Plot of inconsistency test results (local approach)

**Figure S4.** Funnel plot with Egger test values

**Supplement 1. PRISMA NMA checklist**

**PRISMA NMA Checklist of Items to Include When Reporting A Systematic Review Involving a Network Meta-analysis**

| **Section/Topic** | **Item #** | **Checklist Item** | **Reported on Page #** |
| --- | --- | --- | --- |
| **TITLE** |  |  |  |
| Title | 1 | Identify the report as a systematic review *incorporating a network meta-analysis (or related form of meta-analysis).* | Title, 1 |
|  |  |  |  |
| **ABSTRACT** |  |  |  |
| Structured summary | 2 | Provide a structured summary including, as applicable:  **Background:** main objectives  **Methods:** data sources; study eligibility criteria, participants, and interventions; study appraisal; and *synthesis methods, such as network meta-analysis.*  **Results:** number of studies and participants identified; summary estimates with corresponding confidence/credible intervals; *treatment rankings may also be discussed. Authors may choose to summarize pairwise comparisons against a chosen treatment included in their analyses for brevity.*  **Discussion/Conclusions:** limitations; conclusions and implications of findings.  **Other:** primary source of funding; systematic review registration number with registry name. | 1 |
|  |  |  |  |
| **INTRODUCTION** |  |  |  |
| Rationale | 3 | Describe the rationale for the review in the context of what is already known*, including mention of why a network meta-analysis has been conducted.* | 1-2 |
| Objectives | 4 | Provide an explicit statement of questions being addressed, with reference to participants, interventions, comparisons, outcomes, and study design (PICOS). | 2 |
|  |  |  |  |
| **METHODS** |  |  |  |
| Protocol and registration | 5 | Indicate whether a review protocol exists and if and where it can be accessed (e.g., Web address); and, if available, provide registration information, including registration number. | 2 |
| Eligibility criteria | 6 | Specify study characteristics (e.g., PICOS, length of follow-up) and report characteristics (e.g., years considered, language, publication status) used as criteria for eligibility, giving rationale. *Clearly describe eligible treatments included in the treatment network, and note whether any have been clustered or merged into the same node (with justification).* | 2-3 |
| Information sources | 7 | Describe all information sources (e.g., databases with dates of coverage, contact with study authors to identify additional studies) in the search and date last searched. | 3 |
| Search | 8 | Present full electronic search strategy for at least one database, including any limits used, such that it could be repeated. | Supplement 2 |
| Study selection | 9 | State the process for selecting studies (i.e., screening, eligibility, included in systematic review, and, if applicable, included in the meta-analysis). | 3 |
| Data collection process | 10 | Describe method of data extraction from reports (e.g., piloted forms, independently, in duplicate) and any processes for obtaining and confirming data from investigators. | 3 |
| Data items | 11 | List and define all variables for which data were sought (e.g., PICOS, funding sources) and any assumptions and simplifications made. | 3 |
| **Geometry of the network** | **S1** | Describe methods used to explore the geometry of the treatment network under study and potential biases related to it. This should include how the evidence base has been graphically summarized for presentation, and what characteristics were compiled and used to describe the evidence base to readers. | 3 |
| Risk of bias within individual studies | 12 | Describe methods used for assessing risk of bias of individual studies (including specification of whether this was done at the study or outcome level), and how this information is to be used in any data synthesis. | 3 |
| Summary measures | 13 | State the principal summary measures (e.g., risk ratio, difference in means). *Also describe the use of additional summary measures assessed, such as treatment rankings and surface under the cumulative ranking curve (SUCRA) values, as well as modified approaches used to present summary findings from meta-analyses.* | 3 |
| Planned methods of analysis | 14 | Describe the methods of handling data and combining results of studies for each network meta-analysis. This should include, but not be limited to:   - *Handling of multi-arm trials;* - *Selection of variance structure;* - *Selection of prior distributions in Bayesian analyses; and* - *Assessment of model fit.* | 3 |
| **Assessment of Inconsistency** | **S2** | Describe the statistical methods used to evaluate the agreement of direct and indirect evidence in the treatment network(s) studied. Describe efforts taken to address its presence when found. | 3 |
| Risk of bias across studies | 15 | Specify any assessment of risk of bias that may affect the cumulative evidence (e.g., publication bias, selective reporting within studies). | 3 |
| Additional analyses | 16 | Describe methods of additional analyses if done, indicating which were pre-specified. This may include, but not be limited to, the following:   - Sensitivity or subgroup analyses; - Meta-regression analyses; - *Alternative formulations of the treatment network; and* - *Use of alternative prior distributions for Bayesian analyses (if applicable).* | None |
|  |  |  |  |
| **RESULTS†** |  |  |  |
| Study selection | 17 | Give numbers of studies screened, assessed for eligibility, and included in the review, with reasons for exclusions at each stage, ideally with a flow diagram. | Figure 1. |
| **Presentation of network structure** | **S3** | Provide a network graph of the included studies to enable visualization of the geometry of the treatment network. | Figure 2. |
| **Summary of network geometry** | **S4** | Provide a brief overview of characteristics of the treatment network. This may include commentary on the abundance of trials and randomized patients for the different interventions and pairwise comparisons in the network, gaps of evidence in the treatment network, and potential biases reflected by the network structure. | 5 |
| Study characteristics | 18 | For each study, present characteristics for which data were extracted (e.g., study size, PICOS, follow-up period) and provide the citations. | Table 1. |
| Risk of bias within studies | 19 | Present data on risk of bias of each study and, if available, any outcome level assessment. | Table S2. |
| Results of individual studies | 20 | For all outcomes considered (benefits or harms), present, for each study: 1) simple summary data for each intervention group, and 2) effect estimates and confidence intervals. *Modified approaches may be needed to deal with information from larger networks.* | Table S1. |
| Synthesis of results | 21 | Present results of each meta-analysis done, including confidence/credible intervals. *In larger networks, authors may focus on comparisons versus a particular comparator (e.g. placebo or standard care), with full findings presented in an appendix. League tables and forest plots may be considered to summarize pairwise comparisons.* If additional summary measures were explored (such as treatment rankings), these should also be presented. | Figure 3, Table 2 and Table S3. |
| **Exploration for inconsistency** | **S5** | Describe results from investigations of inconsistency. This may include such information as measures of model fit to compare consistency and inconsistency models, *P* values from statistical tests, or summary of inconsistency estimates from different parts of the treatment network. | Figure S1-3. |
| Risk of bias across studies | 22 | Present results of any assessment of risk of bias across studies for the evidence base being studied. | Figure S4. |
| Results of additional analyses | 23 | Give results of additional analyses, if done (e.g., sensitivity or subgroup analyses, meta-regression analyses*, alternative network geometries studied, alternative choice of prior distributions for Bayesian analyses,* and so forth). | None |
|  |  |  |  |
| **DISCUSSION** |  |  |  |
| Summary of evidence | 24 | Summarize the main findings, including the strength of evidence for each main outcome; consider their relevance to key groups (e.g., healthcare providers, users, and policy-makers). | 5 |
| Limitations | 25 | Discuss limitations at study and outcome level (e.g., risk of bias), and at review level (e.g., incomplete retrieval of identified research, reporting bias). *Comment on the validity of the assumptions, such as transitivity and consistency. Comment on any concerns regarding network geometry (e.g., avoidance of certain comparisons).* | 24 |
| Conclusions | 26 | Provide a general interpretation of the results in the context of other evidence, and implications for future research. | 26 |
|  |  |  |  |
| **FUNDING** |  |  |  |
| Funding | 27 | Describe sources of funding for the systematic review and other support (e.g., supply of data); role of funders for the systematic review. This should also include information regarding whether funding has been received from manufacturers of treatments in the network and/or whether some of the authors are content experts with professional conflicts of interest that could affect use of treatments in the network. | 26 |

PICOS = population, intervention, comparators, outcomes, study design.

* Text in italics indicateS wording specific to reporting of network meta-analyses that has been added to guidance from the PRISMA statement.

† Authors may wish to plan for use of appendices to present all relevant information in full detail for items in this section.

**Supplement 2. Search strategies**

| **The Cochrane Library** | |
| --- | --- |
| #1 | MeSH DESCRIPTOR "Pulmonary Disease, Chronic Obstructive" Explode All Trees |
| #2 | MeSH DESCRIPTOR "Bronchitis, Chronic" Explode All Trees |
| #3 | MeSH DESCRIPTOR "Pulmonary Emphysema" Explode All Trees |
| #4 | (obstruct*) near/3 (pulmonary or lung* or airway* or airflow* or bronch* or respirat*):ti,ab,kw |
| #5 | (COPD or COAD or COBD):ti,ab,kw |
| #6 |  |
| #7 | MeSH descriptor: [acupuncture] explode all trees |
| #8 | MeSH descriptor: [acupuncture therapy] explode all trees |
| #9 | MeSH descriptor: [acupuncture, ear] explode all trees |
| #10 | MeSH descriptor: [acupuncture points] explode all trees |
| #11 | MeSH descriptor: [acupressure] explode all trees |
| #12 | MeSH descriptor: [meridians] explode all trees |
| #13 | MeSH descriptor: [trigger points] explode all trees |
| #14 | MeSH descriptor: [dry needling] explode all trees |
| #15 | (acup*) |
| #16 | (acupuncture):ti,ab,kw |
| #17 | (acupuncture therap*):ti,ab,kw |
| #18 | (auricular acup*):ti,ab,kw |
| #19 | (auricular needl*):ti,ab,kw |
| #20 | (ear acup*):ti,ab,kw |
| #21 | (acupoint*):ti,ab,kw |
| #22 | (acupuncture point*):ti,ab,w |
| #23 | (acupressure):ti,ab,kw |
| #24 | (meridian*):ti,ab,kw |
| #25 | (manual acup*):ti,ab,kw |
| #26 | (warm acup*):ti,ab,kw |
| #27 | (laser acup*):ti,ab,kw |
| #28 | MeSH descriptor: [electroacupuncture] explode all trees |
| #29 | MeSH descriptor: [transcutaneous electric nerve stimulation] explode all trees |
| #30 | (electroacup*):ti,ab,kw |
| #31 | (electric acup*):ti,ab,kw |
| #32 | (electrical acup*):ti,ab,kw |
| #33 | (transcutaneous electric nerve stimulation):ti,ab,kw |
| #34 | (electro stimulat*):ti,ab,kw |
| #35 | (TENS):ti,ab,kw |
| #36 | (electr*):ti,ab,kw near/2 ((acup*):ti,ab,kw OR (needl*):ti,ab,kw OR (stimulat*):ti,ab,kw) |
| #37 | MeSH descriptor: [moxibustion] explode all trees |
| #38 | (moxibustion):ti,ab,kw |
| #39 | (moxa):ti,ab,kw |
| #40 | (mugwort):ti,ab,kw |
| #41 | MeSH descriptor: [cupping therapy] explode all trees |
| #42 | MeSH descriptor: [bloodletting] explode all trees |
| #43 | (cupping*):ti,ab,kw |
| #44 | ((dry):ti,ab,kw OR (wet):ti,ab,kw OR (flash):ti,ab,kw OR (herbal):ti,ab,kw OR (moving):ti,ab,kw OR (vacuum):ti,ab,kw OR (fire):ti,ab,kw OR (suction):ti,ab,kw) NEAR/2 (cup*):ti,ab,kw |
| #45 | (ventouse):ti,ab,kw |
| #46 | (blood$letting):ti,ab,kw |
| #47 | (hijama):ti,ab,kw |
| #48 | MeSH descriptor: [Chiropractic] explode all trees |
| #49 | MeSH descriptor: [Manipulation, Spinal] explode all trees |
| #50 | MeSH descriptor: [Manipulation, Chiropractic] explode all trees |
| #51 | MeSH descriptor: [Manipulation, Osteopathic] explode all trees |
| #52 | MeSH descriptor: [Therapy, Soft Tissue] explode all trees |
| #53 | (manual NEAR therap*):ti,ab,kw |
| #54 | (mobili?ation):ti,ab,kw |
| #55 | (chiroprac*):ti,ab,kw |
| #56 | (osteopath*):ti,ab,kw |
| #57 | (spin* NEAR/2 manipulat*):ti,ab,kw |
| #58 | (muscle NEXT energy NEXT technique*):ti,ab,kw |
| #59 | (myofascial release*):ti,ab,kw |
| #60 | (ischemic compression):ti,ab,kw |
| #61 | ((strain OR counterstrain) NEAR technique*):ti,ab,kw |
| #62 | (tuina):ti,ab,kw |
| #63 | (chuna):ti,ab,kw |
| #64 | MeSH descriptor: [qigong] explode all trees |
| #65 | MeSH descriptor: [tai ji] explode all trees |
| #66 | (tai chi):ti,ab,kw |
| #67 | ("tai ji"):ti,ab,kw |
| #68 | (taichichuan):ti,ab,kw |
| #69 | (qigoing):ti,ab,kw |
| #70 | (chi kung):ti,ab,kw |
| #71 | (wuqinxi):ti,ab,kw |
| #72 | (five animal):ti,ab,kw |
| #73 | (liuzijue):ti,ab,kw |
| #74 | ("six character"):ti,ab,kw |
| #75 | ("six syllable"):ti,ab,kw |
| #76 | ("six letter*"):ti,ab,kw |
| #77 | (baduanjin):ti,ab,kw |
| #78 |  |
| #79 | **#6 and #78** |
| #79 | **#6 and #78 in Trials(All Years)** |

| **Medline via PubMed** | |
| --- | --- |
| 1 | "pulmonary disease, chronic obstructive"[MeSH Terms] |
| 2 | "bronchitis, chronic"[MeSH Terms] |
| 3 | "pulmonary emphysema"[MeSH Terms] |
| 4 | emphysema*[Title/Abstract] |
| 5 | chronic bronchiti*[Title/Abstract] |
| 6 | (obstruct*[Title/Abstract]) AND pulmonary[Title/Abstract] |
| 7 | (obstruct*[Title/Abstract]) AND lung[Title/Abstract] |
| 8 | (obstruct*[Title/Abstract]) AND airway[Title/Abstract] |
| 9 | (obstruct*[Title/Abstract]) AND airflow[Title/Abstract] |
| 10 | (obstruct*[Title/Abstract]) AND bronchial[Title/Abstract] |
| 11 | (obstruct*[Title/Abstract]) AND **respirat***[Title/Abstract] |
| 12 | COPD[Title/Abstract] |
| 13 | COAD[Title/Abstract] |
| 14 | COBD[Title/Abstract] |
| **15** | **or/1-14** |
| 16 | acupuncture[MeSH Terms] |
| 17 | acupuncture, ear[MeSH Terms] |
| 18 | acupuncture points[MeSH Terms] |
| 19 | acupressure[MeSH Terms] |
| 20 | meridians[MeSH Terms] |
| 21 | trigger points[MeSH Terms] |
| 22 | dry needling[MeSH Terms] |
| 23 | acup*[Text Word] |
| 24 | acupuncture[Title/Abstract] |
| 25 | acupuncture therap*[Title/Abstract] |
| 26 | auricular acup*[Title/Abstract] |
| 27 | auricular needl*[Title/Abstract] |
| 28 | ear acup*[Title/Abstract] |
| 29 | acupoint*[Title/Abstract] |
| 30 | acupuncture point*[Title/Abstract] |
| 31 | acupressure[Title/Abstract] |
| 32 | meridian*[Title/Abstract] |
| 33 | manual acup*[Title/Abstract] |
| 34 | warm acup*[Title/Abstract] |
| 35 | laser acup*[Title/Abstract] |
| 36 | electroacupuncture[MeSH Terms] |
| 37 | transcutaneous electric nerve stimulation[MeSH Terms] |
| 38 | electroacup*[Title/Abstract] |
| 39 | electro acup*[Title/Abstract] |
| 40 | electric acup*[Title/Abstract] |
| 41 | electrical acup*[Title/Abstract] |
| 42 | transcutaneous electric nerve stimulation[Title/Abstract] |
| 43 | "electro stimulat*"[Title/Abstract] |
| 44 | TENS[Title/Abstract] |
| 45 | electric*[Title/Abstract] AND ("acup*"[Title/Abstract] OR "needl*"[Title/Abstract] OR "stimulat*"[Title/Abstract]) |
| 46 | moxibustion[MeSH Terms] |
| 47 | moxibustion[Title/Abstract] |
| 48 | moxa[Title/Abstract] |
| 49 | mugwort[Title/Abstract] |
| 50 | cupping therapy[MeSH Terms] |
| 51 | bloodletting[MeSH Terms] |
| 52 | cupping*[Title/Abstract] |
| 53 | dry cup*[Title/Abstract] |
| 54 | wet cup*[Title/Abstract] |
| 55 | ventouse[Title/Abstract] |
| 56 | bloodletting[Title/Abstract] |
| 57 | blood letting[Title/Abstract] |
| 58 | cupping therapy[Title/Abstract] |
| 59 | flash cup*[Title/Abstract] |
| 60 | herbal cup*[Title/Abstract] |
| 61 | moving cup*[Title/Abstract] |
| 62 | hijama[Title/Abstract] |
| 63 | bleeding cup*[Title/Abstract] |
| 64 | vacuum cup*[Title/Abstract] |
| 65 | fire cup*[Title/Abstract] |
| 66 | suction cup*[Title/Abstract] |
| 67 | chiropractic[MeSH Terms] |
| 68 | manipulation, spinal[MeSH Terms] |
| 69 | manipulation, chiropractic[MeSH Terms] |
| 70 | manipulation, osteopathic[MeSH Terms] |
| 71 | therapy, soft tissue[MeSH Terms] |
| 72 | manual therap*[Title/Abstract] |
| 73 | mobilization[Title/Abstract] |
| 74 | mobilisation[Title/Abstract] |
| 75 | chiroprac*[Title/Abstract] |
| 76 | osteopath*[Title/Abstract] |
| 77 | spinal manipulat*[Title/Abstract] |
| 78 | muscle energy technique*[Title/Abstract] |
| 79 | myofascial release*[Title/Abstract] |
| 80 | ischemic compression[Title/Abstract] |
| 81 | ((strain[Title/Abstract]) OR (counterstrain[Title/Abstract])) AND (technique*[Title/Abstract]) |
| 82 | tuina[Title/Abstract] |
| 83 | chuna[Title/Abstract] |
| 84 | qigong[MeSH Terms] |
| 85 | tai ji[MeSH Terms] |
| 86 | tai chi[Title/Abstract] |
| 87 | "tai ji"[Title/Abstract] |
| 88 | taichichuan[Title/Abstract] |
| 89 | qigong[Title/Abstract] |
| 90 | qi gong[Title/Abstract] |
| 91 | chi kung[Title/Abstract] |
| 92 | wuqinxi[Title/Abstract] |
| 93 | five animal[Title/Abstract] |
| 94 | liuzijue[Title/Abstract] |
| 95 | six character[Title/Abstract] |
| 96 | six syllable[Title/Abstract] |
| 97 | six letter*[Title/Abstract] |
| 98 | baduanjin[Title/Abstract] |
| **99** | **or/16-99** |
| **100** | **15 and 99** |
| 101 | ("randomized controlled trial"[Publication Type] OR "controlled clinical trial"[Publication Type] OR "randomized"[Title/Abstract] OR "placebo"[Title/Abstract] OR "clinical trials as topic"[MeSH Terms:noexp] OR "randomly"[Title/Abstract] OR "trial"[Title]) NOT ("animals"[MeSH Terms] NOT "humans"[MeSH Terms]) |
| **102** | **100 and 101** |

| **Elsevier via EMBASE** | |
| --- | --- |
| #1 | chronic obstructive lung disease'/exp |
| #2 | chronic bronchitis'/exp |
| #3 | lung emphysema'/exp |
| #4 | emphysema$:ti,ab,kw |
| #5 | (chronic$ NEXT/2 bronchiti*):ti,ab,kw |
| #6 | (obstruct* NEXT/3 (pulmonary OR lung$ OR airway$ OR airflow$ OR bronch* OR respirat*)):ti,ab,kw |
| #7 | copd:ti,ab,kw |
| #8 | coad:ti,ab,kw |
| #9 | cobd:ti,ab,kw |
| **#10** | **#1 OR #2 OR #3 OR #4 OR #5 OR #6 OR #7 OR #8 OR #9** |
| #11 | acupuncture'/exp |
| #12 | auricular acupuncture'/exp |
| #13 | acupuncture point'/exp |
| #14 | acupressure'/exp |
| #15 | trigger point'/exp |
| #16 | dry needling'/exp |
| #17 | acup* |
| #18 | acupuncture:ti,ab,kw |
| #19 | acupuncture therap*':ti,ab,kw |
| #20 | auricular acup*':ti,ab,kw |
| #21 | auricular needl*':ti,ab,kw |
| #22 | ear acup*':ti,ab,kw |
| #23 | acupoint*:ti,ab,kw |
| #24 | acupuncture point*':ti,ab,kw |
| #25 | acupressure:ti,ab,kw |
| #26 | meridian*:ti,ab,kw |
| #27 | manual acup*':ti,ab,kw |
| #28 | warm acup*':ti,ab,kw |
| #29 | laser acup*':ti,ab,kw |
| #30 | electroacupuncture'/exp |
| #31 | transcutaneous electrical nerve stimulation'/exp |
| #32 | electro&acup*:ti,ab,kw |
| #33 | electric acup*':ti,ab,kw |
| #34 | electrical acup*':ti,ab,kw |
| #35 | transcutaneous electric nerve stimulation':ti,ab,kw |
| #36 | electro stimulat*':ti,ab,kw |
| #37 | TENS:ti,ab,kw |
| #38 | (electr* NEAR/2 needl*):ti,ab,kw |
| #39 | (electr* NEAR/2 acupoint*):ti,ab,kw |
| #40 | (electr* NEAR/2 stimulat*):ti,ab,kw |
| #41 | moxibustion'/exp |
| #42 | moxibustion:ti,ab,kw |
| #43 | moxa:ti,ab,kw |
| #44 | mugwort:ti,ab,kw |
| #45 | cupping therapy'/exp |
| #46 | bloodletting'/exp |
| #47 | cupping*:ti,ab,kw |
| #48 | ((dry OR wet OR flash OR herbal OR moving OR vacuum OR fire OR suction) NEAR/2 cup*):ti,ab,kw |
| #49 | ventouse:ti,ab,kw |
| #50 | blood$letting:ti,ab,kw |
| #51 | hijama:ti,ab,kw |
| #52 | chiropractic manipulation'/exp |
| #53 | osteopathic manipulation'/exp |
| #54 | spine manipulation'/exp |
| #55 | soft tissue therapy'/exp |
| #56 | joint mobilization'/exp |
| #57 | (manual NEAR/1 therap*):ti,ab,kw |
| #58 | mobili?ation:ti,ab,kw |
| #59 | chiroprac*:ti,ab,kw |
| #60 | osteopath*:ti,ab,kw |
| #61 | (spin* NEAR/2 manipulat*):ti,ab,kw |
| #62 | muscle energy technique*':ti,ab,kw |
| #63 | myofascial release*':ti,ab,kw |
| #64 | ischemic compression':ti,ab,kw |
| #65 | ((strain OR counterstrain) NEAR/1 technique*):ti,ab,kw |
| #66 | tuina:ti,ab,kw |
| #67 | chuna:ti,ab,kw |
| #68 | qigong'/exp |
| #69 | tai chi'/exp |
| #70 | tai chi':ti,ab,kw |
| #71 | tai ji':ti,ab,kw |
| #72 | taichiquan:ti,ab,kw |
| #73 | qigong:ti,ab,kw |
| #74 | chi kung':ti,ab,kw |
| #75 | wuqinxi:ti,ab,kw |
| #76 | five animal':ti,ab,kw |
| #77 | liuzijue:ti,ab,kw |
| #78 | six character':ti,ab,kw |
| #79 | six syllable':ti,ab,kw |
| #80 | six letter*':ti,ab,kw |
| #81 | baduanjin:ti,ab,kw |
| **#82** | **#11 OR #12 OR #13 OR #14 OR #15 OR #16 OR #17 OR #18 OR #19 OR #20 OR #21 OR #22 OR #23 OR #24 OR #25 OR #26 OR #27 OR #28 OR #29 OR #30 OR #31 OR #32 OR #33 OR #34 OR #35 OR #36 OR #37 OR #38 OR #39 OR #40 OR #41 OR #42 OR #43 OR #44 OR #45 OR #46 OR #47 OR #48 OR #49 OR #50 OR #51 OR #52 OR #53 OR #54 OR #55 OR #56 OR #57 OR #58 OR #59 OR #60 OR #61 OR #62 OR #63 OR #64 OR #65 OR #66 OR #67 OR #68 OR #69 OR #70 OR #71 OR #72 OR #73 OR #74 OR #75 OR #76 OR #77 OR #78 OR #79 OR #80 OR #81** |
| **#83** | **#10 AND #82** |
| #84 | crossover procedure':de OR 'double-blind procedure':de OR 'randomized controlled trial':de OR 'single-blind procedure':de OR (random* OR factorial* OR crossover* OR cross NEXT/1 over* OR placebo* OR doubl* NEAR/1 blind* OR singl* NEAR/1 blind* OR assign* OR allocat* OR volunteer*):de,ab,ti |
| #85 | **random*:ab,ti OR placebo*:de,ab,ti OR (double NEXT/1 blind*):ab,ti** |
| **#86** | #83 AND #84 |
| **#87** | **#83 AND #85** |

| **CNKI** | |
| --- | --- |
| #1 | COPD |
| #2 | 慢性阻塞性肺疾病 |
| #3 | 慢性阻塞性肺病 |
| #4 | 慢性阻塞性肺 |
| #5 | 肺气肿 |
| #6 | 慢性支气管炎 |
| #7 | 慢性气管炎 |
| #8 | 慢阻肺疾病 |
| #9 | 慢阻肺病 |
| #10 | 慢阻肺 |
| **#11** | **or/#1-#10** |
| #12 | 针 |
| #13 | 针刺 |
| #14 | 耳针 |
| #15 | 温针 |
| #16 | 火针 |
| #17 | 經穴 |
| #18 | 穴位 |
| #19 | 电针 |
| #20 | 灸 |
| #21 | 针灸 |
| #25 | 灸法 |
| #26 | 艾灸 |
| #27 | 罐 |
| #28 | 拔罐 |
| #29 | 手法治疗 |
| #30 | 推拿 |
| #31 | 捏脊 |
| #32 | 正骨 |
| #33 | 整骨 |
| #34 | 氣功 |
| #35 | 太極 |
| #36 | 太極拳 |
| #37 | 五禽戱 |
| #38 | 六字訣 |
| #39 | 八段錦 |
| **#40** | **or/#12-#39** |
| **#41** | **#11 and #40** |
| #42 | 随机 |
| **#43** | **#41 and #42** |

**Supplement 3. Tables**

Table S1. Summary of the primary outcomes

| **Category** | | **Author (year)/Country, city** | **Treatment** | **FEV_1_ (L)** | | | **FEV_1_ (%, prd)** | | | **FVC (L)** | | | **FVC (%, prd)** | | | **FEV_1_/FVC (%)** | | | **6MWD (m)** | | |
| --- | --- | --- | --- | --- | --- | --- | --- | --- | --- | --- | --- | --- | --- | --- | --- | --- | --- | --- | --- | --- | --- |
|  |  |  |  | **baseline** | **final** |  | **baseline** | **final** |  | **baseline** | **final** |  | **baseline** | **final** |  | **baseline** | **final** |  | **baseline** | **final** |  |
|  |  |  |  | **mean ± sd** | **mean ± sd** | **MD ± sd** | **mean ± sd** | **mean ± sd** | **MD ± sd** | **mean ± sd** | **mean ± sd** | **MD ± sd** | **mean ± sd** | **mean ± sd** | **MD ± sd** | **mean ± sd** | **mean ± sd** | **MD ± sd** | **mean ± sd** | **mean ± sd** | **MD ± sd** |
| Qigong | Qigong (气功) - unspecified | Dong (2021)/China, Jinan | Qigong exercise |  |  |  |  |  |  |  |  |  |  |  |  |  |  |  | 530.00 ± 875.38 | 559.80 ± 55.8 |  |
|  |  |  | Cycle ergometer exercise |  |  |  |  |  |  |  |  |  |  |  |  |  |  |  | 520.60 ± 35.33 | 549.60 ± 26.94 |  |
|  | Health qigong integrated for lung health | Li (2019)/China, Shanghai | Health qigong integrated for lung health | 1.6 ± 0.6 | 1.7 ± 0.6 |  | 59.2 ± 4.3 | 59.6 ± 20.0 |  | 2.7 ± 0.7 | 2.8 ± 0.6 |  |  |  |  | 58.9 ± 10.4 | 58.5 ± 11.0 |  | 501.26 ± 74.08 | 535.78 ± 55.09 |  |
|  |  |  | Usual care | 1.5 ± 0.1 | 1.4 ± 0.1 |  | 59.2 ± 18.2 | 57.5 ± 16.9 |  | 2.5 ± 0.2 | 2.4 ± 0.2 |  |  |  |  | 60.0 ± 8.8 | 57.5 ± 13.6 |  | 440.74 ± 82.00 | 434.07 ± 83.12 |  |
|  |  | Liu (2012)/China, Shanghai | Health qigong integrated for lung health |  |  |  | 74.43 ± 12.93 | 75.47 ± 12.43 |  |  |  |  |  |  |  | 60.73 ± 6.18 | 61.93 ± 5.94 |  | 375.28 ± 31.12 | 434.53 ± 28.70 |  |
|  |  |  | Usual care |  |  |  | 75.31 ± 13.79 | 75.34 ± 12.89 |  |  |  |  |  |  |  | 61.43 ± 6.17 | 61.85 ± 5.90 |  | 381.91 ± 28.94 | 407.14 ± 22.75 |  |
|  |  |  | PR |  |  |  | 75.31 ± 12.84 | 76.22 ± 12.47 |  |  |  |  |  |  |  | 61.27 ± 5.86 | 62.02 ± 5.75 |  | 378.91 ± 31.03 | 435.69 ± 23.78 |  |
|  |  | Liu (2011)/China, Jiangsu | Health qigong integrated for lung health |  |  |  | 63.50 ± 8.03 | 65.35 ± 8.87 |  |  |  |  |  |  |  | 57.26 ± 5.97 | 59.33 ± 6.59 |  | 352.50 ± 19.92 | 430.19 ± 25.86 |  |
|  |  |  | Usual care |  |  |  | 63.67 ± 7.84 | 64.67 ± 8.16 |  |  |  |  |  |  |  | 57.64 ± 5.86 | 58.71 ± 6.35 |  | 361.89 ± 19.66 | 412.38 ± 17.48 |  |
|  |  |  | Pursed-lip breathing, walking exercise |  |  |  | 64.82 ± 7.46 | 66.24 ± 7.80 |  |  |  |  |  |  |  | 57.71 ± 5.52 | 58.94 ± 5.91 |  | 353.76 ± 13.51 | 427.82 ± 22.64 |  |
|  | Wuqinxi (五禽戏) | Wei (2015)/China, Bozhou | Wuquinxi |  |  |  | 64.91 ± 8.72 | 58.97±5.63 |  |  |  |  |  |  |  | 62.14 ± 5.40 | 58.97±5.63 |  |  |  |  |
|  |  |  | Usual care |  |  |  | 64.55 ± 6.03 | 58.82±5.76 |  |  |  |  |  |  |  | 61.26 ± 6.25 | 58.97±5.63 |  |  |  |  |
|  |  | Zhao (2015)/China, Zhengzhou | Wuquinxi |  |  |  | 55.68 ± 4.49 | 55.92 ± 3.95 |  |  |  |  |  |  |  | 64.34 ± 3.96 | 64.62 ± 3.65 |  | 398.96 ± 21.41 | 439.04 ± 31.27 |  |
|  |  |  | Usual care |  |  |  | 57.46 ± 4.31 | 57.77 ± 3.89 |  |  |  |  |  |  |  | 65.44 ± 3.36 | 65.17 ± 2.28 |  | 403.12 ± 20.97 | 401.59 ± 26.67 |  |
|  |  | Yao (2021)/China, Zhangjiajie | Wuquinxi | 1.13 ± 0.32 | 1.31 ± 0.30 |  | 57.74 ± 6.80 | 66.06 ± 9.85 |  |  |  |  |  |  |  | 50.61 ± 10.93 | 61.48 ± 17.54 |  | 407.86 ± 68.30 | 440.03 ± 73.89 |  |
|  |  |  | Usual care | 1.21 ± 0.31 | 1.25 ± 0.31 |  | 57.94 ± 10.64 | 58.94 ± 10.16 |  |  |  |  |  |  |  | 52.85 ± 13.39 | 54.03 ± 16.03 |  | 406.15 ± 74.58 | 420.64 ± 79.05 |  |
|  | Liuzijue (六字诀) | Jiang (2017)/China, Changsha | Liuzijue | 1.11 ± 0.25 | 1.72 ± 0.25 |  | 58.33 ± 7.07 | 70.43 ± 6.18 |  | 1.80 ± 0.49 | 2.20 ± 0.61 |  |  |  |  | 61.97 ± 4.69 | 78.18 ± 5.34 |  | 288.88 ± 29.44 | 364.98 ± 29.44 |  |
|  |  |  | Pursed-lip breathing | 1.16 ± 0.35 | 1.20 ± 0.35 |  | 59.96 ± 8.03 | 61.51 ± 5.45 |  | 1.84 ± 0.56 | 1.84 ± 0.56 |  |  |  |  | 62.71 ± 3.31 | 65.21 ± 4.32 |  | 277.62 ± 31.04 | 339.45 ± 31.04 |  |
|  |  |  | Enhanced Liuzijue | 1.21 ± 0.31 | 2.33 ± 0.31 |  | 59.05 ± 7.23 | 74.97 ± 7.54 |  | 1.96 ± 0.43 | 2.99 ± 0.52 |  |  |  |  | 62.03 ± 3.96 | 77.92 ± 3.89 |  | 280.82 ± 31.18 | 421.32 ± 31.18 |  |
|  |  | Hou (2017)/China, Xianyang | Simplified Liuzijue | 1.23 ± 0.42 | 1.75 ± 0.45 |  |  |  |  |  |  |  |  |  |  | 47.21 ± 7.21 | 80.20 ± 9.75 |  |  |  |  |
|  |  |  | Usual care | 1.21 ± 0.39 | 1.45 ± 0.32 |  |  |  |  |  |  |  |  |  |  | 43.70 ± 6.81 | 70.15 ± 7.14 |  |  |  |  |
|  |  | Zhang (2009)/China, Fujian | Liuzijue |  |  |  |  |  |  |  |  |  |  |  |  |  |  |  | 374.81 ± 25.50 |  | 23.05 ± 5.43 |
|  |  |  | Usual care |  |  |  |  |  |  |  |  |  |  |  |  |  |  |  | 375.71 ± 15.92 |  | 6.11 ± 3.31 |
|  |  | Wu (2018)/China, Shanghai (a) | Liuzijue on land |  |  |  | 55 ± 17 | 57 ± 16 |  |  |  |  |  |  |  | 60 ± 18 | 57 ± 13 |  |  |  |  |
|  |  |  | Usual care |  |  |  | 59 ± 17 | 57 ± 14 |  |  |  |  |  |  |  | 60 ± 7.57 | 57 ± 14 |  |  |  |  |
|  |  |  | Liuzijue in the water |  |  |  | 59 ± 22 | 60 ± 22 |  |  |  |  |  |  |  | 62 ± 14 | 60 ± 15 |  |  |  |  |
|  |  | Zhu (2011)/China, Nanjing | Wuquinxi | 1.54 ± 0.24 | 1.81 ± 0.43 |  | 55.12 ± 10.76 | 68.07 ± 15.77 |  |  |  |  |  |  |  | 63.92 ± 10.54 | 76.14 ± 10.67 |  |  |  |  |
|  |  |  | Usual care | 1.51 ± 0.46 | 1.40 ± 0.13 |  | 53.07 ± 11.12 | 50.54 ± 10.64 |  |  |  |  |  |  |  | 64.51 ± 9.59 | 61.76 ± 10.01 |  |  |  |  |
|  |  |  | Walking exercise | 1.47 ± 0.33 | 1.53 ± 0.44 |  | 54.09 ± 9.63 | 58.68 ± 13.51 |  |  |  |  |  |  |  | 65.14 ± 9.76 | 68.96 ± 11.87 |  |  |  |  |
|  |  | Xiao (2015)/China, Beijing | Liuzijue |  |  |  |  |  |  |  |  |  |  |  |  |  |  |  | 301.0 ± 10.9 | 321.5 ± 15.5 |  |
|  |  |  | Pursed-lip breathing and walking exercise |  |  |  |  |  |  |  |  |  |  |  |  |  |  |  | 301.0 ± 13.5 | 310.9 ± 14.4 |  |
|  |  | Wu (2018)/China, Shanghai (b) | Liuzijue | 1.55 ± 0.55 | 1.66 ± 0.7 |  | 55 ± 17 | 63 ± 22 |  |  |  |  | 74 ± 19 | 76 ± 20 |  | 62 ± 20 | 62 ± 17 |  | 435.63 ± 66.27 | 485.75 ± 68.91 |  |
|  |  |  | Usual care | 1.48 ± 0.55 | 1.34 ± 0.42 |  | 55 ± 16 | 54 ± 16 |  |  |  |  | 73 ± 19 | 68 ± 17 |  | 59 ± 9 | 59 ± 14 |  | 408.71 ± 61.88 | 410.41 ± 64.35 |  |
|  |  |  | Liuzijue and elastic band exercise | 1.43 ± 0.36 | 1.55 ± 0.5 |  | 53 ± 15 | 60 ± 18 |  |  |  |  | 75 ± 17 | 77 ± 20 |  | 59 ± 9 | 61 ± 16 |  | 432.06 ± 52.78 | 478.91 ± 48.93 |  |
|  |  | Li (2018)/China, Shanghai | Liuzijue |  |  |  | 55.50 ± 16.8 | 63.06 ± 21.6 |  |  |  |  |  |  |  | 56.93 ± 12.8 | 62.28 ± 16.4 |  | 435.41 ± 64.18 | 483.62 ± 67.30 |  |
|  |  |  | Usual care |  |  |  | 58.49 ± 19.4 | 57.13 ± 18.3 |  |  |  |  |  |  |  | 60.78 ± 10.3 | 58.07 ± 13.0 |  | 417.46 ± 64.05 | 418.71 ± 66.19 |  |
|  |  | Chen (2008)/China, Fujian | Liuzijue | 1.03 ± 0.23 | 1.19 ± 0.26 |  | 41.13 ± 15.74 | 48.33 ± 11.82 |  |  |  |  |  |  |  | 53.76 ± 10.28 | 56.95 ± 6.81 |  |  |  |  |
|  |  |  | Usual care | 0.94 ± 0.26 | 0.95 ± 0.31 |  | 41.37 ± 16.77 | 38.21 ± 16.73 |  |  |  |  |  |  |  | 53.32 ± 9.94 | 50.05 ± 11.12 |  |  |  |  |
|  |  | Yan (2020)/China,Taian | Enhanced Liuzijue |  |  |  | 52.42 ± 7.00 | 54.12 ± 6.97 |  |  |  |  |  |  |  | 48.37 ± 3.80 | 53.77 ± 4.66 |  | 378.81 ± 16.92 | 399.35 ± 16.89 |  |
|  |  |  | Usual care |  |  |  | 52.99 ± 6.88 | 54.23 ± 6.71 |  |  |  |  |  |  |  | 48.78 ± 4.26 | 52.47 ± 5.11 |  | 375.66 ± 14.54 | 387.80 ± 13.68 |  |
|  |  |  | Liuzijue and herbal medicine |  |  |  | 51.57 ± 7.62 | 53.36 ± 7.38 |  |  |  |  |  |  |  | 49.11 ± 4.94 | 53.87 ± 5.14 |  | 379.77 ± 16.11 | 393.89 ± 16.55 |  |
|  |  |  | Enhanced Liuzijue and herbal medicine |  |  |  | 51.41 ± 7.97 | 54.57 ± 7.08 |  |  |  |  |  |  |  | 48.04 ± 3.93 | 54.84 ± 4.15 |  | 382.57 ± 17.89 | 407.88 ± 15.17 |  |
|  |  | Deng (2020)/China, Fuzhou | Liuzijue | 1.28 ± 0.56 | 1.32 ± 0.54 |  | 57.66 ± 26.17 | 59.62 ± 25.49 |  |  |  |  |  |  |  | 58.73 ± 14.01 | 60.72 ± 14.72 |  |  |  |  |
|  |  |  | Usual care | 1.15 ± 0.46 | 1.14 ± 0.45 |  | 54.24 ± 23.24 | 53.27 ± 22.33 |  |  |  |  |  |  |  | 56.14 ± 14.28 | 56.59 ± 15.48 |  |  |  |  |
|  |  | Deng (2018)/China, Fuzhou | Liuzijue |  |  |  |  |  |  |  |  |  |  |  |  |  |  |  | 315.11 ± 50.82 | 374.39 ± 52.34 |  |
|  |  |  | Full-body breathing |  |  |  |  |  |  |  |  |  |  |  |  |  |  |  | 299.35 ± 50.38 | 334.08 ± 57.35 |  |
|  |  | Deng (2009)/China, Fuzhou | Enhanced Liuzijue | 1.03 ± 0.32 | 1.07 ± 0.31 |  | 38.00 ± 10.64 | 38.24 ± 8.11 |  |  |  |  |  |  |  | 55.64 ± 7.37 | 57.65 ± 7.87 |  | 310.71 ± 52.20 | 360.65 ± 53.24 |  |
|  |  |  | Full-body breathing | 1.06 ± 0.39 | 1.07 ± 0.40 |  | 37.8 ± 9.72 | 38.27 ± 10.62 |  |  |  |  |  |  |  | 58.24 ± 8.23 | 57.22 ± 7.71 |  | 302.62 ± 50.32 | 337.59 ± 69.02 |  |
|  |  | He (2019)/China, Beijing | Liuzijue |  |  |  | 62.03 ± 17.64 | 63.99 ± 18.43 |  | 2.61 ± 0.87 | 2.68 ± 091 |  |  |  |  | 56.74 ± 9.23 | 58.40 ± 10.26 |  |  |  |  |
|  |  |  | Usual care |  |  |  | 57.08 ± 21.44 | 57.08 ± 23.00 |  | 2.40 ± 0.84 | 2.32 ± 0.91 |  |  |  |  | 55.96 ± 9.97 | 55.73 ± 10.98 |  |  |  |  |
|  |  | Lan (2016)/China, Luzhou | Liuzijue |  |  |  | 55.05 ± 6.49 | 71.38 ± 5.13 |  |  |  |  |  |  |  | 55.72 ± 8.14 | 74.38 ± 4.27 |  |  |  |  |
|  |  |  | Usual care |  |  |  | 54.60 ± 6.71 | 61.55 ± 5.66 |  |  |  |  |  |  |  | 55.41 ± 8.43 | 62.68 ± 5.11 |  |  |  |  |
|  |  | Zhang (2021)/China, Tianjin | Liuzijue |  |  |  |  |  |  |  |  |  |  |  |  |  |  |  | 433.62 ± 106.548 | 478.38 ± 66.438 |  |
|  |  |  | Usual care |  |  |  |  |  |  | Median (Q1,Q3) 1.53(1.39,1.66) | Median (Q1,Q3) 2.55(2.30,2.88) |  |  |  |  |  |  |  | 415.83 ± 83.757 | 414.60 ± 71.753 |  |
|  |  | Lu (2021)/China, Fujian province | Liuzijue | Median (Q1, Q3) 1.25 (1.17,1.36) | Median (Q1, Q3) 1.53 (1.39,1.66) |  |  |  |  | Median (Q1, Q3) 1.43 (1.29,1.50) | Median (Q1, Q3) 2.51 (2.23,2.77) |  |  |  |  |  |  |  |  |  |  |
|  |  |  | Usual care | Median (Q1, Q3) 1.27 (1.20,1.37) | Median (Q1, Q3) 1.43 (1.29,1.50) |  |  |  |  |  |  |  |  |  |  |  |  |  |  |  |  |
|  |  | Liu (2017)/China, Shanghai | Liuzijue on land |  |  |  |  |  |  |  |  |  |  |  |  |  |  |  | 440.47 ± 61.83 | 449.80 ± 58.11 |  |
|  |  |  | Usual care |  |  |  |  |  |  |  |  |  |  |  |  |  |  |  | 418.16 ± 63.26 | 413.76 ± 65.67 |  |
|  |  |  | Liuzijue in the water |  |  |  |  |  |  |  |  |  |  |  |  |  |  |  | 395.57 ± 63.93 | 486.10 ± 78.52 |  |
|  |  | Hu (2021)/China, Shanghai | Liuzijue on land | 1.30 ± 0.32 | 1.33 ± 0.31 |  | 50.09 ± 11.61 | 53.80 ± 9.90 |  |  |  |  |  |  |  | 57.84 ± 8.84 | 61.91 ± 12.18 |  | 548.33 ± 62.80 | 560.56 ± 62.38 |  |
|  |  |  | Usual care | 1.23 ± 0.29 | 1.13 ± 0.31 |  | 50.02 ± 12.48 | 46.84 ± 11.95 |  |  |  |  |  |  |  | 56.81 ± 8.55 | 56.16 ± 14.55 |  | 537.50 ± 58.57 | 532.75 ± 54.69 |  |
|  |  |  | Liuzijue in the water | 1.17 ± 0.34 | 1.40 ± 0.34 |  | 49.36 ± 10.42 | 57.22 ± 9.85 |  |  |  |  |  |  |  | 54.77 ± 10.49 | 62.18 ± 11.79 |  | 528.33 ± 82.62 | 579.44 ± 76.96 |  |
|  |  | Shen (2017)/China, Shanghai | Liuzijue | 1.85 ± 0.38 | 1.69 ± 0.40 |  |  |  |  | 2.76 ± 0.60 | 2.64 ± 0.59 |  |  |  |  |  |  |  |  |  |  |
|  |  |  | Usual care | 1.91 ± 0.40 | 1.65 ± 0.40 |  |  |  |  | 2.91 ± 0.65 | 2.73 ± 0.67 |  |  |  |  |  |  |  |  |  |  |
|  |  | Ji (2019)/China, Shanghai | Liuzijue |  |  |  | 40.32 ± 12.64 | 41.37 ± 13.97 |  |  |  |  |  |  |  | 50.36 ± 11.21 | 49.04 ± 12.42 |  |  |  |  |
|  |  |  | Usual care |  |  |  | 46.85 ± 11.80 | 46.11 ± 12.46 |  |  |  |  |  |  |  | 52.12 ± 12.55 | 52.87 ± 12.28 |  |  |  |  |
|  |  | Qu (2019)/China, Taizhou | Liuzijue |  |  |  |  |  |  |  |  |  |  |  |  |  |  |  | 284.6 ± 96.6 | 394.8 ± 89.4 |  |
|  |  |  | Full-body breathing |  |  |  |  |  |  |  |  |  |  |  |  |  |  |  | 275.6 ± 95.5 | 311.5 ± 86.9 |  |
|  |  | Li (2011)/China, Fuzhou | Liuzijue | 1.12 ± 0.33 | 1.30 ± 0.32 |  | 47.00 ± 12.88 | 50.47 ± 12.23 |  |  |  |  |  |  |  | 56.67 ± 13.19 | 56.50 ± 10.40 |  |  |  |  |
|  |  |  | Full-body breathing | 1.28 ± 0.36 | 1.33 ± 0.35 |  | 53.30 ± 12.47 | 53.77 ± 12.12 |  |  |  |  |  |  |  | 54.50 ± 10.85 | 52.90 ± 11.39 |  |  |  |  |
|  |  | Li (2018)/China, Beijing | Liuzijue |  |  |  | 57.00 ± 4.71 | 72.47 ± 3.42 |  | 2.34 ± 0.14 | 2.40 ± 0.13 |  |  |  |  | 59.47 ± 4.91 | 63.53 ± 3.23 |  |  |  |  |
|  |  |  | Usual care |  |  |  | 56.33 ± 5.30 | 55.40 ± 6.01 |  | 2.33 ± 0.18 | 2.33 ± 0.17 |  |  |  |  | 58.87 ± 4.02 | 57.67 ± 4.90 |  |  |  |  |
|  |  | Yan (2021)/China, Lanzhou | Liuzijue |  |  |  | 56.34 ± 15.20 | 64.35 ± 14.75 |  |  |  |  |  |  |  | 57.11 ± 11.23 | 63.19 ± 13.08 |  | 435.26 ± 65.05 | 484.66 ± 59.38 |  |
|  |  |  | Usual care |  |  |  | 58.46 ± 15.21 | 57.67 ± 14.22 |  |  |  |  |  |  |  | 60.59 ± 10.54 | 59.06 ± 12.35 |  | 418.02 ± 63.59 | 419.28 ± 66.05 |  |
|  |  | Wang (2013)/China, Shanghai | Enhanced Liuzijue | 1.10 ± 0.27 | 1.07 ± 0.26 |  |  |  |  |  |  |  |  |  |  | 52.84 ± 9.54 | 52.28 ± 9.36 |  |  |  |  |
|  |  |  | Diaphragmatic and pursed-lip breathing | 1.12 ± 0.27 | 1.08 ± 0.25 |  |  |  |  |  |  |  |  |  |  | 52.91 ± 8.66 | 52.12 ± 8.36 |  |  |  |  |
|  |  | Quan (2021)/China, Tianjin | Enhanced liuzijue |  |  |  | 49.75 ± 11.14 | 48.18 ± 12.51 |  |  |  |  |  |  |  | 57.50 ± 11.88 | 59.77 ± 10.70 |  |  |  |  |
|  |  |  | Usual care |  |  |  | 48.04 ± 11.40 | 40.71 ± 10.23 |  |  |  |  |  |  |  | 57.58 ± 10.40 | 55.08 ± 10.39 |  |  |  |  |
|  |  |  | Diaphragmatic and pursed-lip breathing |  |  |  | 49.41 ± 11.47 | 45.16 ± 16.62 |  |  |  |  |  |  |  | 57.89 ± 9.22 | 56.80 ± 11.13 |  |  |  |  |
|  |  | Liu (2021)/China, Shanghai | Liuzijue on land |  |  |  |  |  |  |  |  |  |  |  |  |  |  |  | 432.20 ± 65.97 | 451.00 ± 62.09 |  |
|  |  |  | Usual care |  |  |  |  |  |  |  |  |  |  |  |  |  |  |  | 422.75 ± 61.31 | 417.16 ± 63.36 |  |
|  |  |  | Liuzijue in the water |  |  |  |  |  |  |  |  |  |  |  |  |  |  |  | 395.57 ± 63.93 | 486.07 ± 78.52 |  |
|  | Yijinging (易筋经) | Zhang (2016)/China, Jiangsu province | Yijinging | 1.70 ± 0.55 | 2.12 ± 0.37 |  | 59.12 ± 4.13 | 63.16 ± 6.54 |  |  |  |  |  |  |  | 60.39 ± 4.84 | 63.09 ± 6.89 |  | 294.34 ± 7.89 | 324.23 ± 10.67 |  |
|  |  |  | Usual care | 1.65 ± 0.43 | 1.61 ± 0.38 |  | 58.11 ± 4.37 | 57.13 ± 6.27 |  |  |  |  |  |  |  | 59.15 ± 5.79 | 58.32 ± 7.18 |  | 294.18 ± 9.12 | 292.95 ± 8.89 |  |
|  |  |  | Self-management exercise intervention | 1.68 ± 0.43 | 1.83 ± 0.53 |  | 57.39 ± 5.15 | 59.16 ± 7.12 |  |  |  |  |  |  |  | 58.45 ± 5.15 | 59.37 ± 5.13 |  | 295.47 ± 10.29 | 302.25 ± 7.74 |  |
|  |  | Gao (2015)/China, Suzhou | Yijinjing | 1.76 ± 0.86 | 2.44 ± 0.68 |  | 69.84 ± 25.80 | 79.45 ± 15.07 |  |  |  |  |  |  |  | 62.61 ± 16.69 | 65.15 ± 14.09 |  | 375.7 ± 95.89 | 422.1 ± 76.14 |  |
|  |  |  | Diaphragmatic breathing | 1.70 ± 0.90 | 1.91 ± 0.56 |  | 64.23 ± 17.53 | 66.94 ± 15.86 |  |  |  |  |  |  |  | 58.29 ± 13.14 | 61.85 ± 12.69 |  | 376.9 ± 73.33 | 400.2 ± 64.88 |  |
|  | Tai chi (太极) | Zhu (2018)/China, Changsha | Tai chi |  |  |  | 35.11 ± 13.74 | 40.16 ± 15.51 |  |  |  |  |  |  |  |  |  |  | 450.34 ± 59.23 | 489.09 ± 59.55 |  |
|  |  |  | Usual care |  |  |  | 40.77 ± 15.60 | 41.43 ± 13.82 |  |  |  |  |  |  |  |  |  |  | 420.28 ± 108.08 | 395.76 ± 115.22 |  |
|  |  | Zhang (2014)/China, Jinan | Tai chi | 1.36 ± 0.24 | 1.38 ± 0.27 |  | 56.71 ± 7.62 | 57.54 ± 7.35 |  |  |  |  |  |  |  | 45.13 ± 11.31 | 48.01 ± 10.13 |  | 243.12 ± 39.54 | 292.13 ± 56.91 |  |
|  |  |  | Usual care | 1.37 ± 0.24 | 1.34 ± 0.29 |  | 57.81 ± 6.94 | 57.16 ± 7.23 |  |  |  |  |  |  |  | 48.26 ± 10.12 | 48.62 ± 11.32 |  | 238.30 ± 45.13 | 269.14 ± 50.23 |  |
|  |  |  | Diaphragmatic breathing | 1.34 ± 0.25 | 1.37 ± 0.23 |  | 56.72 ± 8.11 | 56.94 ± 7.85 |  |  |  |  |  |  |  | 46.24 ± 12.12 | 45.34 ± 13.16 |  | 239.10 ± 42.31 | 299.16 ± 54.25 |  |
|  |  |  | Tai chi + iaphragmatic breathing | 1.35 ± 0.28 | 1.37 ± 0.28 |  | 55.62 ± 7.53 | 56.41 ± 7.13 |  |  |  |  |  |  |  | 46.16 ± 11.84 | 48.12 ± 10.57 |  | 242.13 ± 41.72 | 314.13 ± 50.65 |  |
|  |  | Zhang (2012)/China, Beijing | Tai chi + pursed-lip breathing | 1.33 ± 0.28 | 1.38 ± 0.24 |  | 51.7 ± 8.5 | 53.9 ± 8.1 |  |  |  |  |  |  |  | 45 ± 8.8 | 46 ± 8.5 |  | 230 ± 49.5 | 302 ± 58.2 |  |
|  |  |  | Usual care | 1.34 ± 0.25 | 1.29 ± 0.21 |  | 51.9 ± 7.5 | 49.6 ± 8.2 |  |  |  |  |  |  |  | 46 ± 10.1 | 43 ± 9.3 |  | 235 ± 45.6 | 253 ± 45.5 |  |
|  |  |  | Pursed-lip breathing | 1.35 ± 0.24 | 1.38 ± 0.25 |  | 52.5 ± 8.7 | 53.6 ± 8.6 |  |  |  |  |  |  |  | 47 ± 7.9 | 46 ± 6.8 |  | 237 ± 47.3 | 280 ± 52.3 |  |
|  |  | Yeh (2020)/USA, Boston | Tai chi |  |  |  |  |  |  |  |  |  |  |  |  |  |  |  |  |  | 35.3 ± 118.6 |
|  |  |  | Usual care |  |  |  |  |  |  |  |  |  |  |  |  |  |  |  |  |  | 30.5 ± 121.6 |
|  |  | Wang (2014)/China, Yingde & Zhengzhou | Tai chi |  |  |  | 41.80 ± 14.35 | 41.39 ± 10.63 |  |  |  |  |  |  |  |  |  |  | 420.15 ± 35.78 | 479.12 ± 34.32 |  |
|  |  |  | Usual care |  |  |  | 43.44 ± 11.66 | 48.08 ± 7.71 |  |  |  |  |  |  |  |  |  |  | 424.34 ± 50.56 | 429.07 ± 51.29 |  |
|  |  | Wang (2019)/China, Zunyi | Tai chi | 1.18 ± 0.04 | 1.39 ± 0.05 |  | 55.46 ± 11.47 | 59.81 ± 15.09 |  |  |  |  |  |  |  | 53.25 ± 16.34 | 56.43 ± 17.21 |  | 451.71 ± 61.39 | 488.57 ± 61.57 |  |
|  |  |  | Usual care | 1.19 ± 0.10 | 1.08 ± 0.47 |  | 62.55 ± 20.18 | 60.52 ± 17.52 |  |  |  |  |  |  |  | 56.71 ± 18.17 | 55.47 ± 20.34 |  | 455.51 ± 71.72 | 431.87 ± 71.17 |  |
|  |  | Polkey (2018)/China, Meizhou | Tai chi | 1.1 ± 0.4 | 1.2 ± 0.42 |  |  |  |  | 2.4 ± 0.6 | 2.5 ± 0.6 |  |  |  |  |  |  |  | 561 ± 57.9 | 580 ± 52.5 |  |
|  |  |  | PR | 1.2 ± 0.5 | 1.3 ± 0.5 |  |  |  |  | 2.4 ± 0.6 | 2.4 ± 0.7 |  |  |  |  |  |  |  | 541 ± 67.0 | 564 ± 68.0 |  |
|  |  | Niu (2014)/China, Changsha | Tai chi | 1.21 ± 0.10 | 1.43 ± 0.08 |  | 41.9 ± 5.50 | 47.6 ± 4.76 |  |  |  |  |  |  |  |  |  |  | 431 ± 22.8 | 476 ± 15.0 |  |
|  |  |  | Usual care | 1.18 ± 0.09 | 1.18 ± 0.09 |  | 43.7 ± 5.16 | 42.6 ± 5.46 |  |  |  |  |  |  |  |  |  |  | 422 ± 20.0 | 416 ± 22.5 |  |
|  |  | Ng (2014)/China, Hong Kong | Tai chi + PR | 1.10 ± 0.45 | 1.20 ± 0.50 |  | 56.38 ± 23.81 | 61.71 ± 26.35 |  | 2.05 ± 0.66 | 2.20 ± 0.75 |  |  |  |  |  |  |  | 312.1 ± 64.15 | 339.5 ± 69.43 |  |
|  |  |  | PR | 1.23 ± 0.45 | 1.31 ± 0.55 |  | 63.1 ± 22.11 | 67.0 ± 26.89 |  | 2.26 ± 0.80 | 2.33 ± 0.94 |  |  |  |  |  |  |  | 320.2 ± 71.86 | 341.0 ± 73.81 |  |
|  |  | Yeh (2010)/USA, Boston | Tai chi |  |  |  |  |  |  |  |  |  |  |  |  | median (range) 73 (48-87) | median (range) 69 (53-85) |  | median (range) 401 (240-575) | median (range) 428 (379-624) |  |
|  |  |  | Usual care |  |  |  |  |  |  |  |  |  |  |  |  | median (range) 54 (42-73) | median (range) 54 (43-72) |  | median (range) 422 (121-526) | median (range) 381 (121-522) |  |
|  |  | Moy (2021)/USA, Boston | Tai chi |  |  |  |  |  |  |  |  |  |  |  |  |  |  |  | 324.7 ± 123.4 |  | +6.8 ± 53.3 |
|  |  |  | Usual care |  |  |  |  |  |  |  |  |  |  |  |  |  |  |  | 369.7 ± 104.7 |  | -5.1 ± 41.2 |
|  |  | Kantatong (2020)/Thailand, Chiang Mai | Tai chi | 1.25 ± 0.45 | 1.27 ± 0.41 |  |  |  |  | 2.15 ± 0.67 | 2.16 ± 0.68 |  |  |  |  |  |  |  | 368.72 ± 77.38 | 442.28 ± 60.85 |  |
|  |  |  | Usual care | 1.36 ± 0.41 | 1.22 ± 0.39 |  |  |  |  | 2.28 ± 0.66 | 2.27 ± 0.60 |  |  |  |  |  |  |  | 387.12 ± 98.89 | 348.64 ± 93.26 |  |
|  |  | Du (2013)/China, Zhangjiakou | Tai chi |  |  |  | 73.36 ± 6.33 | 79.14 ± 5.36 |  |  |  |  |  |  |  | 55.01 ± 4.77 | 61.05 ± 4.82 |  | 291.00 ± 13.75 | 300.78 ± 10.79 |  |
|  |  |  | Usual care |  |  |  | 72.97 ± 6.46 | 71.26 ± 6.38 |  |  |  |  |  |  |  | 55.54 ± 4.75 | 54.39 ± 5.04 |  | 291.22 ± 13.84 | 290.45 ± 9.64 |  |
|  |  |  | Exercise |  |  |  | 74.28 ± 7.39 | 75.16 ± 7.15 |  |  |  |  |  |  |  | 54.04 ± 4.65 | 54.06 ± 5.28 |  | 288.50 ± 11.51 | 291.17 ± 11.91 |  |
|  |  | Chan (2013)/China, Hong kong | Tai chi | 0.89 ± 0.38 | 0.99 ± 0.42 |  |  |  |  | 1.97 ± 0.62 | 2.16 ± 0.63 |  |  |  |  |  |  |  | 297.91 ± 68.53 | 349.41 ± 70.69 |  |
|  |  |  | Usual care | 0.89 ± 0.39 | 0.84 ± 0.31 |  |  |  |  | 1.82 ± 0.58 | 1.69 ± 0.48 |  |  |  |  |  |  |  | 289.75 ± 72.97 | 297.09 ± 84.25 |  |
|  |  |  | Diaphragmatic and pursed-lip breathing + walking exercise | 0.91 ± 0.39 | 0.94 ± 0.42 |  |  |  |  | 1.84 ± 0.52 | 1.95 ± 0.62 |  |  |  |  |  |  |  | 284.64 ± 79.11 | 298.07 ± 87.74 |  |
|  |  | Zhang(2019)/China, Chongqing | Tai chi | (mL/s) 2131 ± 76 | (mL/s) 2731 ± 87 |  | 53.51 ± 5.42 | 82.31 ± 5.12 |  | (mL/s) 2232 ± 81 | (mL/s) 2832 ± 91 |  |  |  |  |  |  |  |  |  |  |
|  |  |  | Pursed-lip breathing | (mL/s) 2137 ± 70 | (mL/s) 2387 ± 77 |  | 53.64 ± 5.54 | 68.52 ± 6.04 |  | (mL/s) 2238 ± 87 | (mL/s) 2508 ± 80 |  |  |  |  |  |  |  |  |  |  |
|  |  | Cui (2016)/China, Zhangjiakou | Tai chi + non-invasive ventilators | 1.34 ± 0.27 | 1.41 ± 0.27 |  | 55.60 ± 7.51 | 56.39 ± 7.12 |  |  |  |  |  |  |  | 46.15 ± 11.82 | 48.11 ± 10.61 |  | 242.10 ± 41.68 | 315.12 ± 50.58 |  |
|  |  |  | Non-invasive ventilators | 1.35 ± 0.23 | 1.39 ± 0.29 |  | 56.69 ± 7.58 | 57.53 ± 7.32 |  |  |  |  |  |  |  | 45.12 ± 11.29 | 47.98 ± 10.10 |  | 242.11 ± 39.61 | 292.08 ± 57.34 |  |
|  |  | Li (2019)/China, Fenyang | Tai chi + diaphragmatic and pursed-lip breathing | 1.25 ± 0.88 | 1.95 ± 0.90 |  |  |  |  | 2.06 ± 0.36 | 2.58 ± 0.44 |  |  |  |  | 53.39 ± 15.29 | 71.44 ± 14.28 |  |  |  |  |
|  |  |  | Diaphragmatic and pursed-lip breathing | 1.24 ± 0.92 | 1.54 ± 0.93 |  |  |  |  | 2.12 ± 0.38 | 2.36 ± 0.42 |  |  |  |  | 54.12 ± 16.05 | 62.56 ± 15.09 |  |  |  |  |
|  |  | Li (2016)/China, Yantai | Tai chi + diaphragmatic and pursed-lip breathing | 1.33 ± 0.53 | 1.45 ± 0.60 |  | 46.0 ± 16.5 | 50.3 ± 16.9 |  |  |  |  |  |  |  | 47.4 ± 15.5 | 49.7 ± 16.2 |  | 456 ± 96 | 526 ± 88 |  |
|  |  |  | Diaphragmatic and pursed-lip breathing | 1.32 ± 0.50 | 1.36 ± 0.57 |  | 46.1 ± 16.5 | 48.3 ± 16.6 |  |  |  |  |  |  |  | 48.2 ± 14.6 | 49.8 ± 14.3 |  | 448 ± 92 | 468 ± 90 |  |
|  |  | Li (2012)/China, Jinan | Tai chi + diaphragmatic and pursed-lip breathing | 1.40 ± 0.60 | 1.89 ± 0.64 |  |  |  |  | 2.71 ± 0.69 | 3.21 ± 0.79 |  |  |  |  | 52.05 ± 3.40 | 59.24 ± 2.92 |  |  |  |  |
|  |  |  | Diaphragmatic and pursed-lip breathing | 1.41 ± 0.62 | 1.87 ± 0.66 |  |  |  |  | 2.69 ± 0.67 | 3.16 ± 0.70 |  |  |  |  | 52.54 ± 2.78 | 59.37 ± 3.14 |  |  |  |  |
|  |  | Pan (2018)/China, Chengdu | Tai chi + health education | 1.42 ± 0.11 | 1.44 ± 0.11 |  | 52.80 ± 3.12 | 54.70 ± 3.86 |  | 2.65 ± 0.16 | 2.73 ± 0.19 |  | 78.05 ± 3.89 | 74.80 ± 4.91 |  |  |  |  | 461.40 ± 18.06 | 518.20 ± 21.74 |  |
|  |  |  | Usual care | 1.61 ± 0.13 | 1.48 ± 0.11 |  | 52.05 ± 3.27 | 54.14 ± 3.84 |  | 2.71 ± 0.14 | 2.70 ± 0.14 |  | 76.43 ± 2.98 | 77.05 ± 3.24 |  |  |  |  | 499.81 ± 14.57 | 518.52 ± 19.26 |  |
|  |  | Ren (2017)/China, Beijing | Tai chi + health education | 0.90 ± 0.22 | 1.72 ± 0.30 |  |  |  |  | 1.53 ± 0.24 | 2.23 ± 0.34 |  |  |  |  | 40.23 ± 4.14 | 55.00 ± 2.34 |  | 541.00 ± 83.10 | 595.20 ± 82.03 |  |
|  |  |  | PR + health education | 0.88 ± 0.41 | 1.43 ± 0.31 |  |  |  |  | 1.53 ± 0.25 | 1.95 ± 0.23 |  |  |  |  | 39.91 ± 4.23 | 45.00 ± 3.43 |  | 547.23 ± 71.54 | 570.12 ± 62.03 |  |
|  |  | He (2019)/China, Jinan | Tai chi + health education | 1.49 ± 0.08 | 2.11 ± 0.07 |  | 67.43 ± 1.21 | 80.49 ± 1.27 |  | 2.24 ± 0.10 | 2.63 ± 0.09 |  |  |  |  |  |  |  | 322.5 ± 7.62 | 458.2 ± 12.28 |  |
|  |  |  | Usual care | 1.63 ± 0.05 | 1.36 ± 0.04 |  | 68.12 ± 1.03 | 56.16 ± 1.88 |  | 2.40 ± 0.08 | 2.49 ± 0.09 |  |  |  |  |  |  |  | 326.2 ± 11.29 | 343.3 ± 11.14 |  |
|  | Baduanjin (八段锦) | Lu (2015)/China, Beijing | Baduanjin |  |  |  |  |  |  |  |  |  |  |  |  |  |  |  | 395.60 ± 20.06 | 399.24 ± 18.54 |  |
|  |  |  | Usual care |  |  |  |  |  |  |  |  |  |  |  |  |  |  |  | 406.29 ± 16.92 | 404.85 ± 15.77 |  |
|  |  | Feng (2009)/China, Guangzhou | Baduanjin | 1.52 ± 0.45 | 1.63 ± 0.71 |  | 56.98 ± 6.41 | 61.19 ± 6.97 |  | 2.41 ± 0.46 | 2.47 ± 0.59 |  |  |  |  | 61.49 ± 8.02 | 65.88 ± 10.49 |  | 254.12 ± 40.12 | 281.46 ± 54.06 |  |
|  |  |  | Usual care | 1.53 ± 0.31 | 1.55 ± 0.86 |  | 55.85 ± 6.23 | 57.17 ± 7.02 |  | 2.39 ± 0.51 | 2.42 ± 0.81 |  |  |  |  | 62.03 ± 7.87 | 63.31 ± 9.84 |  | 255.39 ± 41.56 | 257.65 ± 45.36 |  |
|  |  | Huang (2017)/China, Nanjing | Baduanjin | 1.09 ± 0.34 | 1.56 ± 0.13 |  | 49.23 ± 9.21 | 57.76 ± 9.01 |  |  |  |  |  |  |  | 52.04 ± 8.97 | 58.45 ± 10.10 |  |  |  |  |
|  |  |  | Usual care | 1.07 ± 0.12 | 1.12 ± 0.11 |  | 50.28 ± 8.67 | 51.93 ± 9.12 |  |  |  |  |  |  |  | 53.88 ± 9.77 | 54.06 ± 9.03 |  |  |  |  |
|  |  | Yin (2013)/China, Guangzhou | Baduanjin |  |  |  |  |  |  |  |  |  |  |  |  |  |  |  | 356.58 ± 114.16 | 385.62 ± 99.13 |  |
|  |  |  | Diaphragmatic breathing |  |  |  |  |  |  |  |  |  |  |  |  |  |  |  | 350.56 ± 115.76 | 369.13 ± 111.67 |  |
|  |  | Chen (2015)/China, Fuzhou | Baduanjin + health education |  |  |  |  |  |  |  |  |  |  |  |  |  |  |  | 349.00 ± 27.94 | 376.04 ± 25.68 |  |
|  |  |  | Usual care |  |  |  |  |  |  |  |  |  |  |  |  |  |  |  | 346.23 ± 31.57 | 355.54 ± 26.65 |  |
|  |  | Zhu (2014)/China, Changsha | Baduanjin | 0.93 ± 0.14 | 1.38 ± 0.18 |  | 48.20 ± 11.28 | 58.31 ± 13.23 |  | 1.86 ± 0.89 | 2.28 ± 1.34 |  |  |  |  | 45.23 ± 10.28 | 57.00 ± 10.78 |  | 342.18 ± 30.93 | 398.17 ± 43.86 |  |
|  |  |  | Usual care | 0.95 ± 10.15 | 0.96 ± 0.17 |  | 48.45 ± 10.34 | 47.45 ± 11.18 |  | 1.82 ± 1.12 | 1.83 ± 1.08 |  |  |  |  | 46.12 ± 11.17 | 45.94 ± 10.28 |  | 343.27 ± 31.45 | 342.18 ± 30.93 |  |
|  |  | Ng (2011)/China, Hong Kong | Baduanjin |  |  |  |  |  |  |  |  |  |  |  |  |  |  |  | 310.78 ± 13.03 | 338.53 ± 13.42 |  |
|  |  |  | Pursed-lip breathing + walking exercise |  |  |  |  |  |  |  |  |  |  |  |  |  |  |  | 310.15 ± 13.03 | 320.80 ± 13.42 |  |
|  |  | Xu (2010)/China, Yunyang | Baduanjin |  |  |  |  |  |  |  |  |  |  |  |  |  |  |  | 387.48 ± 34.50 | 460.85 ± 35.80 |  |
|  |  |  | Usual care |  |  |  |  |  |  |  |  |  |  |  |  |  |  |  | 385.42 ± 35.80 | 385.82 ± 39.80 |  |
|  |  |  | PR |  |  |  |  |  |  |  |  |  |  |  |  |  |  |  | 386.42 ± 35.50 | 458.85 ± 40.80 |  |
|  |  |  | Baduanjin + PR |  |  |  |  |  |  |  |  |  |  |  |  |  |  |  | 385.82 ± 28.50 | 485.85 ± 42.80 |  |
|  |  | Huang (2016)/China, Dongguan | Baduanjin + diaphragmatic and pursed-lip breathing | 1.02 ± 0.42 | 1.27 ± 0.64 |  | 41.07 ± 16.88 | 50.20 ± 17.35 |  | 2.01 ± 0.56 | 2.68 ± 0.55 |  |  |  |  | 44.03 ± 11.23 | 49.80 ± 11.50 |  | points 1.02 ± 0.33 | points 1.77 ± 0.42 |  |
|  |  |  | Diaphragmatic and pursed-lip breathing | 1.03 ± 0.21 | 1.39 ± 0.53 |  | 42.25 ± 16.45 | 58.20 ± 17.83 |  | 2.11 ± 0.65 | 2.98 ± 0.70 |  |  |  |  | 46.01 ± 12.25 | 55.97 ± 13.20 |  | points 1.02 ± 0.33 | points 2.49 ± 0.67 |  |
|  |  | Chen (2017)/China, Shenyang | Baduanjin + health education | 1.15 ± 0.52 | 1.09 ± 0.51 |  |  |  |  | 2.32 ± 0.68 | 2.29 ± 0.75 |  |  |  |  |  |  |  |  |  |  |
|  |  |  | Usual care | 1.11 ± 0.79 | 1.06 ± 0.47 |  |  |  |  | 2.47 ± 0.52 | 2.51 ± 0.61 |  |  |  |  |  |  |  |  |  |  |
|  |  | Chen (2015)/China, Chengdu | Baduanjin |  |  |  | 52.48 ± 16.72 | 62.67 ± 20.12 |  |  |  |  |  |  |  | 59.85 ± 10.13 | 67.11 ± 12.89 |  |  |  |  |
|  |  |  | Usual care |  |  |  | 53.43 ± 18.74 | 57.13 ± 22.26 |  |  |  |  |  |  |  | 59.96 ± 10.94 | 63.03 ± 12.65 |  |  |  |  |
|  |  | Zheng (2019)/China, Guangzhou | Baduanjin | 0.86 ± 0.23 | 0.86 ± 0.30 |  | 34.55 ± 9.49 | 35.99 ± 14.14 |  |  |  |  |  |  |  | 48.53 ± 7.83 | 46.80 ± 9.72 |  | 431.88 ± 72.88 | 413.31 ± 63.34 |  |
|  |  |  | Usual care | 0.86 ± 0.19 | 0.88 ± 0.27 |  | 33.18 ± 7.5 | 34.08 ± 12.62 |  |  |  |  |  |  |  | 44.47 ± 8.36 | 45.39 ± 7.25 |  | 423.33 ± 89.12 | 411.27 ± 100.68 |  |
|  |  |  | Pursed-lip breathing | 0.84 ± 0.26 | 0.83 ± 0.29 |  | 33.97 ± 10.03 | 33.94 ± 11.37 |  |  |  |  |  |  |  | 42.47 ± 11.29 | 42.49 ± 7.98 |  | 400.50 ± 54.49 | 385.50 ± 67.55 |  |
|  |  | Deng (2014)/China, Fuzhou | Baduanjin + health education | 1.13 ± 0.53 | 1.18 ± 0.55 |  | 42.18 ± 17.99 | 44.30 ± 18.40 |  | 2.42 ± 0.67 | 2.49 ± 0.66 |  |  |  |  | 45.15 ± 12.34 | 45.90 ± 12.61 |  | 349.00 ± 27.94 | 376.04 ± 25.68 |  |
|  |  |  | Usual care | 1.05 ± 0.52 | 1.08 ± 0.55 |  | 43.36 ± 17.56 | 44.31 ± 18.94 |  | 2.22 ± 0.76 | 2.19 ± 0.81 |  |  |  |  | 47.12 ± 13.36 | 50.71 ± 14.40 |  | 346.23 ± 31.57 | 355.54 ± 26.65 |  |
|  |  | Deng (2020)/China, Guangzhou | Baduanjin | 0.71 ± 0.16 | 0.82 ± 0.18 |  | 50.15 ± 20.38 | 51.33 ± 18.52 |  | 1.15 ± 0.47 | 1.28 ± 0.51 |  |  |  |  | 61.87 ± 10.16 | 63.52 ± 12.18 |  | 365.45 ± 67.35 | 422.67 ± 63.48 |  |
|  |  |  | Usual care | 0.69 ± 0.18 | 0.73 ± 0.20 |  | 49.85 ± 16.54 | 50.91 ± 17.96 |  | 1.10 ± 0.56 | 1.21 ± 0.49 |  |  |  |  | 62.15 ± 12.10 | 61.92 ± 11.75 |  | 363.58 ± 66.74 | 379.05 ± 63.46 |  |
|  |  | Zhang (2017)/China, Changchun | Baduanjin |  |  |  | 52.40 ± 9.96 | 53.39 ± 9.94 |  |  |  |  | 57.07 ± 13.92 | 62.28 ± 13.99 |  |  |  |  | 366.47 ± 117.95 | 421.83 ± 135.14 |  |
|  |  |  | Usual care |  |  |  | 52.55 ± 7.76 | 53.47 ± 7.39 |  |  |  |  | 51.92 ± 10.09 | 53.22 ± 9.97 |  |  |  |  | 315.33 ± 111.29 | 338.0 ± 109.04 |  |
|  |  | Sun (2014)/China, Changchun | Baduanjin |  |  |  | 70.86 ± 5.79 | 88.12 ± 5.45 |  |  |  |  |  |  |  |  |  |  | 315.77 ± 36.83 | 428.31 ± 41.06 |  |
|  |  |  | Usual care |  |  |  | 69.78 ± 6.01 | 81.46 ± 5.67 |  |  |  |  |  |  |  |  |  |  | 317.85 ± 35.71 | 330.04 ± 36.62 |  |
|  |  | Liu (2013)/China, Chengdu | Baduanjin |  |  |  | 52.16 ± 16.18 | 60.96 ± 20.40 |  |  |  |  |  |  |  | 61.28 ± 6.95 | 69.31 ± 11.12 |  | 242.75 ± 34.73 | 432.38 ± 50.25 |  |
|  |  |  | Usual care |  |  |  | 53.39 ± 20.19 | 57.53 ± 22.41 |  |  |  |  |  |  |  | 61.77 ± 6.68 | 65.16 ± 10.22 |  | 248.81 ± 33.84 | 383.78 ± 58.59 |  |
|  |  | Ye (2016)/China, Changsha | Baduanjin |  |  |  |  |  |  |  |  |  |  |  |  |  |  |  | 243.46 ± 26.57 | 357.76 ± 28.531 |  |
|  |  |  | Usual care |  |  |  |  |  |  |  |  |  |  |  |  |  |  |  | 247.36 ± 24.89 | 287.43 ± 26.24 |  |
|  |  | Guo (2016)/China, Qingdao | Baduanjin | 1.48 ± 0.58 | 1.91 ± 0.48 |  | 54.84 ± 5.39 | 71.78 ± 6.16 |  | 2.36 ± 0.31 | 2.95 ± 0.56 |  |  |  |  | 58.79 ± 6.76 | 70.25 ± 9.13 |  |  |  |  |
|  |  |  | Usual care | 1.50 ± 0.53 | 1.51 ± 0.52 |  | 56.49 ± 4.16 | 58.67 ± 6.14 |  | 2.50 ± 0.48 | 2.64 ± 0.63 |  |  |  |  | 62.83 ± 6.47 | 66.90 ± 8.35 |  |  |  |  |
|  |  | Liang (2016)/China, Dongguan | Baduanjin | 1.09 ± 0.53 | 1.13 ± 0.54 |  | 42.87 ± 17.40 | 44.18 ± 18.11 |  | 2.33 ± 0.66 | 2.35 ± 0.63 |  |  |  |  | 45.07 ± 13.44 | 45.51 ± 13.38 |  |  |  |  |
|  |  |  | Usual care | 1.11 ± 0.51 | 1.16 ± 0.56 |  | 43.02 ± 17.37 | 44.14 ± 18.06 |  | 2.28 ± 0.75 | 2.20 ± 0.81 |  |  |  |  | 46.35 ± 13.36 | 48.93 ± 12.20 |  |  |  |  |
|  |  | Pan (2016)/China, Dazhou | Baduanjin | 1.33 ± 0.58 | 1.98 ± 0.59 |  | 53.78 ± 13.73 | 64.69 ± 21.01 |  | 2.12 ± 0.69 | 2.69 ± 0.78 |  |  |  |  | 55.07 ± 9.27 | 63.09 ± 13.32 |  | 324.4 ± 86.2 | 453.1 ± 95.3 |  |
|  |  |  | Usual care | 1.35 ± 0.55 | 1.43 ± 0.56 |  | 53.03 ± 12.94 | 55.19 ± 18.37 |  | 2.02 ± 0.74 | 2.13 ± 0.83 |  |  |  |  | 56.64 ± 9.81 | 59.75 ± 10.73 |  | 336.7 ± 85.6 | 366.1 ± 77.4 |  |
|  |  | Pan (2019)/China, Foshan | Baduanjin + PR |  |  |  | 40.14 ± 17.90 | 39.04 ± 18.19 |  |  |  |  | 66.68 ± 18.02 | 64.91 ± 18.35 |  | 66.68 ± 18.02 | 64.91 ± 18.35 |  | 214.47 ± 123.06 | 270.16 ± 121.54 |  |
|  |  |  | Usual care |  |  |  | 46.96 ± 17.34 | 44.59 ± 17.40 |  |  |  |  | 70.80 ± 13.19 | 68.34 ± 13.26 |  | 70.80 ± 13.19 | 68.34 ± 13.26 |  | 220.56 ± 80.76 | 234.34 ± 100.92 |  |
|  |  |  | PR |  |  |  | 41.32 ± 14.75 | 39.65 ± 15.11 |  |  |  |  | 65.93 ± 14.39 | 64.29 ± 13.81 |  | 65.93 ± 14.39 | 64.29 ± 14.81 |  | 221.07 ± 76.38 | 244.63 ± 76.8 |  |
|  |  | Xue (2015)/China, Beijing | Baduanjin |  |  | 0.001 ± 0.169 |  |  | 4.425 ± 16.598 |  |  | 0.186 ± 0.431 |  |  |  |  |  | -4.335 ± 8.874 |  |  |  |
|  |  |  | Usual care |  |  | 0.109 ± 0.346 |  |  | 4.327 ± 11.419 |  |  | 0.254 ± 0.527 |  |  |  |  |  | -1.044 ± 7.636 |  |  |  |
|  |  | Yu (2019)/China, Shiyan | Baduanjin | 1.42 ± 0.12 | 1.75 (sd n.r) |  | 52.42 ± 3.84 | 55.74 ± 3.86 |  | 2.54 ± 0.17 | 2.96 ± 0.17 |  | 71.44 ± 3.85 | 77.81 ± 4.86 |  |  |  |  |  |  |  |
|  |  |  | Usual care | 1.41 ± 0.13 | 1.52 ± 0.12 |  | 52.34 ± 3.27 | 53.82 ± 3.15 |  | 2.52 ± 0.16 | 2.64 ± 0.12 |  | 71.43 ± 2.97 | 74.23 ± 3.9 |  |  |  |  |  |  |  |
|  |  | Wang (2018)/China, Dalian | Baduanjin + health education | 1.34 ± 0.35 | 1.97 ± 0.57 |  | 53.36 ± 8.31 | 64.54 ± 9.41 |  | 2.06 ± 0.55 | 2.68 ± 0.79 |  |  |  |  | 55.13 ± 7.15 | 63.22 ± 8.05 |  | 330.27 ± 25.43 | 362.95 ± 36.43 |  |
|  |  |  | Usual care | 1.35 ± 0.31 | 1.61 ± 0.42 |  | 53.27 ± 8.02 | 58.15 ± 8.85 |  | 2.02 ± 0.49 | 2.31 ± 0.60 |  |  |  |  | 55.32 ± 7.02 | 59.28 ± 7.13 |  | 332.56 ± 27.81 | 347.05 ± 30.51 |  |
|  |  | Wang (2022)/China, Hangzhou | Baduanjin + PR | 1.24 ± 0.32 | 1.69 ± 0.41 |  |  |  |  | 1.99 ± 0.44 | 2.65 ± 0.67 |  |  |  |  | 58.60 ± 9.76 | 68.74 ± 10.12 |  | 261.72 ± 40.25 | 343.67 ± 54.82 |  |
|  |  |  | PR | 1.26 ± 0.33 | 1.42 ± 0.36 |  |  |  |  | 2.01 ± 0.43 | 2.28 ± 0.51 |  |  |  |  | 57.93 ± 9.50 | 62.71 ± 9.94 |  | 262.03 ± 38.44 | 308.15 ± 48.07 |  |
|  |  | Yu (2019)/China, Wuxi | Baduanjin + health education |  |  |  |  |  |  |  |  |  |  |  |  |  |  |  | 321.13 ± 42.31 | 415.32 ± 55.32 |  |
|  |  |  | Usual care |  |  |  |  |  |  |  |  |  |  |  |  |  |  |  | 320.86 ± 40.97 | 378.65 ± 50.56 |  |
|  |  | Cao (2016)/China, Nanjing | Baduanjin + health education | 1.35 ± 0.35 | 1.43 ± 0.42 |  | 55.14 ± 10.47 | 57.27 ± 9.45 |  |  |  |  |  |  |  | 64.65 ± 7.04 | 64.64 ± 7.48 |  |  |  |  |
|  |  |  | Walking exercise + health education | 1.34 ± 0.33 | 1.23 ± 0.28 |  | 56.32 ± 7.67 | 53.37 ± 7.56 |  |  |  |  |  |  |  | 63.19 ± 7.25 | 61.64 ± 7.49 |  |  |  |  |
| Moxibustion | Moxibustion - unspecified | Wang (2016)/China, Nanning | Moxibustion |  |  |  | 42.9 ± 15.0 | 46.2 ± 16.6 |  |  |  |  |  |  |  | 51.52 ± 9.55 | 57.43 ± 9.65 |  |  |  |  |
|  |  |  | Usual care |  |  |  | 43.9 ± 10.8 | 48.5 ± 12.3 |  |  |  |  |  |  |  | 52.02 ± 7.43 | 55.73 ± 8.77 |  |  |  |  |
|  |  | Li (2011)/China, Guangzhou | Moxibustion |  |  |  | 52.39 ± 6.45 | 56.42 ± 6.93 |  |  |  |  |  |  |  | 55.86 ± 3.23 | 58.23 ± 3.02 |  | 332.44 ± 84.95 | 369.84 ± 82.16 |  |
|  |  |  | Usual care |  |  |  | 51.70 ± 7.02 | 52.29 ± 6.85 |  |  |  |  |  |  |  | 54.77 ± 2.93 | 55.43 ± 2.87 |  | 321.68 ± 92.76 | 331.12 ± 90.43 |  |
|  | Ginger moxibustion (隔姜灸) | He (2013)/China, Shanghai | Ginger moxibustion | 1.19 ± 0.52 | 1.42 ± 0.64 |  | 47.74 ± 16.09 | 56.03 ± 18.71 |  | 2.16 ± 0.71 | 2.17 ± 0.86 |  |  |  |  | 55.02 ± 10.27 | 65.12 ± 20.84 |  |  |  |  |
|  |  |  | Usual care | 1.17 ± 0.35 | 1.25 ± 0.43 |  | 46.06 ± 14.73 | 50.81 ± 13.29 |  | 2.15 ± 0.72 | 2.16 ± 0.67 |  |  |  |  | 56.94 ± 9.99 | 57.93 ± 9.41 |  |  |  |  |
|  |  | Cui (2017)/China, Changzhi | Ginger moxibustion: Treatment during 20 min | 1.35 ± 0.17 | 2.01 ± 0.18 |  |  |  |  | 1.70 ± 0.39 | 2.40 ± 0.20 |  |  |  |  |  |  |  |  |  |  |
|  |  |  | Usual care | 1.33 ± 0.18 | 1.55 ± 0.25 |  |  |  |  | 1.61 ±0.38 | 1.93 ± 0.39 |  |  |  |  |  |  |  |  |  |  |
|  |  |  | Ginger moxibustion: Treatment during 40 min | 1.26 ± 0.15 | 1.78 ± 0.23 |  |  |  |  | 1.79 ± 0.35 | 2.09 ± 0.34 |  |  |  |  |  |  |  |  |  |  |
|  | Heat-sensitive point’s moxibustion (热敏灸) | Liang (2018)/China, Foshan | Heat-sensitive point’s moxibustion |  |  |  | 47.81 ± 5.17 | 53.76 ± 5.88 |  |  |  |  |  |  |  | 50.05 ± 5.41 | 55.95 ± 5.89 |  |  |  |  |
|  |  |  | Usual care |  |  |  | 47.69 ± 5.03 | 50.12 ± 5.40 |  |  |  |  |  |  |  | 50.03 ± 5.34 | 52.99 ± 5.69 |  |  |  |  |
|  |  | Chen (2017)/China, Nanchang | (G1) Heat-sensitive point’s moxibustion |  |  |  | 64.37 ± 6.55 | 76.94 ± 4.03 |  |  |  |  |  |  |  | 55.81 ± 5.26 | 69.88 ± 6.05 |  |  |  |  |
|  |  |  | (G1) Usual care |  |  |  | 65.33 ± 4.10 | 70.18 ± 6.72 |  |  |  |  |  |  |  | 57.26 ± 8.31 | 65.47 ± 6.59 |  |  |  |  |
|  |  |  | (G2) Heat-sensitive point’s moxibustion |  |  |  | 45.02 ± 4.84 | 65.75 ± 5.93 |  |  |  |  |  |  |  | 49.63 ± 7.29 | 62.34 ± 4.78 |  |  |  |  |
|  |  |  | (G2) Usual care |  |  |  | 44.91 ± 5.39 | 50.36 ± 6.65 |  |  |  |  |  |  |  | 49.10 ± 6.82 | 56.57 ± 7.48 |  |  |  |  |
|  |  | Fan (2021)/China, Haikou | Heat-sensitive point’s moxibustion |  |  |  | 58.56 ± 3.22 | 78.32 ± 3.56 |  |  |  |  |  |  |  | 58.21 ± 3.13 | 76.38 ± 3.26 |  | 310.16 ± 52.26 | 372.25 ± 50.32 |  |
|  |  |  | Usual care |  |  |  | 58.76 ± 3.32 | 60.76 ± 3.48 |  |  |  |  |  |  |  | 58.18 ± 3.26 | 68.32 ± 3.26 |  | 311.32 ± 52.32 | 356.31 ± 50.69 |  |
|  |  | Wang (2011)/China, Zhengzhou | Heat-sensitive point’s moxibustion |  |  |  | 56.79 ± 10.68 | 80.83 ± 10.68 |  |  |  |  |  |  |  | 55.87 ± 11.83 | 74.46 ± 11.43 |  |  |  |  |
|  |  |  | Usual care |  |  |  | 55.66 ± 11.79 | 62.05 ± 10.36 |  |  |  |  |  |  |  | 54.83 ± 11.69 | 60.57 ± 12.35 |  |  |  |  |
|  |  | Zhe (2017)/China, Yan’an | Heat-sensitive point’s moxibustion + pursed-lip breathing | (mL/s) 1485.36 ± 46.58 | (mL/s) 1621.25 ± 55.21 |  | 51.36 ± 4.12 | 62.35 ± 4.18 |  |  |  |  |  |  |  | 59.96 ± 3.65 | 68.51 ± 3.12 |  |  |  |  |
|  |  |  | Usual care + pursed-lip breathing | (mL/s) 1498.25 ± 33.37 | (mL/s) 1557.21 ± 35.47 |  | 52.22 ± 3.58 | 58.36 ± 3.95 |  |  |  |  |  |  |  | 58.85 ± 3.41 | 65.23 ± 3.48 |  |  |  |  |
|  |  | Cheng (2011)/China, Jiujiang | Heat-sensitive point’s moxibustion | (mL/s) 1484.20 ± 46.17 | (mL/s) 1574.43 ± 55.80 | (mL/s) 88.33 ± 12.51 | 53.20 ± 4.15 | 62.59 ± 5.18 | 9.02 ± 3.01 |  |  |  |  |  |  | 59.96 ± 3.95 | 68.69 ± 4.95 | 8.73 ± 2.88 |  |  |  |
|  |  |  | Usual care | (mL/s) 1477.63 ± 39.83 | (mL/s) 1546.63 ± 37.26 | (mL/s) 69.00 ± 8.44 | 52.23 ± 3.74 | 58.26 ± 4.37 | 6.19 ± 1.71 |  |  |  |  |  |  | 59.53 ± 3.74 | 65.81 ± 4.32 | 6.21 ± 1.37 |  |  |  |
|  |  |  | Traditional moxibustion | (mL/s) 1496.67 ± 33.26 | (mL/s) 1572.74 ± 34.71 | (mL/s) 76.07 ± 12.65 | 51.03 ± 4.79 | 58.73 ± 4.12 | 7.72 ± 2.93 |  |  |  |  |  |  | 58.81 ± 3.43 | 66.05 ± 3.39 | 7.3 ± 2.71 |  |  |  |
|  | Yi Fei moxibustion (益肺灸) | Zhao (2018)/China, Nanjing | Yi Fei moxibustion | 1.11 ± 0.28 | 1.36 ± 0.14 |  | 54.23 ± 9.46 | 57.76 ± 9.08 |  |  |  |  |  |  |  | 58.04 ± 8.76 | 59.32 ± 10.23 |  |  |  |  |
|  |  |  | Usual care | 1.13 ± 0.14 | 1.15 ± 0.12 |  | 51.28 ± 8.86 | 50.93 ± 9.35 |  |  |  |  |  |  |  | 53.88 ± 9.77 | 53.76 ± 9.93 |  |  |  |  |
|  |  | Li (2015)/China, Zhengzhou | Yi Fei moxibustion | 1.44 ± 0.77 | 1.44 ± 0.77 |  | 52.72 ± 18.88 | 52.43 ± 18.84 |  | 2.42 ± 0.84 | 2.44 ± 0.87 |  |  |  |  |  |  |  | 431.79 ± 115.05 | 466.97 ± 111.76 |  |
|  |  |  | Usual care | 1.16 ± 0.51 | 1.15 ± 0.50 |  | 46.62 ± 16.90 | 46.27 ± 16.93 |  | 2.12 ± 0.62 | 2.07 ± 0.58 |  |  |  |  |  |  |  | 393.63 ± 107.86 | 402.20 ± 118.16 |  |
|  |  | Huang (2021)/China, Zhengzhou | Yi Fei moxibustion | 1.44 ± 0.29 | 1.90 ± 0.33 |  |  |  |  | 2.06 ± 0.41 | 2.65 ± 0.68 |  |  |  |  | 55.00 ± 7.35 | 70.87 ± 7.32 |  | 240.56 ± 28.75 | 410.86 ± 28.54 |  |
|  |  |  | PR | 1.42 ± 0.26 | 1.71 ± 0.36 |  |  |  |  | 2.11 ± 0.44 | 2.33 ± 0.63 |  |  |  |  | 55.09 ± 7.42 | 63.33 ± 7.56 |  | 240.87 ± 28.63 | 365.56 ± 28.77 |  |
|  |  | Han (2017)/China, Yue Pu Hu Xian | Yi Fei moxibustion |  |  |  | 54.44 ± 4.73 | 56.77 ± 3.93 |  |  |  |  |  |  |  | 55.29 ± 5.03 | 59.81 ± 4.62 |  |  |  |  |
|  |  |  | Usual care |  |  |  | 54.21 ± 4.57 | 55.43 ± 2.95 |  |  |  |  |  |  |  | 56.23 ± 3.74 | 57.01 ± 4.28 |  |  |  |  |
|  |  | Qian (2014)/China, Zhengzhou | Yi Fei moxibustion | 1.26 ± 0.26 | 1.81 ± 0.59 |  | 48.83 ± 10.42 | 58.79 ± 12.04 |  | 2.35 ± 0.36 | 2.81 ± 0.59 |  |  |  |  |  |  |  | 346.77 ± 43.87 | 430.87 ± 48.99 |  |
|  |  |  | Usual care | 1.27 ± 0.33 | 1.44 ± 0.35 |  | 51.37 ± 10.98 | 54.18 ± 12.07 |  | 2.37 ± 0.47 | 2.47 ± 0.46 |  |  |  |  |  |  |  | 356.33 ± 78.91 | 397.77 ± 50.01 |  |
|  |  | Yang (2016)/China, Puyang | Yi Fei Moxibustion |  |  |  | 56.28 ± 10.72 | 80.24 ± 10.29 |  |  |  |  |  |  |  | 54.63 ± 10.84 | 73.68 ± 11.23 |  |  |  |  |
|  |  |  | Usual care |  |  |  | 55.19 ± 10.81 | 64.53 ± 11.63 |  |  |  |  |  |  |  | 53.88 ± 10.64 | 60.52 ± 11.38 |  |  |  |  |
|  |  | Cui (2015)/China, Zhengzhou | Yi Fei moxibustion |  |  |  | 58.31 ± 4.72 | 62.35 ± 5.37 |  |  |  |  |  |  |  | 56.23 ± 4.35 | 59.46 ± 3.51 |  |  |  |  |
|  |  |  | Usual care |  |  |  | 56.39 ± 4.74 | 58.83 ± 4.02 |  |  |  |  |  |  |  | 56.68 ± 4.27 | 58.48 ± 3.48 |  |  |  |  |
|  |  | Zhou (2011)/China, Zhengzhou | Yi Fei moxibustion | 1.61 ± 0.06 | 1.89 ± 0.19 |  | 61.03 ± 6.36 | 66.82 ± 7.49 |  | 2.41 ± 0.18 | 2.83 ± 0.40 |  |  |  |  |  |  |  |  |  |  |
|  |  |  | Usual care | 1.65 ± 0.11 | 1.66 ± 0.88 |  | 60.48 ± 5.65 | 61.91 ± 5.01 |  | 2.41 ± 0.13 | 2.45 ± 0.21 |  |  |  |  |  |  |  |  |  |  |
|  |  | Zhang (2012)/China, Zhengzhou | Yi Fei moxibustion | 0.67 ± 0.43 | 0.81 ± 0.44 |  | 50.13 ± 14.09 | 55.11 ± 18.10 |  |  |  |  |  |  |  |  |  |  |  |  |  |
|  |  |  | Usual care | 0.67 ± 0.35 | 0.79 ± 0.37 |  | 50.88 ± 16.72 | 52.77 ± 16.55 |  |  |  |  |  |  |  |  |  |  |  |  |  |
|  | Fire-dragon moxibustion (火龙灸) | Xu (2022)/China, Guangzhou | Fire-dragon moxibustion |  |  |  | 50.96 ± 12.91 | 56.25 ± 15.52 |  |  |  |  | 68.31 ± 18.18 | 76.19 ± 17.40 |  | 60.65 ± 9.34 | 62.04 ± 9.83 |  |  |  |  |
|  |  |  | Usual care |  |  |  | 47.07 ± 11.32 | 47.12 ± 13.05 |  |  |  |  | 68.53 ± 14.61 | 69.06 ± 16.15 |  | 56.89 ± 9.31 | 57.51 ± 11.30 |  |  |  |  |
| Chuna |  | Rocha (2015)/Brazil | Manual diaphragm release technique |  |  |  |  |  |  |  |  |  |  |  |  |  |  |  | 446.61 ± 81.20 | 461.73 ± 82.47 |  |
|  |  |  | Sham |  |  |  |  |  |  |  |  |  |  |  |  |  |  |  | 421.56 ± 63.01 | 415.11 ± 61.74 |  |
|  |  | Noll (2008)/United States | OMT | 1.22 ± 0.65 | 1.18 ± 0.62 |  | 45 ± 23 | 44 ± 22 |  | 2.50 ± 0.94 | 2.36 ± 0.93 |  | 73 ± 23 | 69 ± 23 |  | 47.72 ± 13.23 | 48.89 ± 12.88 |  |  |  |  |
|  |  |  | Sham | 1.26 ± 0.57 | 1.28 ± 0.63 |  | 46 ± 20 | 46 ± 22 |  | 2.71 ± 0.87 | 2.66 ± 0.92 |  | 77 ± 20 | 75 ± 21 |  | 46.41 ± 13.05 | 45.88 ± 16.66 |  |  |  |  |
|  |  | Maskey-Warzechowska (2019)/Poland | OMT | median (Q1, Q3) 1.1 (0.8-1.4) | median (Q1, Q3) 1.0 (0.7-1.3) |  | median (Q1, Q3) 43.4 (34.5-46.7) | median (Q1, Q3) 38.9 (33.6-45.8) |  | median (Q1, Q3) 2.9 (2.4-3.7) | median (Q1, Q3) 3.2 (2.2-3.7) |  | median (Q1, Q3) 87.7 (76.8-98.2) | median (Q1, Q3) 87.5 (73.2-97.7) |  | median (Q1, Q3) 33.3 (29.4-43.1) | median (Q1, Q3) 33.2 (30.0-43.3) |  |  |  |  |
|  |  |  | Sham | median (Q1, Q3) 1.0 (0.7-1.3) | median (Q1, Q3) 1.0 (0.8-1.3) |  | median (Q1, Q3) 36.8 (33.1-46.4) | median (Q1, Q3) 398.0 (33.6-47.4) |  | median (Q1, Q3) 3.0 (2.4-3.6) | median (Q1, Q3) 2.9 (2.3-3.7) |  | median (Q1, Q3) 85.9 (72.6-93.1) | median (Q1, Q3) 85.5 (73.9-94.4) |  | median (Q1, Q3) 32.4 (29.2-43.1) | median (Q1, Q3) 31.4 (28.5-43.7) |  |  |  |  |
|  |  | Kurzaj (2013)/Poland | 6 specialized physiotherapies + basic physiotheraphy | 1.1 ± 0.19 | 1.4 ± 0.26 |  | 45.2 ± 19.04 | 59.2 ± 14.18 |  |  |  |  |  |  |  |  |  |  | 241.0 ± 78.8 | 318.7 ± 73.6 |  |
|  |  |  | basic physiotherapy | 1.2 ± 0.8 | 1.4 ± 0.7 |  | 40.2 ± 13.0 | 42.7 ± 11.9 |  |  |  |  |  |  |  |  |  |  | 229.0 ± 87.1 | 262.5 ± 89.9 |  |
|  |  | Buran Cirak (2022)/Turkey | Manual therapy + inspiratory muscle training |  |  |  | 34.6 ± 7.33 | 40.1 ± 5.26 | 4.18 (2.15 to 8.74) |  |  |  | 50.4 ± 10.6 | 54.7 ± 12.0 | 4.06 (1.59 to 10.1) | 56.2 ± 9.15 | 59.3 ± 8.63 | 3.14 (1.43 to 7.75) | 395.1 ± 106.8 | 467.2 ± 112.4 | 66.1 (34.6 to 98.7) |
|  |  |  | Inspiratory muscle training |  |  |  | 36.4 ± 8.12 | 37.9 ± 10.2 | 0.64 (-3.25 to 6.29) |  |  |  | 51.1 ± 11.6 | 52.9 ± 10.7 | 0.72 (-3.98 to 7.60) | 58.2 ± 12.4 | 52.98 ± 10.6 | 0.93 (-4.36 to 7.60) | 386.7 ± 110.4 | 430.2 ± 114.5 | 42.5 (10.7 to -81.5) |
|  |  | Chen (2006)/China, Shanghai | Chuna | 1.248 ± 0.743 | 1.419 ± 0.953 |  | 47.63 ± 10.69 | 54.57 ± 11.25 |  | 2.311 ± 0.875 | 2.628 ± 0.921 |  |  |  |  |  |  |  | 330.51 ± 67.21 | 389.73 ± 72.15 |  |
|  |  |  | Usual care | 1.269 ± 0.881 | 1.333 ± 0.798 |  | 48.25 ± 11.71 | 50.60 ± 9.62 |  | 2.266 ± 0.956 | 2.362 ± 0.759 |  |  |  |  |  |  |  | 328.79 ± 71.13 | 346.65 ± 69.23 |  |
| Acupuncture | Acupuncture (针刺) | Suzuki (2008)/Japan | Acupuncture | 1.25 ± 0.6 | 1.34 ± 0.6 | 0.10 ± 0.2 | 50.2 ± 23.8 | 54.1 ± 24.8 | 3.9 ± 6.3 | 2.42 ± 1.0 | 2.72 ± 1.0 | 0.29 ± 0.3 |  |  |  | 51.4 ± 15.4 | 48.6 ± 15.2 | -2.8 ± 5.3 | 380.2 ± 132.5 | 425.6 ± 136.4 | 45.4 ± 17.2 |
|  |  |  | Usual care | 1.10 ± 0.4 | 1.08 ± 0.4 | -0.02 ± 0.1 | 45.5 ± 15.4 | 44.7 ± 14.6 | -0.73 ± 3.6 | 2.27 ± 0.7 | 2.19 ± 0.7 | -0.08 ± 0.2 |  |  |  | 49.2 ± 11.8 | 50.4 ± 14.4 | 1.3 ± 6.3 | 417.7 ± 107.1 | 390.4 ± 110.1 | -26.8 ± 62.2 |
|  |  | Liu (2015)/China, Shanghai | Acupuncture |  |  |  | 35.71 ± 7.28 | 45.36 ± 7.13 | 10.12 ± 6.58 |  |  |  |  |  |  | 53.31 ± 7.32 | 60.48 ± 6.42 | 7.35 ± 7.01 | 321.67 ± 30.27 | 375.15 ± 50.36 | 53.10 ± 34.27 |
|  |  |  | Usual care |  |  |  | 36.42 ± 6.42 | 42.19 ± 8.34 | 7.01 ± 2.25 |  |  |  |  |  |  | 55.26 ± 6.68 | 58.98 ± 5.64 | 4.02 ± 5.87 | 335.63 ± 40.51 | 380.33 ± 28.85 | 44.70 ± 30.81 |
|  |  | Deering (2011)/Ireland | Acupuncture + PR |  |  |  | 48.8 ± 22.7 | 54.2 ± 23.4 | 1.1 ± 7.2 |  |  |  | 80.7 ± 24.2 | 82.2 ± 24.5 | 2.0 ± 15.7 |  |  |  |  |  |  |
|  |  |  | PR |  |  |  | 48.5 ± 16.1 | 48.1 ± 16.8 | -1.2 ± 7.9 |  |  |  | 77.0 ± 19 | 78.4 ± 20.7 | -1.4 ± 16.3 |  |  |  |  |  |  |
|  |  |  | Usual care |  |  |  | 45.8 ± 18.3 | 44.9 ± 20.1 | 0.8 ± 10.5 |  |  |  | 70.6 ± 19.3 | 67.0 ± 18.5 | -2.5 ± 12.5 |  |  |  |  |  |  |
|  |  | Suzuki (2012)/Japan | Acupuncture | 1.0 ± 0.3 | 1.1 ± 0.3 | 0.07 ± 0.3 | 46.0 ± 16.6 | 49.2 ± 19.5 | 3.17 ± 10.7 | 2.8 ± 0.5 | 2.9 ± 0.5 | 0.14 ± 0.2 |  |  |  |  |  |  |  |  |  |
|  |  |  | Sham | 1.1 ± 0.3 | 1.1 ± 0.3 | -0.04 ± 0.2 | 47.9 ± 16.5 | 46.9 ± 17.7 | -1.00 ± 4.8 | 3.0 ± 0.7 | 2.9 ± 0.6 | -0.10 ± 0.2 |  |  |  |  |  |  |  |  |  |
|  |  | Li (2019)/China, Chengdu | Acupuncture | 1.47 ± 0.44 | 1.61 ± 0.45 | 0.29 ± 0.52 | 53.67 ± 9.03 | 57.74 ± 10.19 | 3.9 ± 6.3 | 2.47 ± 0.57 | 2.73 ± 0.70 | 0.38 ± 0.11 |  |  |  |  |  |  | 303.25 ± 44.73 | 352.41 ± 72.91 | 49.26 ± 21.15 |
|  |  |  | Usual care | 1.42 ± 0.38 | 1.59 ± 0.13 | 0.10 ± 0.47 | 52.88 ± 10.01 | 55.79 ± 10.68 | 1.94 ± 0.06 | 2.50 ± 0.49 | 2.61 ± 0.62 | 0.09 ± 0.13 |  |  |  |  |  |  | 310.56 ± 39.74 | 331.15 ± 75.51 | 18.42 ± 12.01 |
|  |  | Feng (2016)/China, Fuyang & Harbin | Acupuncture |  |  |  | 47.3 ± 17.1 |  | 3.22(-6.7, 12.8) | 2.9 ± 0.7 |  | 0.15(-0.14, 0.389) |  |  |  |  |  |  | 417.3 ± 122.5 |  | 67.4(31.9, 47.3) |
|  |  |  | Sham |  |  |  | 43.9 ± 16.8 |  | -0.9(-3.5, 3.1) | 2.7 ± 0.7 |  | -0.11(-0.27, 0.07) |  |  |  |  |  |  | 394.8 ± 131.6 |  | -13.8(-45.2, -5.7) |
|  |  | Zhong (2018)/China, Maoming | Acupuncture |  |  |  | 61.93 ± 5.28 | 50.58 ± 2.92 |  |  |  |  |  |  |  | 40.37 ± 3.00 | 50.33 ± 6.12 |  |  |  |  |
|  |  |  | Usual care |  |  |  | 53.49 ± 5.47 | 48.34 ± 3.33 |  |  |  |  |  |  |  | 38.47 ± 2.36 | 42.32 ± 0.52 |  |  |  |  |
|  |  | Deng (2016)/China, Guangzhou | Acupuncture |  |  |  | 52.4 ± 2.9 | 59.8 ± 3.5 |  |  |  |  |  |  |  | 51.3 ± 3.5 | 59.4 ± 3.8 |  | 369.3 ± 43.5 | 418.7 ± 52.5 |  |
|  |  |  | Usual care |  |  |  | 51.5 ± 2.7 | 56.2 ± 3.3 |  |  |  |  |  |  |  | 52.0 ± 3.5 | 53.3 ± 3.6 |  | 370.3 ± 44.1 | 390.4 ± 54.3 |  |
|  |  | Yang (2018)/China, Wuhan | Acupuncture | 1.55 ± 0.40 | 1.86 ± 0.42 |  | 45.94 ± 6.03 | 60.11 ± 5.45 |  |  |  |  |  |  |  | 51.28 ± 6.32 | 58.77 ± 5.24 |  |  |  |  |
|  |  |  | Usual care | 1.56 ± 0.34 | 1.97 ± 0.55 |  | 44.73 ± 5.83 | 60.21 ± 6.34 |  |  |  |  |  |  |  | 52.38 ± 6.34 | 60.43 ± 6.22 |  |  |  |  |
|  |  | Jiao (2020)/China, Zhengzhou | Acupuncture + health education |  |  |  |  |  |  |  |  |  |  |  |  |  |  |  | 350.65 ± 71.23 | 450.46 ± 63.87 |  |
|  |  |  | Usual care |  |  |  |  |  |  |  |  |  |  |  |  |  |  |  | 353.72 ± 68.95 | 395.27 ± 70.36 |  |
|  |  | Tang (2017)/China, Fuzhou | Acupuncture |  |  |  |  |  |  |  |  |  |  |  |  |  |  |  | 308.67 ± 24.38 | 427.80 ± 38.08 |  |
|  |  |  | Usual care |  |  |  |  |  |  |  |  |  |  |  |  |  |  |  | 304.76 ± 28.05 | 338.83 ± 17.71 |  |
|  |  | Li (2020)/China, Shenyang | Acupuncture | 1.48 ± 0.24 | 2.75 ± 0.46 |  |  |  |  | 1.47 ± 0.20 | 2.89 ± 0.64 |  |  |  |  |  |  |  | 331.55 ± 86.08 | 433.69 ± 89.25 |  |
|  |  |  | Usual care | 1.45 ± 0.26 | 1.97 ± 0.48 |  |  |  |  | 1.49 ± 0.22 | 2.21 ± 0.51 |  |  |  |  |  |  |  | 330.92 ± 82.15 | 381.57 ± 87.48 |  |
|  |  | Wang (2021)/China, Chongqing | Acupuncture | 1.38 ± 0.19 | 1.87 ± 0.38 |  |  |  |  | 2.50 ± 0.28 | 2.96 ± 0.54 |  |  |  |  | 0.55 ± 0.10 | 0.61 ± 0.12 |  |  |  |  |
|  |  |  | Usual care | 1.47 ± 0.25 | 1.68 ± 0.29 |  |  |  |  | 2.39 ± 0.36 | 2.74 ± 0.41 |  |  |  |  | 0.59 ± 0.21 | 0.63 ± 0.07 |  |  |  |  |
|  | Acu-TENS | Ngai (2010)/China, Hong Kong | Acu-TENS | 0.79 ± 0.10 | 0.86 ± 0.13 | 8.2 ± 2.4 |  |  |  | 1.89 ± 0.19 | 1.94 ± 0.20 | 3.3 ± 3.9 |  |  |  |  |  |  | 305.8 ± 32.5 | 329.5 ± 34.7 | 8.5 ± 4.4 |
|  |  |  | Placebo-TENS | 0.74 ± 0.08 | 0.75 ± 0.10 | 1.1 ± 2.6 |  |  |  | 1.74 ± 0.31 | 1.70 ± 0.27 | 1.9 ± 7.5 |  |  |  |  |  |  | 325.7 ± 19.2 | 333.8 ± 15.3 | 3.1 ± 1.8 |
|  |  |  | Sham-TENS | 0.79 ± 0.10 | 0.73 ± 0.08 | -5.4 ± 3.5 |  |  |  | 1.61 ± 0.15 | 1.64 ± 0.12 | 3.0 ± 3.7 |  |  |  |  |  |  | 346.7 ± 22.5 | 339.0 ± 25.8 | -2.9 ± 2.4 |
|  |  | Liu (2015)/China, Chengdu | Acu-TENS |  |  |  | 47.1 ± 19.2 | 53.6 ± 19.5 |  |  |  |  | 56.3 ± 19.5 | 60.3 ± 13.9 |  |  |  |  | 383.8 ± 98.4 | 418.3 ± 90.6 |  |
|  |  |  | Placebo-TENS |  |  |  | 38.4 ± 18.7 | 39.0 ± 16.8 |  |  |  |  | 50.5 ± 16.1 | 49.4 ± 13.8 |  |  |  |  | 395.2 ± 76.1 | 418.7 ± 57.1 |  |
|  |  | Lau (2008)/China, Hong Kong | Acu-TENS | 1.24 ± 0.46 | 1.37 ± 0.47 | 0.13 ± 0.09 |  |  |  | 1.70 ± 0.44 | 1.77 ± 0.44 | 0.07 ± 0.09 |  |  |  |  |  |  |  |  |  |
|  |  |  | Placebo-TENS | 1.39 ± 0.43 | 1.41 ± 0.43 | 0.01 ± 0.03 |  |  |  | 1.75 ± 0.53 | 1.77 ± 0.52 | 0.02 ± 0.09 |  |  |  |  |  |  |  |  |  |
|  |  | Jones (2011)/China, Sichuan province | Acu-TENS | 0.57 ± 0.04 | 0.66 ± 0.04 |  |  |  |  | 1.09 ± 0.09 | 1.09 ± 0.06 |  |  |  |  |  |  |  |  |  |  |
|  |  |  | Placebo-TENS | 0.66 ± 0.03 | 0.62 ± 0.03 |  |  |  |  | 1.24 ± 0.06 | 1.17 ± 0.05 |  |  |  |  |  |  |  |  |  |  |
|  | Warm acupuncture (温针) | Yang (2016)/China, Xiamen | Warm acupuncture |  |  |  | 68.77 ± 13.83 | 69.63 ± 13.54 |  |  |  |  |  |  |  | 59.23 ± 6.19 | 60.30 ± 5.95 |  |  |  |  |
|  |  |  | Usual care |  |  |  | 69.52 ± 13.30 | 70.42 ± 13.16 |  |  |  |  |  |  |  | 59.13 ± 7.92 | 60.19± 7.50 |  |  |  |  |
|  |  | Li (2015)/China, Xiamen | Warm acupuncture |  |  |  | 66.28 ± 6.86 | 67.00 ± 5.94 |  |  |  |  |  |  |  | 61.56 ± 5.64 | 63.34 ± 4.49 |  |  |  |  |
|  |  |  | Usual care |  |  |  | 65.16 ± 6.16 | 65.90 ± 5.76 |  |  |  |  |  |  |  | 60.30 ± 5.82 | 61.24 ± 5.93 |  |  |  |  |
|  |  | Shi (2021)/China, Shanghai | Warm acupuncture | 1.59 ± 0.36 | 1.98 ± 0.43 |  | 58.02 ± 4.86 | 64.28 ± 5.04 |  |  |  |  |  |  |  | 51.95 ± 5.21 | 59.37 ± 6.25 |  | 275.69 ± 36.85 | 402.69 ± 58.92 |  |
|  |  |  | Usual care | 1.57 ± 0.35 | 1.74 ± 0.37 |  | 58.25 ± 4.52 | 60.13 ± 4.58 |  |  |  |  |  |  |  | 51.60 ± 5.24 | 54.95 ± 6.01 |  | 279.17 ± 38.95 | 376.71 ± 51.84 |  |
|  | Auricular acupressure (耳穴贴压) | Pang (2014)/China, Weifang | Auricular acupressure | 1.56 ± 0.65 | 2.03 ± 0.55 |  | 52.58 ± 5.93 | 53.87 ± 5.49 |  |  |  |  |  |  |  | 51.69 ± 6.08 | 52.95 ± 5.89 |  |  |  |  |
|  |  |  | Usual care | 1.55 ± 0.43 | 1.92 ± 0.40 |  | 52.81 ± 5.56 | 54.10 ± 5.28 |  |  |  |  |  |  |  | 50.89 ± 5.83 | 52.20 ± 5.66 |  |  |  |  |
|  |  | Li (2017)/China, Fuzhou | Auricular acupressure + health education | 1.20 ± 0.19 | 1.25 ± 0.22 |  | 54.74 ± 8.93 | 55.34 ± 16.25 |  | 2.58 ± 0.23 | 2.61 ± 0.73 |  |  |  |  | 60.02 ± 5.24 | 63.12 ± 10.05 |  |  |  |  |
|  |  |  | Usual care | 1.23 ± 0.62 | 1.26 ± 0.35 |  | 53.36 ± 8.56 | 54.31 ± 12.94 |  | 2.49 ± 0.76 | 2.59 ± 0.81 |  |  |  |  | 58.12 ± 13.36 | 58.71 ± 14.40 |  |  |  |  |
|  |  |  | Auricular acupressure of midnight-noon Ebb-flow (子午流注鍼法) | 1.21 ± 0.15 | 1.26 ± 0.46 |  | 53.58 ± 10.12 | 56.37 ± 9.41 |  | 2.50 ± 0.22 | 2.60 ± 0.34 |  |  |  |  | 59.03 ± 3.41 | 60.41 ± 4.39 |  |  |  |  |
|  |  | Jin (2009)/China, Wenzhou | Auricular acupressure | 0.71 ± 0.19 | 0.76 ± 0.29 |  | 30.00 ± 10.8 | 29.40 ± 9.6 |  |  |  |  | 50.60 ± 9.7 | 55.50 ± 16.8 |  | 57.10 ± 8.8 | 59.10 ± 17.2 |  |  |  |  |
|  |  |  | Usual care | 0.68 ± 0.22 | 0.69 ± 0.25 |  | 32.30 ± 12.2 | 29.80 ± 12.5 |  |  |  |  | 50.40 ± 15.5 | 49.90 ± 16.1 |  | 58.70 ± 17.5 | 59.20 ± 13.8 |  |  |  |  |
|  | Electroacupuncture (电针) | He (2021)/China, Guangzhou | Electroacupuncture + bicycle exercise |  |  |  | 45.05 ± 24.47 | 46.25 ± 19.61 |  |  |  |  | 70.69 ± 22.82 | 74.42 ± 21.98 |  | 60.42 ± 18.39 | 61.04 ± 18.02 |  | 447.51 ± 124.10 | 495.77 ± 140.74 |  |
|  |  |  | Placebo needling + bicycle exercise |  |  |  | 43.95 ± 18.24 | 41.85 ± 17.94 |  |  |  |  | 72.97 ± 15.75 | 74.13 ± 17.42 |  | 58.94 ± 19.23 | 55.77 ± 19.48 |  | 412.33 ± 101.84 | 427.22 ± 105.08 |  |
|  |  | Ge (2017)/China, Guangzhou | Electroacupuncture + bicycle exercise |  |  |  | 40.76 ± 16.36 | 45.62 ± 19.29 |  |  |  |  | 68.43 ± 22.13 | 73.64 ± 23.78 |  | 49.70 ± 14.61 | 52.74 ± 13.54 |  | 438.05 ± 126.75 | 496.10 ± 130.06 | 58.05 ± 51.52 |
|  |  |  | Placebo needling + bicycle exercise |  |  |  | 40.53 ± 17.40 | 39.45 ± 16.33 |  |  |  |  | 72.61 ± 14.64 | 71.82 ± 14.44 |  | 46.53 ± 15.00 | 44.84 ± 15.45 |  | 414.65 ± 102.83 | 433.68 ± 106.00 | 19.03 ± 48.18 |
|  | Pressing needle (皮内针) | Chen (2018)/China, Deyang | Pressing needle + diaphragmatic and pursed-lip breathing |  |  |  |  | 67 ± 20 |  |  | 3.2 ± 0.43 |  |  |  |  |  | 60 ± 11 |  |  |  |  |
|  |  |  | Diaphragmatic and pursed-lip breathing |  |  |  |  | 51 ± 15 |  |  | 3.0 ± 0.5 |  |  |  |  |  | 48 ± 14 |  |  |  |  |
|  |  | Peng (2015)/China, Chengdu | Pressing needle + health education |  |  |  | 67.07 ± 16.46 | 65.69 ± 18.03 |  |  |  |  | 82.28 ± 14.55 | 83.24 ± 13.61 |  | 59.45 ± 6.90 | 60.31 ± 6.54 |  |  |  |  |
|  |  |  | Usual care |  |  |  | 66.57 ± 16.30 | 65.40 ± 15.48 |  |  |  |  | 82.27 ± 14.78 | 81.40 ± 14.85 |  | 58.40 ± 7.32 | 58.13 ± 6.08 |  |  |  |  |
|  |  | Wang (2019)/China, Shanghai | Pressing needle |  |  |  |  |  |  |  |  |  |  |  |  |  |  |  | 299.41 ± 23.63 | 355.29 ± 29.24 |  |
|  |  |  | Usual care |  |  |  |  |  |  |  |  |  |  |  |  |  |  |  | 295.13 ± 31.54 | 329.08 ± 23.38 |  |
|  | Acupressure (穴位按揉) | Tuo (2018)/China, Wuzhou | Acupressure |  |  |  | 39.36 ± 8.11 | 46.09 ± 7.36 |  |  |  |  |  |  |  |  |  |  | 247.41 ± 50.25 | 312.30 ± 32.30 |  |
|  |  |  | Usual care |  |  |  | 39.40 ± 7.97 | 38.27 ± 7.34 |  |  |  |  |  |  |  |  |  |  | 247.25 ± 48.76 | 242.61 ± 43.85 |  |
| **Abbreviations:** FEV_1_, forced expiratory volume in 1 second; FVC, forced vital capacity; 6MWD, 6-minute walking distance; MD, mean difference; PR, pulmonary rehabilitation; prd, predicted. | | | | | | | | | | | | | | | | | | | | | |

Table S2. Summary of risk of bias based on the Cochrane risk of bias tool

| **Category** |  | **Author(year)/Country, city** | **Random sequence generation** | **Allocation concealment** | **Blinding of participants and personnel** | **Blinding of the outcome assessment** | **Incomplete outcome data addressed** | **Selective reporting** | **Other bias** |
| --- | --- | --- | --- | --- | --- | --- | --- | --- | --- |
| Qigong | Qigong (气功) | Dong (2021)/China, Jinan | Low risk | Low risk | Unclear risk | Low risk | Low risk | Unclear risk | Unclear risk |
|  | Health qigong integrated for lung health | Li (2019)/China, Shanghai | Low risk | Low risk | High risk | Low risk | Low risk | Unclear risk | Unclear risk |
|  |  | Liu (2012)/China, Shanghai | Low risk | Unclear risk | High risk | Low risk | Unclear risk | Unclear risk | Unclear risk |
|  |  | Liu (2011)/China, Jiangsu | Unclear risk | Unclear risk | High risk | Unclear risk | Unclear risk | Unclear risk | Unclear risk |
|  | Wuqinxi (五禽戏) | Wei (2015)/China, Bozhou | Unclear risk | Unclear risk | Unclear risk | Unclear risk | Low risk | Unclear risk | Unclear risk |
|  |  | Zhao (2015)/China, Zhengzhou | Unclear risk | Unclear risk | Unclear risk | Unclear risk | Unclear risk | Unclear risk | Unclear risk |
|  |  | Yao (2021)/China, Zhangjiajie | Unclear risk | Unclear risk | Unclear risk | Unclear risk | Low risk | Unclear risk | Unclear risk |
|  | Liuzijue (六字诀) | Jiang (2017)/China,Changsha | Low risk | Unclear risk | Unclear risk | Unclear risk | Low risk | Unclear risk | Unclear risk |
|  |  | Hou (2017)/China, Xianyang | Low risk | Unclear risk | Unclear risk | Unclear risk | Low risk | Unclear risk | Unclear risk |
|  |  | Zhang (2009)/China, Fujian | Unclear risk | Unclear risk | Unclear risk | Unclear risk | Low risk | Unclear risk | Unclear risk |
|  |  | Wu (2018)/China, Shanghai(a) | Low risk | Low risk | High risk | Low risk | Low risk | Unclear risk | Unclear risk |
|  |  | Zhu (2011)/China, Nanjing | Unclear risk | Unclear risk | Unclear risk | Unclear risk | Low risk | Unclear risk | Unclear risk |
|  |  | Xiao (2015)/China, Beijing) | Unclear risk | Unclear risk | High risk | Low risk | Low risk | Unclear risk | Unclear risk |
|  |  | Wu (2018)/China, Shanghai(b) | Low risk | Low risk | High risk | Low risk | Low risk | Unclear risk | Unclear risk |
|  |  | Li (2018)/China, Shanghai | Low risk | Unclear risk | High risk | Low risk | Low risk | Unclear risk | Unclear risk |
|  |  | Chen (2008)/China, Fujian | Unclear risk | Unclear risk | Unclear risk | Unclear risk | Unclear risk | Unclear risk | Unclear risk |
|  |  | Yan (2020)/China, Taian | Low risk | Unclear risk | Unclear risk | Unclear risk | Low risk | Unclear risk | Unclear risk |
|  |  | Deng (2020)/China, Fuzhou | Low risk | Low risk | Unclear risk | Unclear risk | Low risk | Unclear risk | Unclear risk |
|  |  | Deng (2018)/China, Fuzhou | Low risk | Unclear risk | Unclear risk | Unclear risk | Low risk | Unclear risk | Unclear risk |
|  |  | Deng (2009)/China, Fuzhou | Low risk | Unclear risk | Unclear risk | Unclear risk | Low risk | Unclear risk | Unclear risk |
|  |  | He (2019)/China, Beijing | Low risk | Unclear risk | Unclear risk | Unclear risk | Low risk | Unclear risk | Unclear risk |
|  |  | Lan (2016)/China, Luzhou | Unclear risk | Unclear risk | Unclear risk | Unclear risk | Unclear risk | Unclear risk | Unclear risk |
|  |  | Zhang (2021)/China, Tianjin | Low risk | Low risk | High risk | High risk | Low risk | Unclear risk | Unclear risk |
|  |  | Lu (2021)/China, Fujian province | Low risk | Unclear risk | Unclear risk | Unclear risk | Low risk | Unclear risk | Unclear risk |
|  |  | Liu (2017)/China, Shanghai | Low risk | Low risk | Unclear risk | Unclear risk | Low risk | Unclear risk | Unclear risk |
|  |  | Hu (2021)/China, Shanghai | Unclear risk | Unclear risk | Unclear risk | Unclear risk | Low risk | Unclear risk | Unclear risk |
|  |  | Shen (2017)/China, Shanghai | Low risk | Low risk | Unclear risk | Low risk | Low risk | Unclear risk | Unclear risk |
|  |  | Ji (2019)/China, Shanghai | Unclear risk | Unclear risk | Unclear risk | Unclear risk | Low risk | Unclear risk | Unclear risk |
|  |  | Qu (2019)/China, Taizhou | Low risk | Unclear risk | Unclear risk | Unclear risk | Low risk | Unclear risk | Unclear risk |
|  |  | Li (2011)/China, Fuzhou | Low risk | Unclear risk | Unclear risk | Unclear risk | Unclear risk | Unclear risk | Unclear risk |
|  |  | Li (2018)/China, Beijing | Low risk | Unclear risk | Unclear risk | Unclear risk | Low risk | Unclear risk | Unclear risk |
|  |  | Yan (2021)/China, Lanzhou | Unclear risk | Unclear risk | Unclear risk | Unclear risk | Low risk | Unclear risk | Unclear risk |
|  |  | Wang (2013)/China, Shanghai | Low risk | Unclear risk | Unclear risk | Unclear risk | Low risk | Unclear risk | Unclear risk |
|  |  | Quan (2021)/China, Tianjin | Low risk | Unclear risk | Unclear risk | Unclear risk | Low risk | Unclear risk | Unclear risk |
|  |  | Liu (2021)/China, Shanghai | Low risk | Low risk | Unclear risk | Low risk | Low risk | Unclear risk | Unclear risk |
|  | Yijinging (易筋经) | Zhang (2016)/China, Jiangsu province | Unclear risk | Unclear risk | Unclear risk | Unclear risk | Low risk | Unclear risk | Unclear risk |
|  |  | Gao (2015)/China, Suzhou | Low risk | Unclear risk | Unclear risk | Unclear risk | Low risk | Unclear risk | Unclear risk |
|  | Tai chi (太极) | Zhu (2018)/China, Changsha | Low risk | Unclear risk | Unclear risk | Unclear risk | Low risk | Unclear risk | Unclear risk |
|  |  | Zhang (2014)/China, Jinan | Low risk | Unclear risk | Unclear risk | Unclear risk | Low risk | Unclear risk | Unclear risk |
|  |  | Zhang (2012)/China, Beijing | Low risk | Unclear risk | Unclear risk | Unclear risk | Low risk | Unclear risk | Unclear risk |
|  |  | Yeh (2020)/USA, Boston | Low risk | Unclear risk | Unclear risk | Low risk | Low risk | Unclear risk | Unclear risk |
|  |  | Wang (2014)/China, Yingde & Zhengzhou | Unclear risk | Unclear risk | Unclear risk | Unclear risk | Low risk | Unclear risk | Unclear risk |
|  |  | Wang (2019)/China, Zunyi | Low risk | Unclear risk | High risk | Low risk | Low risk | Unclear risk | Unclear risk |
|  |  | Polkey (2018)/China, Meizhou | Unclear risk | Unclear risk | Unclear risk | Unclear risk | Low risk | Unclear risk | Unclear risk |
|  |  | Niu (2014)/China, Changsha | Low risk | Low risk | High risk | Low risk | Low risk | Unclear risk | Unclear risk |
|  |  | Ng (2014)/China, Hong Kong | Low risk | Low risk | Unclear risk | Unclear risk | Low risk | Unclear risk | Unclear risk |
|  |  | Yeh (2010)/USA, Boston | Low risk | Unclear risk | Unclear risk | Low risk | Low risk | Unclear risk | Unclear risk |
|  |  | Moy (2021)/USA, Boston | Low risk | Low risk | Unclear risk | Low risk | Low risk | Unclear risk | Unclear risk |
|  |  | Kantatong (2020)/Thailand, Chiang Mai | Low risk | Low risk | Unclear risk | Low risk | Low risk | Unclear risk | Unclear risk |
|  |  | Du (2013)/China, Zhangjiakou | Unclear risk | Unclear risk | Unclear risk | Unclear risk | Unclear risk | Unclear risk | Unclear risk |
|  |  | Chan (2013)/China, Hong Kong | Low risk | Unclear risk | Unclear risk | Unclear risk | Low risk | Unclear risk | Unclear risk |
|  |  | Zhang (2019)/China, Chongqing | Unclear risk | Unclear risk | Unclear risk | Unclear risk | Low risk | Unclear risk | Unclear risk |
|  |  | Cui (2016)/China, Zhangjiakou | Low risk | Unclear risk | Unclear risk | Unclear risk | Unclear risk | Unclear risk | Unclear risk |
|  |  | Li (2019)/China, Fenyang | Unclear risk | Unclear risk | Unclear risk | Unclear risk | Unclear risk | Unclear risk | Unclear risk |
|  |  | Li (2016)/China, Yantai | Low risk | Unclear risk | Unclear risk | Unclear risk | Low risk | Unclear risk | Unclear risk |
|  |  | Li (2012)/China, Jinan | Unclear risk | Unclear risk | Unclear risk | Unclear risk | Low risk | Unclear risk | Unclear risk |
|  |  | Pan (2018)/China, Chengdu | Low risk | Unclear risk | Unclear risk | Unclear risk | Low risk | Unclear risk | Unclear risk |
|  |  | Ren (2017)/China, Beijing | Low risk | Unclear risk | Unclear risk | Unclear risk | Low risk | Unclear risk | Unclear risk |
|  |  | He (2019)/China, Jinan | Low risk | Unclear risk | Unclear risk | Unclear risk | High risk | Unclear risk | Unclear risk |
|  | Baduanjin (八段锦) | Lu (2015)/China, Beijing | High risk | Unclear risk | Unclear risk | Unclear risk | Low risk | Unclear risk | Unclear risk |
|  |  | Feng (2009)/China, Guangzhou | Unclear risk | Unclear risk | Unclear risk | Unclear risk | Low risk | Unclear risk | Unclear risk |
|  |  | Huang (2017)/China, Nanjing | Low risk | Unclear risk | Unclear risk | Unclear risk | Low risk | Unclear risk | Unclear risk |
|  |  | Yin (2013)/China, Guangzhou | Low risk | Low risk | Unclear risk | Unclear risk | Unclear risk | Unclear risk | Unclear risk |
|  |  | Chen (2015)/China, Fuzhou | Unclear risk | Unclear risk | Unclear risk | Unclear risk | Low risk | Unclear risk | Unclear risk |
|  |  | Zhu (2014)/China, Changsha | Low risk | Unclear risk | Unclear risk | Unclear risk | High risk | Unclear risk | Unclear risk |
|  |  | Ng (2011)/China, Hong Kong | Low risk | Low risk | High risk | Low risk | High risk | Unclear risk | Unclear risk |
|  |  | Xu (2010)/China, Yunyang | Unclear risk | Unclear risk | Unclear risk | Unclear risk | Unclear risk | Unclear risk | Unclear risk |
|  |  | Huang (2016)/China, Dongguan | Unclear risk | Unclear risk | Unclear risk | Unclear risk | Unclear risk | Unclear risk | Unclear risk |
|  |  | Chen (2017)/China, Shenyang | Low risk | Unclear risk | Unclear risk | Unclear risk | Unclear risk | Unclear risk | Unclear risk |
|  |  | Chen (2015)/China, Chengdu | Low risk | Unclear risk | Unclear risk | Unclear risk | Low risk | Unclear risk | Unclear risk |
|  |  | Zheng (2019)/China, Guangzhou | Low risk | Low risk | Unclear risk | Unclear risk | Unclear risk | Unclear risk | Unclear risk |
|  |  | Deng (2014)/China, Fuzhou | Low risk | Low risk | Unclear risk | Low risk | Low risk | Unclear risk | Unclear risk |
|  |  | Deng (2020)/China, Guangzhou | Low risk | Unclear risk | Unclear risk | Unclear risk | Low risk | Unclear risk | Unclear risk |
|  |  | Zhang (2017)/China, Changchun | Low risk | Unclear risk | Unclear risk | Unclear risk | Low risk | Unclear risk | Unclear risk |
|  |  | Sun (2014)/China, Changchun | Low risk | Unclear risk | Unclear risk | Unclear risk | Low risk | Unclear risk | Unclear risk |
|  |  | Liu (2013)/China, Chengdu | Low risk | Unclear risk | Unclear risk | Unclear risk | Low risk | Unclear risk | Unclear risk |
|  |  | Ye (2016)/China, Changsha | Low risk | Unclear risk | Unclear risk | Unclear risk | Low risk | Unclear risk | Unclear risk |
|  |  | Guo (2016)/China, Qingdao | Unclear risk | Unclear risk | Unclear risk | Unclear risk | Low risk | Unclear risk | Unclear risk |
|  |  | Liang (2016)/China, Dongguan | Unclear risk | Unclear risk | Unclear risk | Unclear risk | Low risk | Unclear risk | Unclear risk |
|  |  | Pan (2016)/ China, Dazhou | Unclear risk | Unclear risk | Unclear risk | Unclear risk | Low risk | Unclear risk | Unclear risk |
|  |  | Pan (2019)/China, Foshan | Low risk | Unclear risk | Unclear risk | Unclear risk | Low risk | Unclear risk | Unclear risk |
|  |  | Xue (2015)/China, Beijing | Unclear risk | Unclear risk | Unclear risk | Unclear risk | Low risk | Unclear risk | Unclear risk |
|  |  | Yu (2019)/China, Shiyan | Unclear risk | Unclear risk | Unclear risk | Unclear risk | Low risk | Unclear risk | Unclear risk |
|  |  | Wang (2018)/China, Dalian | Unclear risk | Unclear risk | Unclear risk | Unclear risk | Low risk | Unclear risk | Unclear risk |
|  |  | Wang (2022)/China, Hangzhou | Unclear risk | Unclear risk | Unclear risk | Unclear risk | Low risk | Unclear risk | Unclear risk |
|  |  | Yu (2019)/China, Wuxi | Unclear risk | Unclear risk | Unclear risk | Unclear risk | Low risk | Unclear risk | Unclear risk |
|  |  | Cao (2016)/China, Nanjing | Low risk | Unclear risk | Unclear risk | Unclear risk | Low risk | Unclear risk | Unclear risk |
| Moxibustion | Moxibustion-unspecified | Wang (2016)/China, Nanning | Unclear risk | Unclear risk | Unclear risk | Unclear risk | Low risk | Unclear risk | Unclear risk |
|  |  | Li (2011)/China, Guangzhou | Unclear risk | Unclear risk | Unclear risk | Unclear risk | Low risk | Unclear risk | Unclear risk |
|  | Ginger moxibustion (隔姜灸) | He (2013)/China, Shanghai ( | Unclear risk | Unclear risk | Unclear risk | Unclear risk | Low risk | Unclear risk | Unclear risk |
|  |  | Cui (2017)/China, Changzhi | Unclear risk | Unclear risk | Unclear risk | Unclear risk | Low risk | Unclear risk | Unclear risk |
|  | Heat-sensitive point’s moxibustion (热敏灸) | Liang (2018)/China, Foshan | Unclear risk | Unclear risk | Unclear risk | Unclear risk | Low risk | Unclear risk | Unclear risk |
|  |  | Chen (2017)/China, Nanchang | Unclear risk | Unclear risk | Unclear risk | Unclear risk | Low risk | Unclear risk | Unclear risk |
|  |  | Fan (2021)/China, Haikou | Low risk | Unclear risk | Unclear risk | Unclear risk | Low risk | Unclear risk | Unclear risk |
|  |  | Wang (2011)/China, Zhengzhou | Low risk | Low risk | Unclear risk | Unclear risk | Low risk | Unclear risk | Unclear risk |
|  |  | Zhe (2017)/China, Yan'an | Low risk | Unclear risk | Unclear risk | Unclear risk | Low risk | Unclear risk | Unclear risk |
|  |  | Cheng (2011)/China, Jiujiang | Unclear risk | Unclear risk | Unclear risk | Unclear risk | Low risk | Unclear risk | Unclear risk |
|  | Yi Fei moxibustion (益肺灸) | Zhao (2018)/China, Nanjing | Low risk | Unclear risk | Unclear risk | Unclear risk | Low risk | Unclear risk | Unclear risk |
|  |  | Li (2015)/China, Zhengzhou | Unclear risk | Unclear risk | Unclear risk | Unclear risk | Low risk | Unclear risk | Unclear risk |
|  |  | Huang (2021)/China, Zhengzhou | Unclear risk | Unclear risk | Unclear risk | Unclear risk | Low risk | Unclear risk | Unclear risk |
|  |  | Han (2017)/China, Yue Pu Hu Xian | Unclear risk | Unclear risk | Unclear risk | Unclear risk | Low risk | Unclear risk | Unclear risk |
|  |  | Qian (2014)/China, Zhengzhou | Low risk | Unclear risk | Unclear risk | Unclear risk | Low risk | Unclear risk | Unclear risk |
|  |  | Yang (2016)/China, Puyang | Unclear risk | Unclear risk | Unclear risk | Unclear risk | Low risk | Unclear risk | Unclear risk |
|  |  | Cui (2015)/China, Zhengzhou) | Low risk | Unclear risk | Unclear risk | Unclear risk | Low risk | Unclear risk | Unclear risk |
|  |  | Zhou (2011)/China, Zhengzhou | Low risk | Unclear risk | Unclear risk | Unclear risk | Low risk | Unclear risk | Unclear risk |
|  |  | Zhang (2012)/China, Zhengzhou | Unclear risk | Unclear risk | Unclear risk | Unclear risk | Low risk | Unclear risk | Unclear risk |
|  | Fire-dragon moxibustion (火龙灸) | Xu (2022)/China, Guangzhou | Low risk | Unclear risk | Unclear risk | Unclear risk | Low risk | Unclear risk | Unclear risk |
| Chuna |  | Rocha (2015)/Brazil | Low risk | Low risk | High risk | Low risk | Low risk | Unclear risk | Unclear risk |
|  |  | Noll (2008)/United States | Low risk | Unclear risk | Unclear risk | Low risk | Low risk | Unclear risk | Unclear risk |
|  |  | Maskey-Warzechowska (2019)/Poland | Low risk | Unclear risk | High risk | Unclear risk | Low risk | Unclear risk | Unclear risk |
|  |  | Kurzaj (2013)/Poland | Unclear risk | Unclear risk | Unclear risk | Unclear risk | Low risk | Unclear risk | Unclear risk |
|  |  | Buran Cirak (2022)/Turkey | Low risk | Low risk | Unclear risk | Unclear risk | Low risk | Unclear risk | Unclear risk |
|  |  | Chen (2006)/China, Shanghai | Unclear risk | Unclear risk | Unclear risk | Unclear risk | Low risk | Unclear risk | Unclear risk |
| Acupuncture | Acupuncture (针刺) | Suzuki (2008)/Japan | Unclear risk | Unclear risk | Unclear risk | Low risk | Low risk | Unclear risk | Unclear risk |
|  |  | Liu (2015)/China, Shanghai | Unclear risk | Unclear risk | Unclear risk | Unclear risk | Low risk | Unclear risk | Unclear risk |
|  |  | Deering (2011)/Ireland | Low risk | Unclear risk | Unclear risk | Low risk | Low risk | Unclear risk | Unclear risk |
|  |  | Suzuki (2012)/Japan | Low risk | Unclear risk | High risk | Low risk | Unclear risk | Unclear risk | Unclear risk |
|  |  | Li (2019)/China, Chengdu | Low risk | Unclear risk | High risk | Low risk | Low risk | Unclear risk | Unclear risk |
|  |  | Feng (2016)/China, Fuyang & Harbin | Low risk | Low risk | High risk | Low risk | Low risk | Unclear risk | Unclear risk |
|  |  | Zhong (2018)/China, Maoming | Unclear risk | Unclear risk | Unclear risk | Unclear risk | Low risk | Unclear risk | Unclear risk |
|  |  | Deng (2016)/China, Guangzhou | Unclear risk | Unclear risk | Unclear risk | Unclear risk | Low risk | Unclear risk | Unclear risk |
|  |  | Yang (2018)/China, Wuhan | Low risk | Unclear risk | Unclear risk | Unclear risk | Low risk | Unclear risk | Unclear risk |
|  |  | Jiao (2020)/China, Zhengzhou | Low risk | Unclear risk | Unclear risk | Unclear risk | Low risk | Unclear risk | Unclear risk |
|  |  | Tang (2017)/China, Fuzhou | Low risk | Unclear risk | Unclear risk | Unclear risk | Low risk | Unclear risk | Unclear risk |
|  |  | Li (2020)/China, Shenyang | Unclear risk | Unclear risk | Unclear risk | Unclear risk | Low risk | Unclear risk | Unclear risk |
|  |  | Wang (2021)/China, Chongqing | Low risk | Unclear risk | Unclear risk | Unclear risk | Low risk | Unclear risk | Unclear risk |
|  | Acu-TENS | Ngai (2010)/Hong Kong | Low risk | Low risk | High risk | Low risk | Low risk | Unclear risk | Unclear risk |
|  |  | Liu (2015)/China, Chengdu | Low risk | Low risk | High risk | Low risk | Low risk | Unclear risk | Unclear risk |
|  |  | Lau (2008)/Hong Kong | Low risk | Low risk | High risk | Low risk | Low risk | Unclear risk | Unclear risk |
|  |  | Jones (2011)/China, Sichuan province | Low risk | Low risk | High risk | Low risk | Low risk | Unclear risk | Unclear risk |
|  | Warm acupuncture (温针) | Yang (2016)/China, Xiamen | Unclear risk | Unclear risk | Unclear risk | Unclear risk | Unclear risk | Unclear risk | Unclear risk |
|  |  | Li (2015)/China, Xiamen | Low risk | Unclear risk | Unclear risk | Unclear risk | Unclear risk | Unclear risk | Unclear risk |
|  |  | Shi (2021)/China, Shanghai | Low risk | Unclear risk | Unclear risk | Unclear risk | Low risk | Unclear risk | Unclear risk |
|  | Auricular acupressure (耳穴贴压) | Pang (2014)/China, Weifang | Unclear risk | Unclear risk | Unclear risk | Unclear risk | High risk | Unclear risk | Unclear risk |
|  |  | Li (2017)/China, Fuzhou | Low risk | Unclear risk | Unclear risk | Unclear risk | Low risk | Unclear risk | Unclear risk |
|  |  | Jin (2009)/China, Wenzhou | Unclear risk | Unclear risk | Unclear risk | Unclear risk | Low risk | Unclear risk | Unclear risk |
|  | Electroacupuncture (电针) | He (2021)/China, Guangzhou | Low risk | Low risk | High risk | Low risk | High risk | Unclear risk | Unclear risk |
|  |  | Ge (2017)/China, Guangzhou | Low risk | Unclear risk | Unclear risk | Unclear risk | Low risk | Unclear risk | Unclear risk |
|  | Pressing needle (皮内针) | Chen (2018)/China, Deyang | Unclear risk | Unclear risk | Unclear risk | Unclear risk | Low risk | Unclear risk | Unclear risk |
|  |  | Peng (2015)/China, Chengdu | Low risk | Low risk | Unclear risk | Low risk | High risk | Unclear risk | Unclear risk |
|  |  | Wang (2019)/China, Shanghai | Low risk | Unclear risk | Unclear risk | Unclear risk | Low risk | Unclear risk | Unclear risk |
|  | Acupressure (穴位按揉) | Tuo (2018)/China, Wuzhou | Low risk | Unclear risk | Unclear risk | Unclear risk | Low risk | Unclear risk | Unclear risk |

Table S3. League table

The following league table contains the network estimates from network meta-analysis in the lower left triangle and the direct treatment estimates from pairwise comparisons in the upper right triangle.

1. FEV_1_ (L)

| **1** | 0.087 (-0.171 to 0.344) | . | 0.228 (0.118 to 0.338) | 0.248 (0.468 to 0.028) | 0.086 (-0.746 to 0.918) | 0.160 (-0.026 to 0.346) |
| --- | --- | --- | --- | --- | --- | --- |
| 0.086 (-0.063 to 0.234) | **2** | . | 0.140 (0.009 to 0.271) | 0.190 (-0.369 to 0.749) | 0.000 (-0.707 to 0.707) | . |
| 0.162 (-0.186 to 0.509) | 0.076 (-0.296 to 0.449) | **3** | . | . | -0.100 (-0.785 to 0.585) | 0.003 (-0.327 to 0.332) |
| 0.228 (0.122 to 0.334) | 0.142 (0.018 to 0.266) | 0.066 (-0.295 to 0.427) | **A** | . | . | . |
|  |  |  |  |  |  |  |
| 0.252 (0.046 to 0.458) | 0.166 (-0.076 to 0.408) | 0.090 (-0.314 to 0.493) | 0.024 (-0.203 to 0.250) | **B** | . | . |
| 0.077 (-0.371 to 0.524) | -0.009 (-0.462 to 0.444) | -0.085 (-0.562 to 0.392) | -0.151 (-0.603 to 0.301) | -0.175 (-0.665 to 0.315) | **C** | . |
| 0.161 (-0.021 to 0.344) | 0.076 (-0.157 to 0.309) | -0.001 (-0.310 to 0.309) | -0.067 (-0.277 to 0.143) | -0.090 (-0.365 to 0.185) | 0.085 (-0.375 to 0.544) | **D** |

1. FEV_1_ (%)

| **1** | 2.107 (-3.604 to 7.818) | . | 4.646 (1.839 to 7.453) | 6.064 (1.844 to 10.285) | 3.970 (-15.316 to 23.256) | 2.563 (-2.294 to 7.419) |
| --- | --- | --- | --- | --- | --- | --- |
| 0.378 (-3.627 to 4.383) | **2** | . | 3.173 (-1.124 to 7.470) | . | 8.616 (-4.928 to 22.160) | 8.545 (-1.710 to 18.799) |
| -1.119 (-14.101 to 11.862) | -1.497 (-14.721 to 11.727) | **3** | . | . | -2.000 (-24.989 to 20.989) | 8.334 (-5.980 to 22.647) |
| 4.441 (1.724 to 7.158) | 4.064 (0.244 to 7.883) | 5.561 (-7.601 to 18.722) | **A** | . | . | . |
| 6.064 (1.844 to 10.285) | 5.687 (-0.132 to 11.505) | 7.184 (-6.467 to 20.834) | 1.623 (-3.396 to 6.642) | **B** | . | . |
| 5.363 (-5.192 to 15.917) | 4.985 (-5.416 to 15.386) | 6.482 (-8.047 to 21.011) | 0.921 (-9.731 to 11.574) | -0.702 (-12.069 to 10.666) | **C** | . |
| 3.926 (-0.536 to 8.389) | 3.549 (-1.927 to 9.024) | 5.046 (-7.491 to 17.583) | -0.515 (-5.579 to 4.550) | -2.138 (-8.280 to 4.004) | -1.436 (-12.490 to 9.617) | **D** |

1. 6MWD (m)

| **1** | 19.497 (-2.419 to 41.413) | . | 45.372 (34.459 to 56.285) | 34.741 (-0.479 to 69.961) | 43.080 (-35.927 to 122.087) | 40.096 (18.662 to 61.529) |
| --- | --- | --- | --- | --- | --- | --- |
| 17.430 (3.182 to 31.679) | **2** | . | 23.625 (10.303 to 36.946) | 45.300 (-16.195 to 106.795) | 46.050 (-14.687 to 106.787) | 65.678 (1.085 to 130.272) |
| 38.856 (-8.149 to 85.862) | 21.426 (-26.804 to 69.656) | **3** | . | . | 46.620 (-42.381 to 135.621) | -2.589 (-51.463 to 46.286) |
| 44.941 (34.448 to 55.434) | 27.511 (15.083 to 39.939) | 6.085 (-41.726 to 53.895) | **A** | . | . | . |
| 41.654 (10.890 to 72.419) | 24.224 (-8.167 to 56.615) | 2.798 (-53.190 to 58.786) | -3.286 (-35.183 to 28.610) | **B** | . | . |
| 62.599 (18.277 to 106.921) | 45.169 (1.004 to 89.333) | 23.743 (-32.216 to 79.701) | 17.658 (-26.949 to 62.265) | 20.944 (-32.509 to 74.398) | **C** | . |
| 43.167 (23.081 to 63.252) | 25.736 (2.086 to 49.387) | 4.310 (-9.884 to 48.505) | -1.774 (-24.026 to 20.477) | 1.512 (-35.070 to 38.094) | -19.432 (-66.289 to 27.425) | **D** |

**Abbreviations:** FEV_1_, forced expiratory volume in a 1 second; 6MWD, 6-minute walking distance

**Supplement 4. PMA results**

1. FEV_1_ (L) in PMA

Abbreviations: FEV1, forced expiratory volume in a 1 second; PMA, pairwise meta-analysis; MD, mean difference; CI, confidence interval

A1. Forest plot of qigong in the experimental group compared with the controls


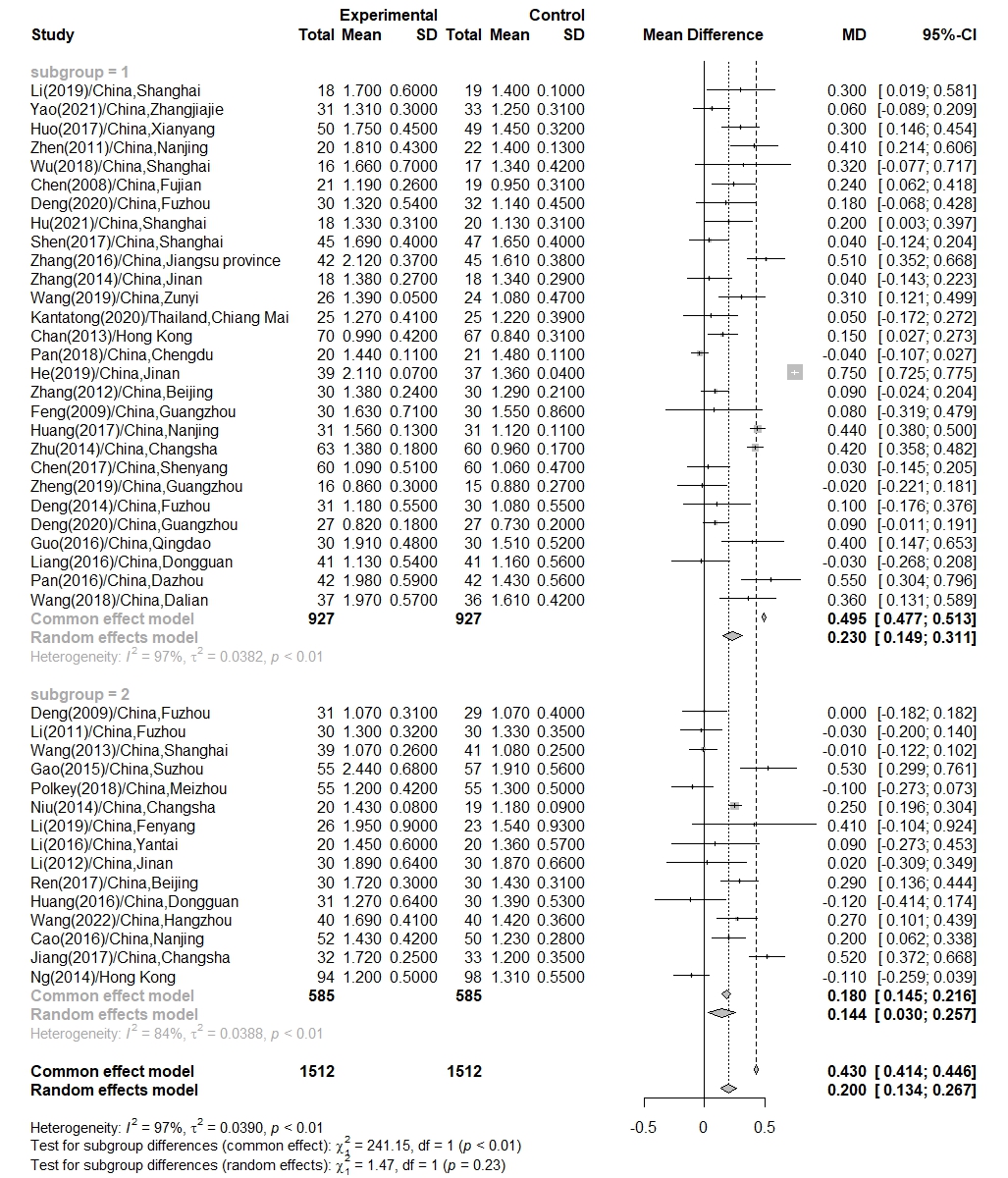


A2. Funnel plot of qigong


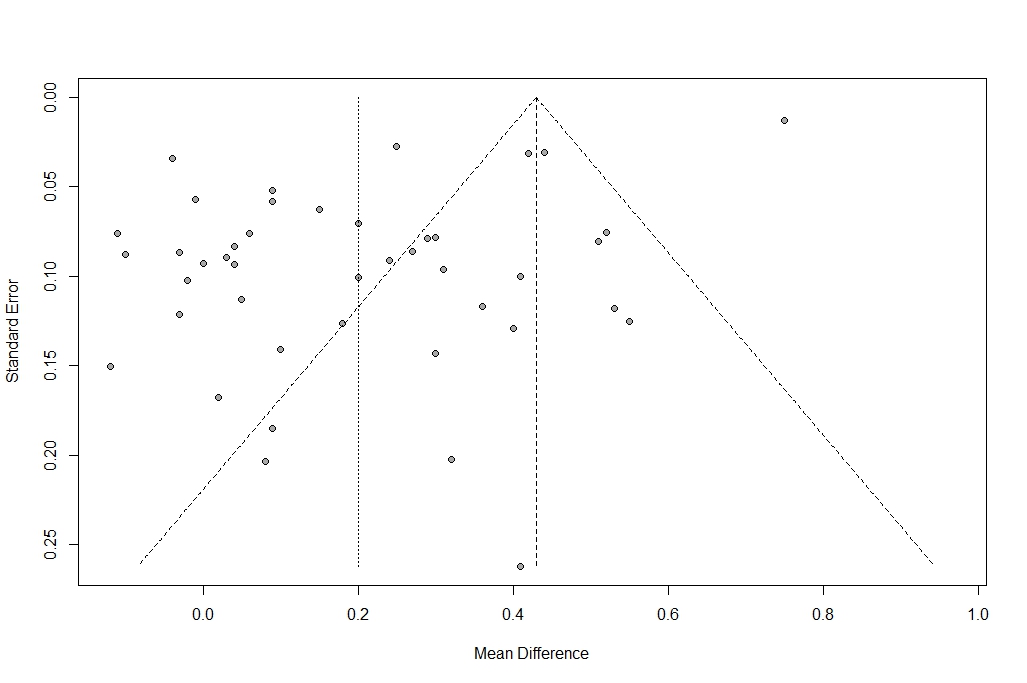


B1. Forest plot of moxibustion in the experimental group compared with the controls


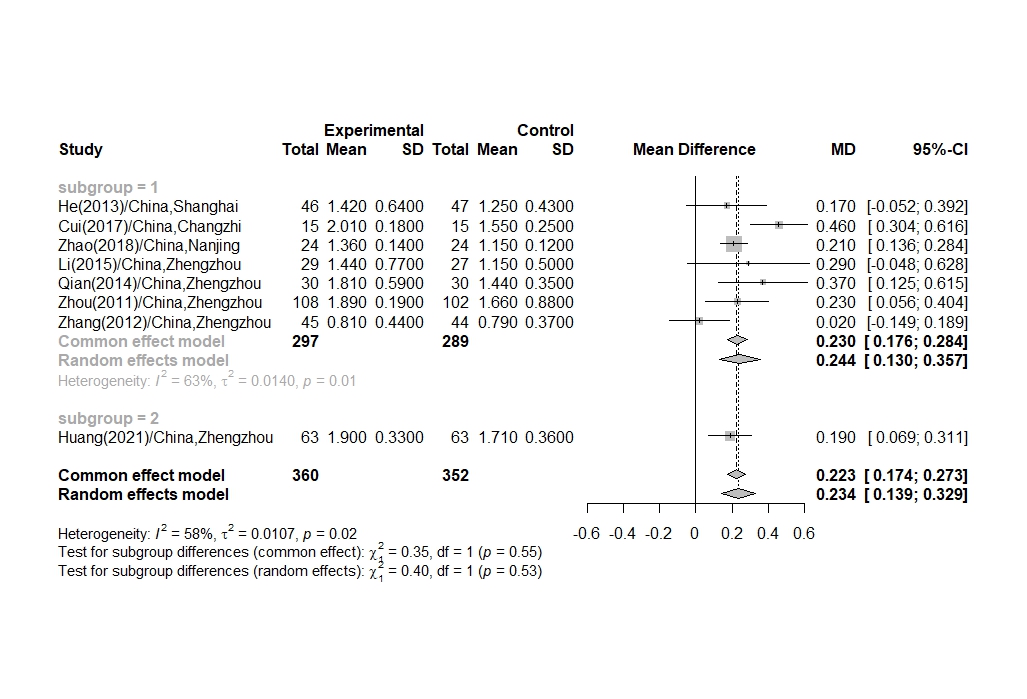


C1. Forest plot of chuna in the experimental group compared with the controls


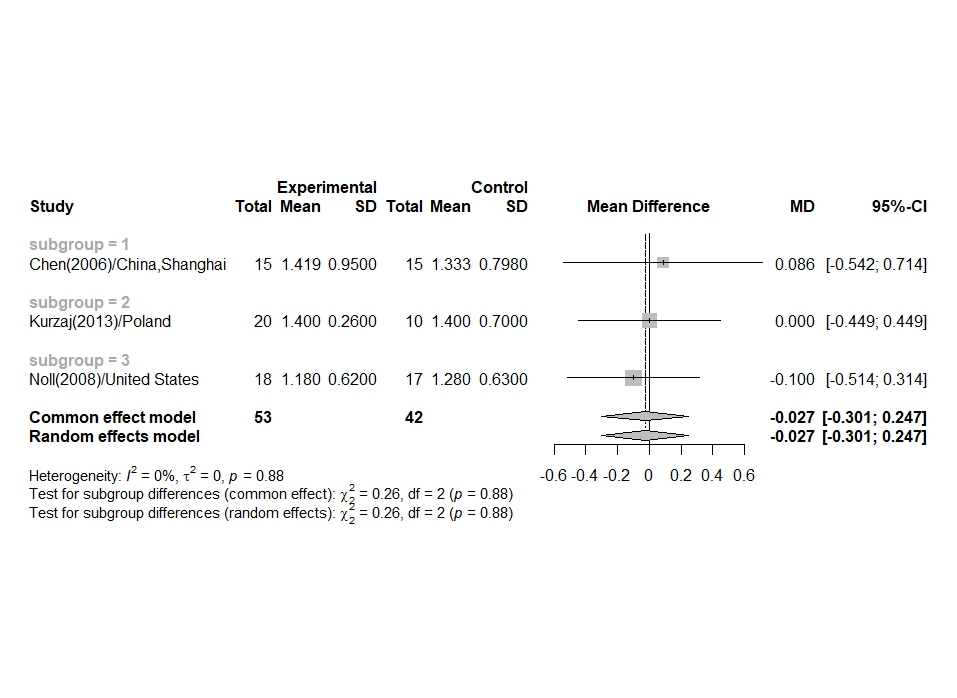


D1. Forest plot of acupuncture in the experimental group compared with the controls


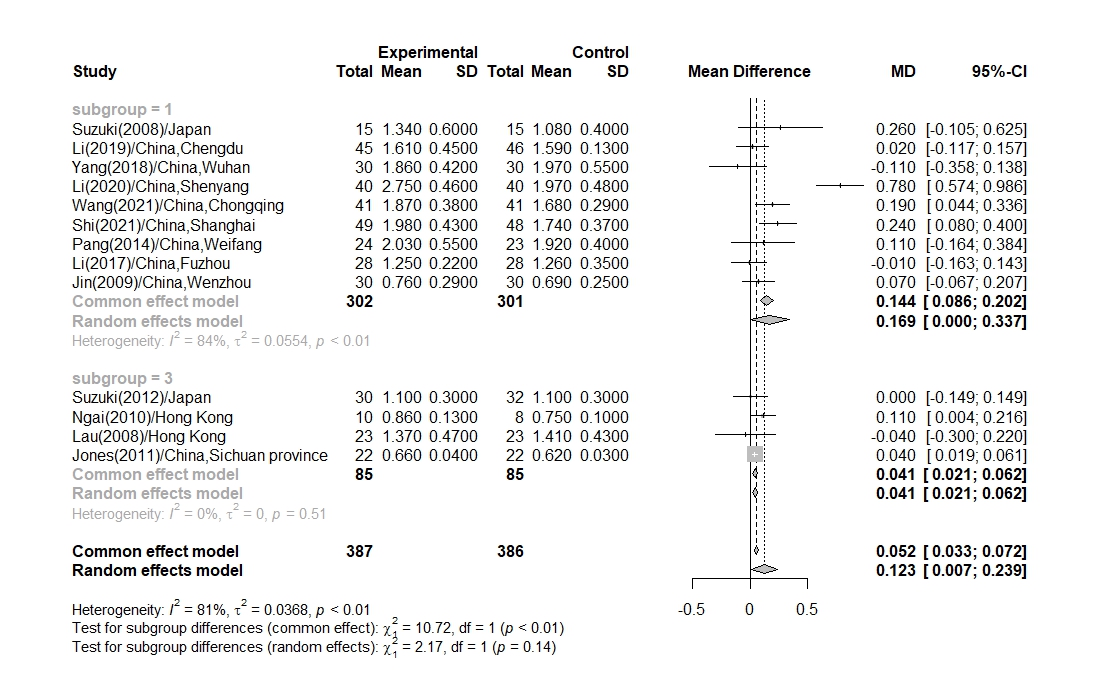


D2. Funnel plot of acupuncture


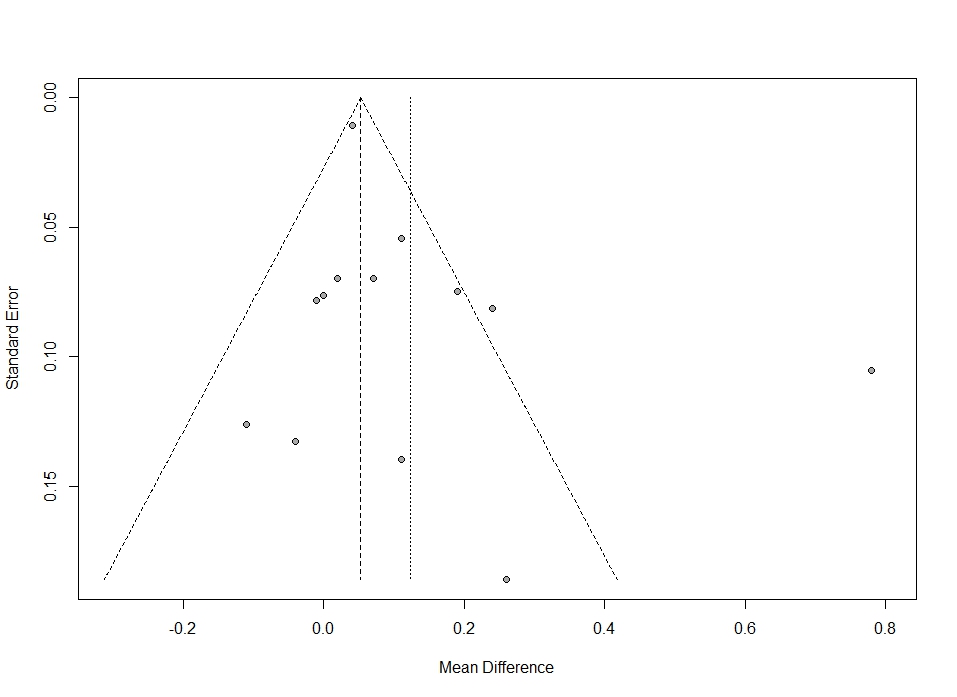


2. FEV_1_ (%) in PMA

Abbreviations: FEV1, forced expiratory volume in a 1 second; PMA, pairwise meta-analysis; MD, mean difference; CI, confidence interval

A1. Forest plot of qigong in the experimental group compared with the controls


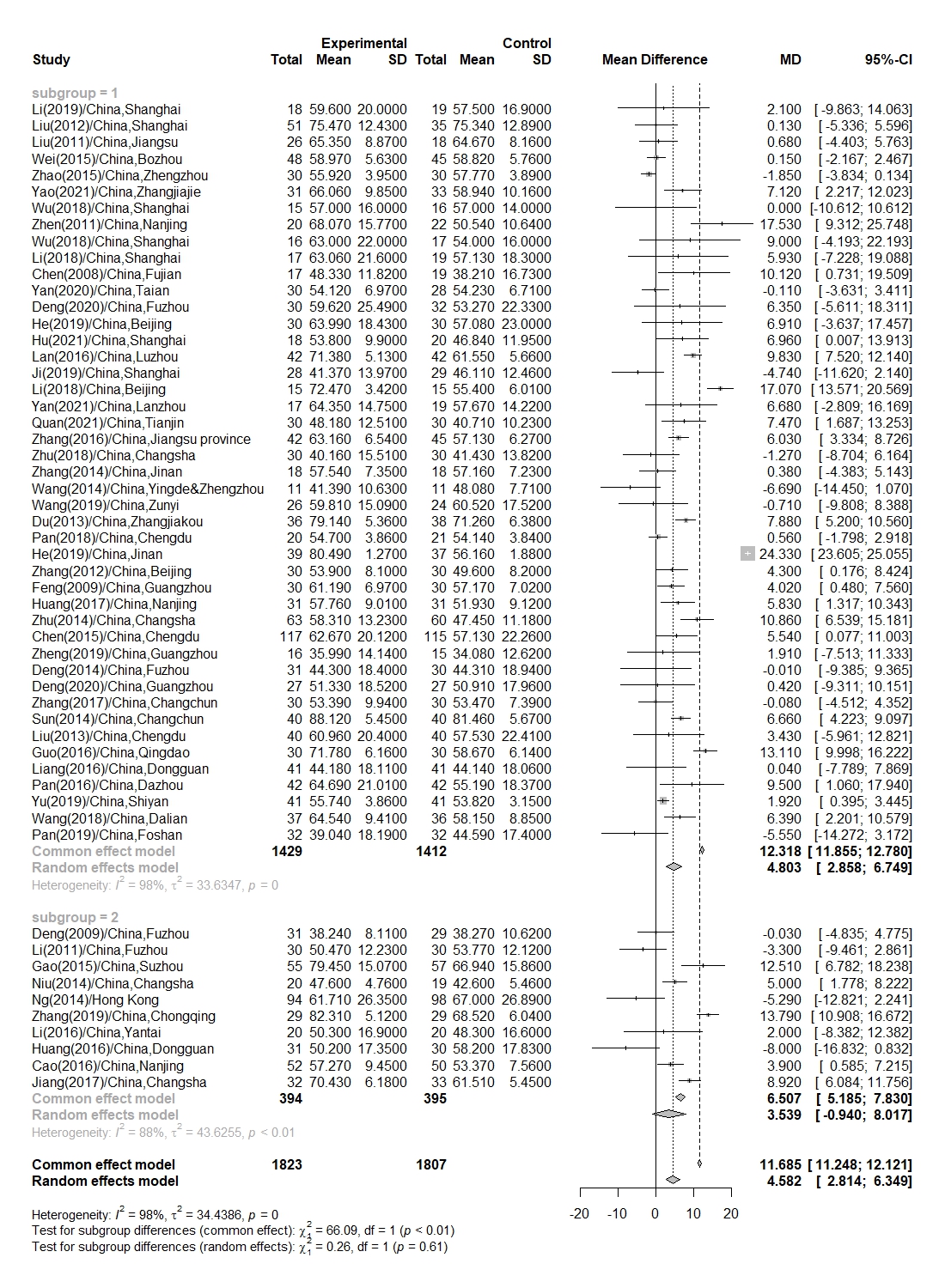


A2. Funnel plot of qigong


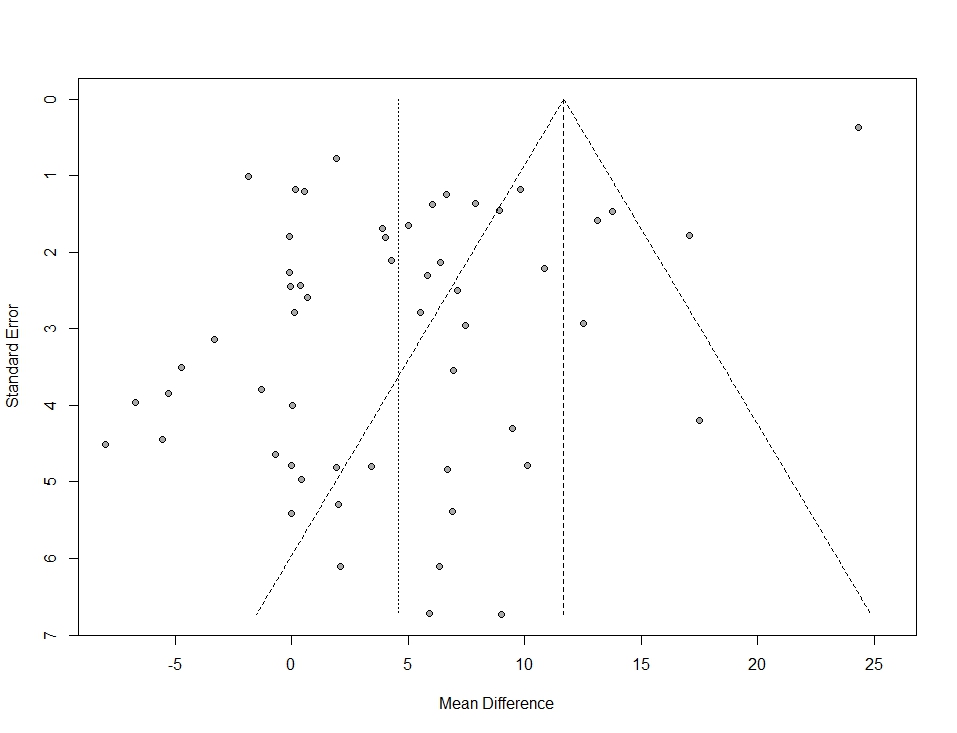


B1. Forest plot of moxibustion in the experimental group compared with the controls


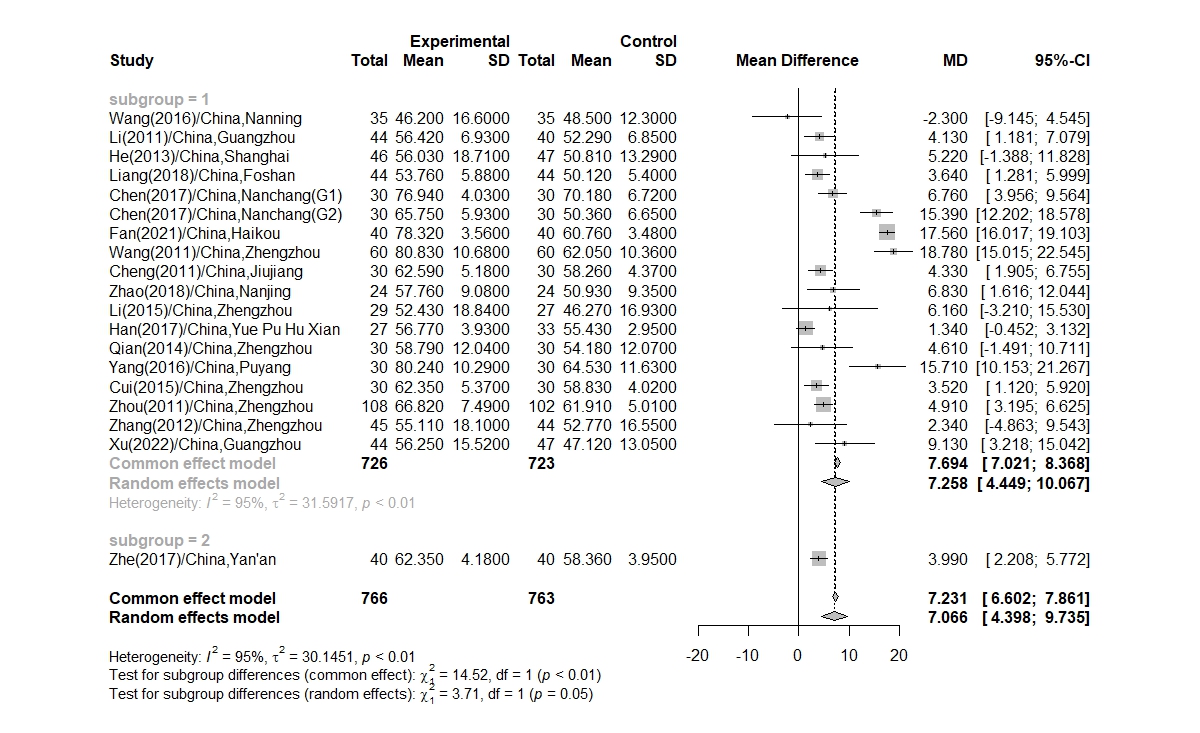


B2. Funnel plot of moxibustion


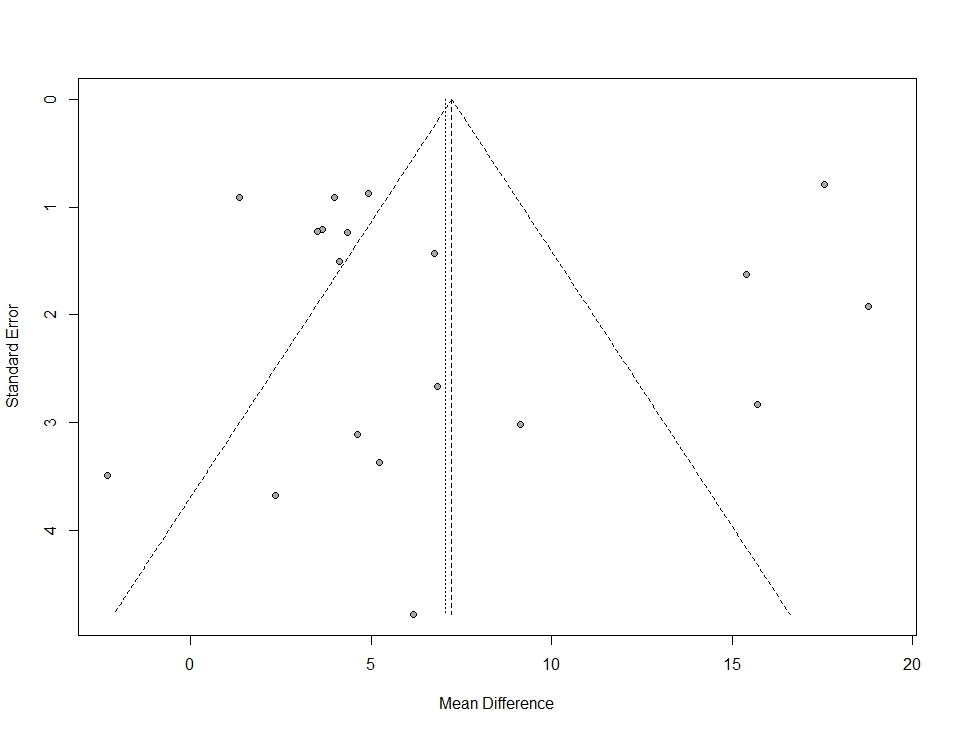


C1. Forest plot of chuna in the experimental group compared with the controls


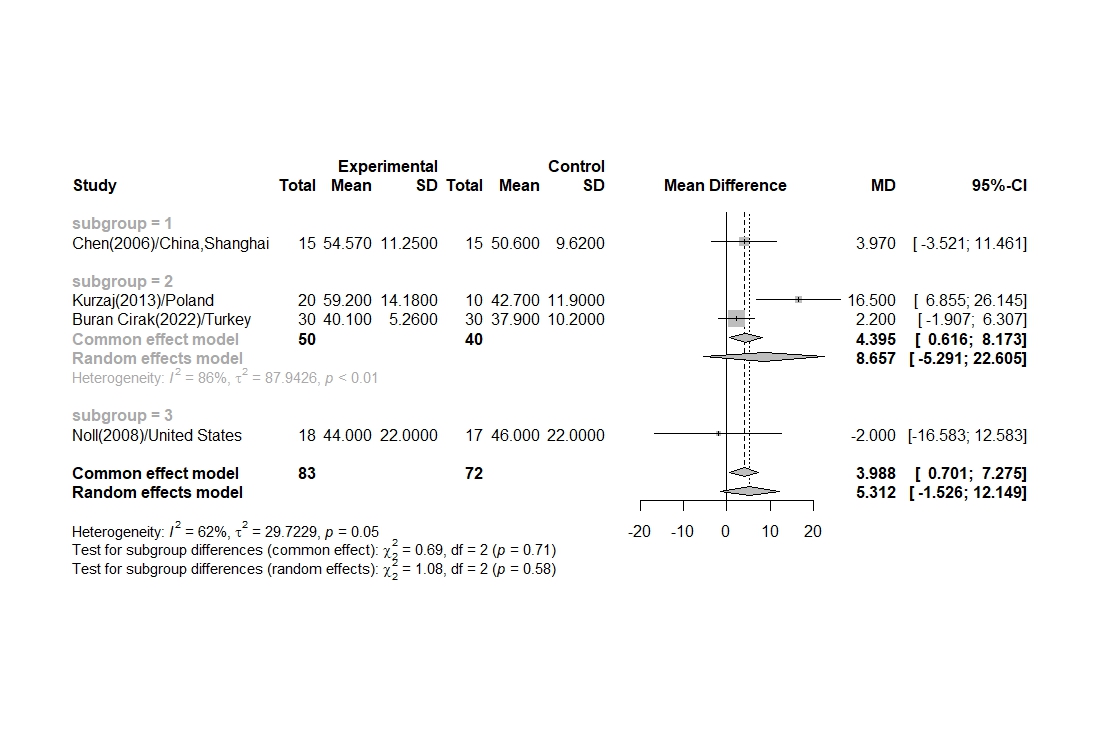


D1. Forest plot of acupuncture in the experimental group compared with the controls


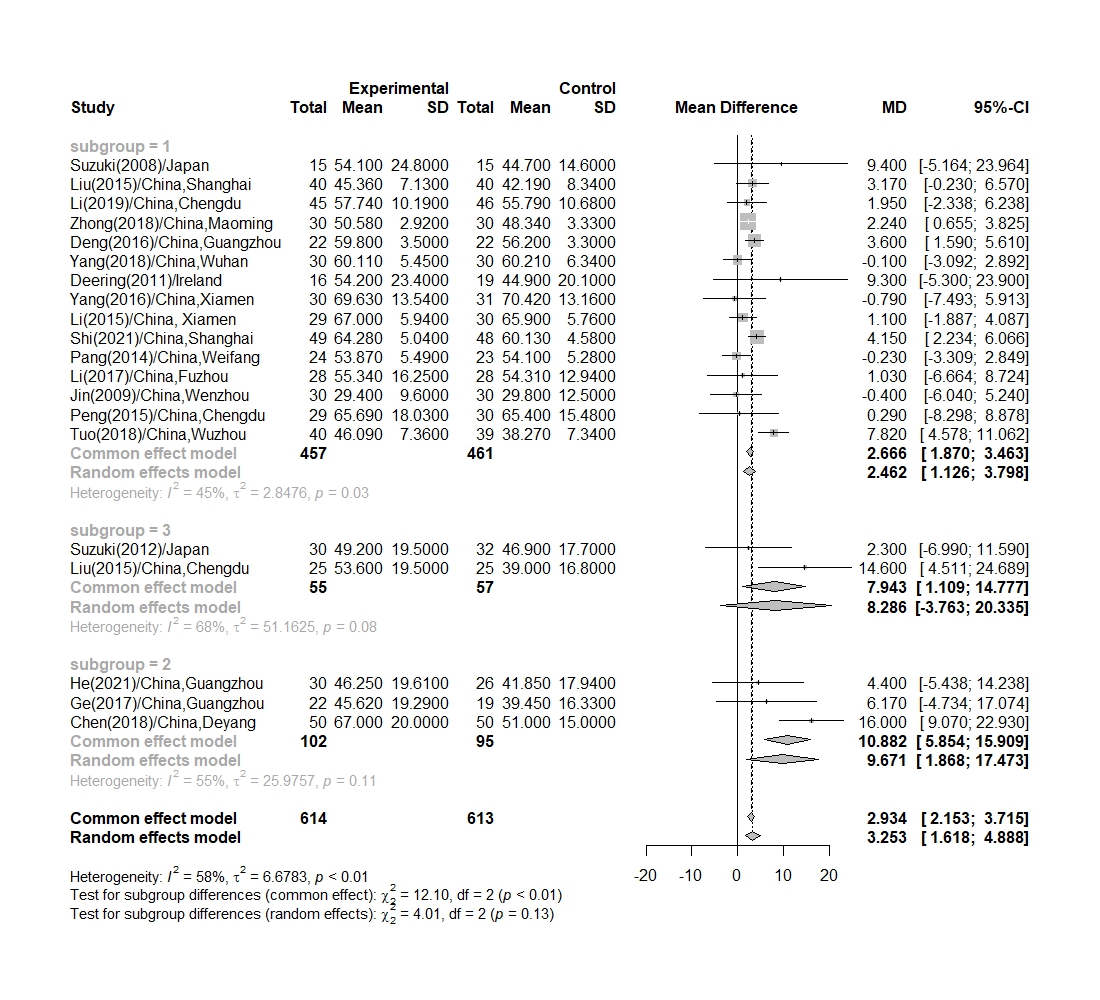


D2. Funnel plot of acupuncture


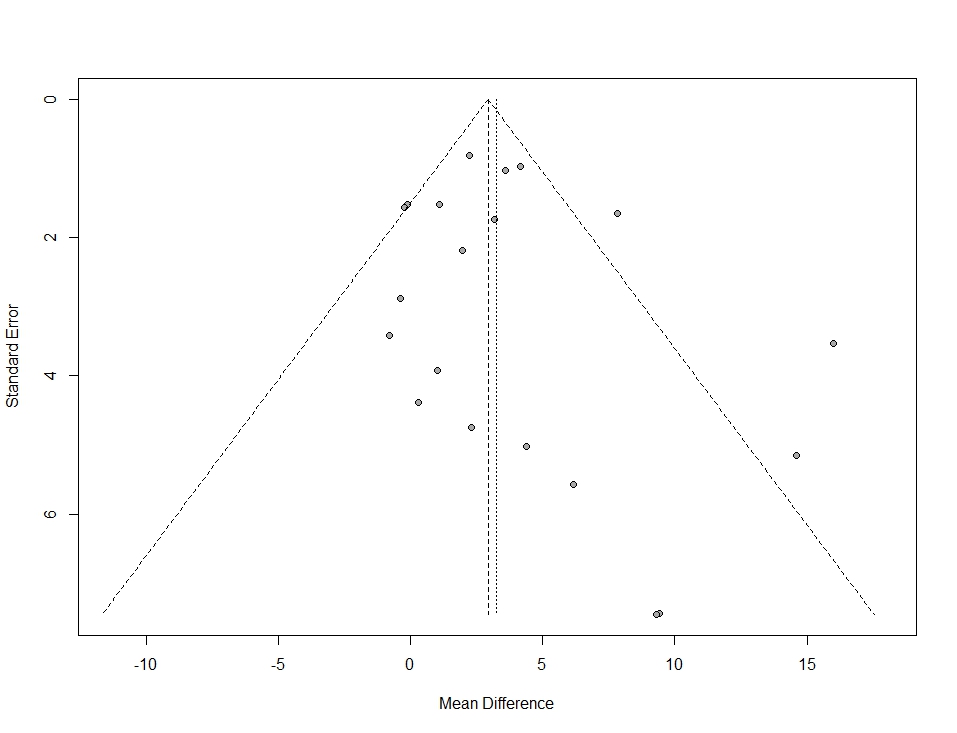


3. 6MWD (m) in PMA

Abbreviations: 6MWD, 6-minute walking distance, pairwise meta-analysis; MD, mean difference; CI, confidence interval

A1. Forest plot of qigong in the experimental group compared with the controls


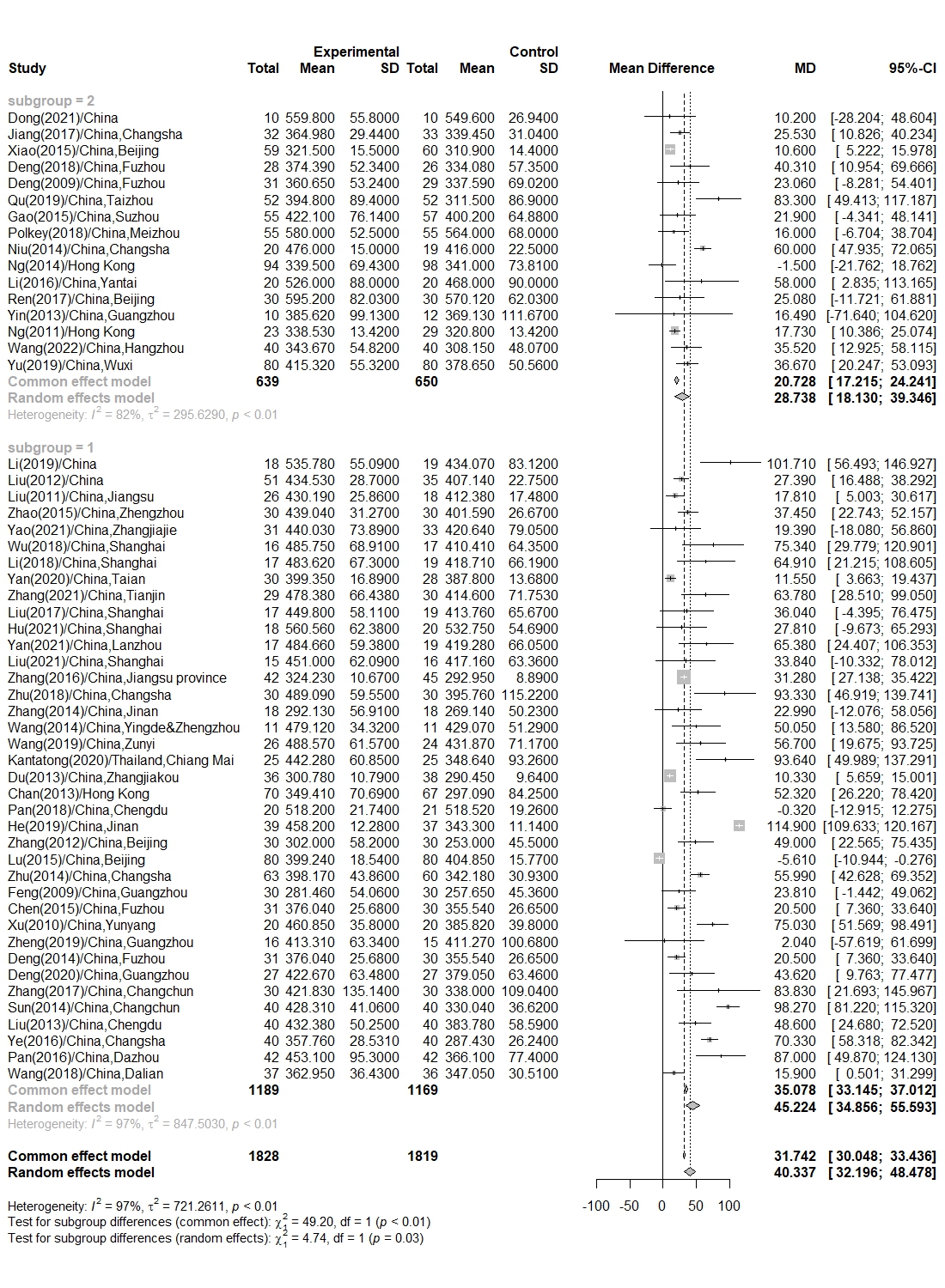


A2. Funnel plot of qigong


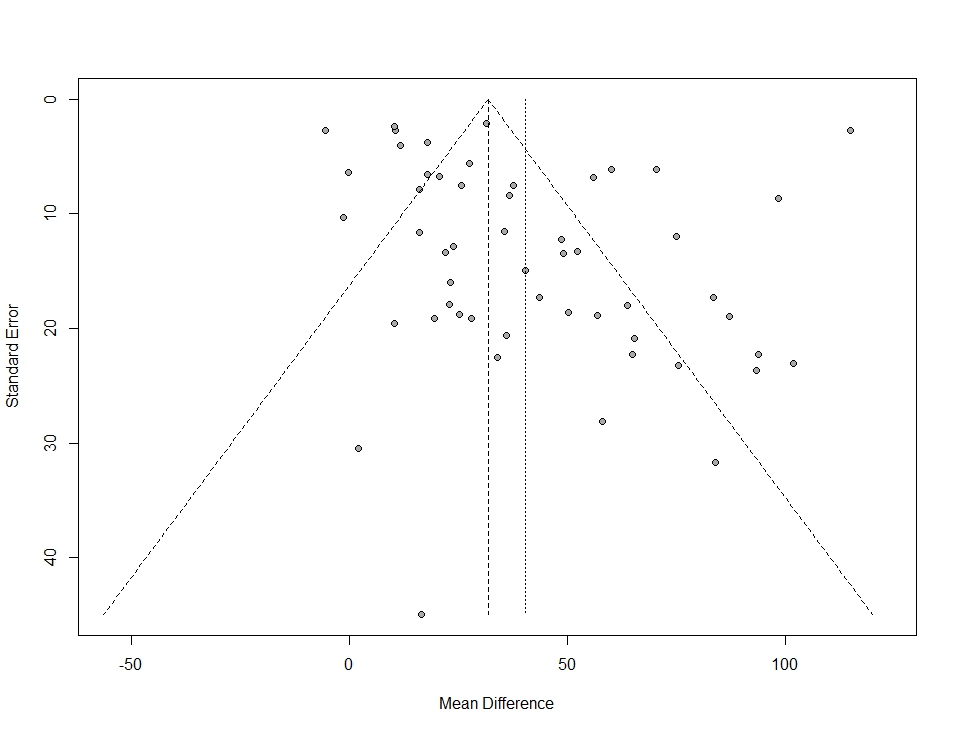


B1. Forest plot of moxibustion in the experimental group compared with the controls


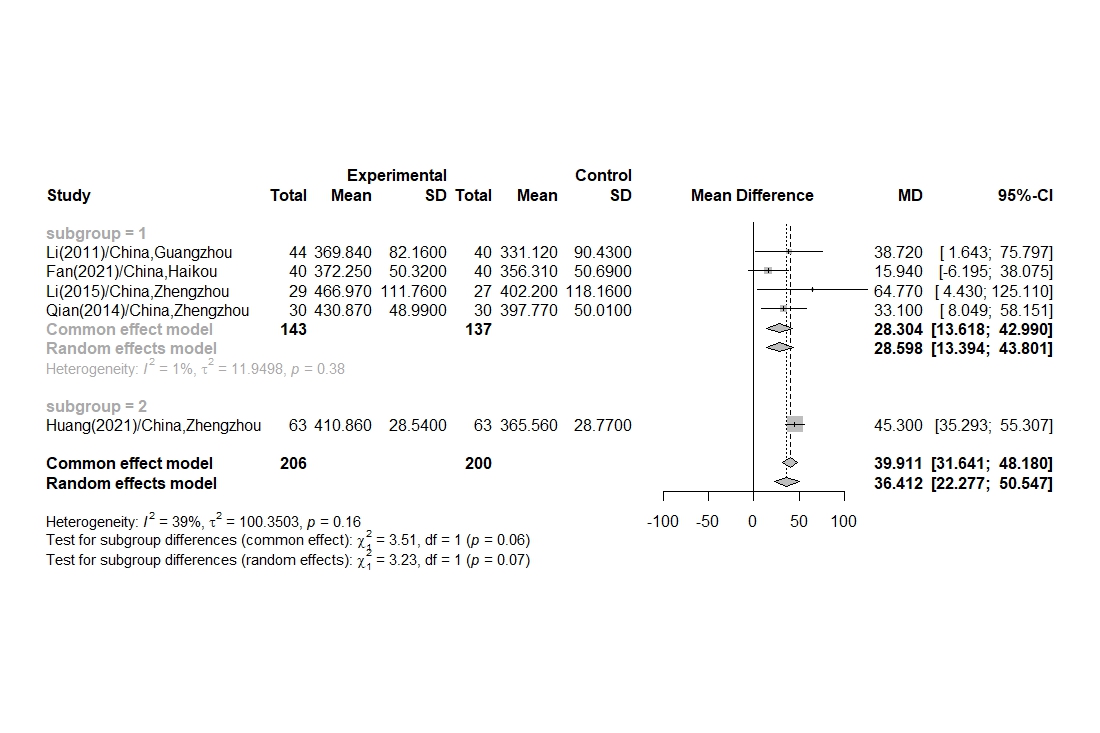


C1. Forest plot of chuna in the experimental group compared with the controls


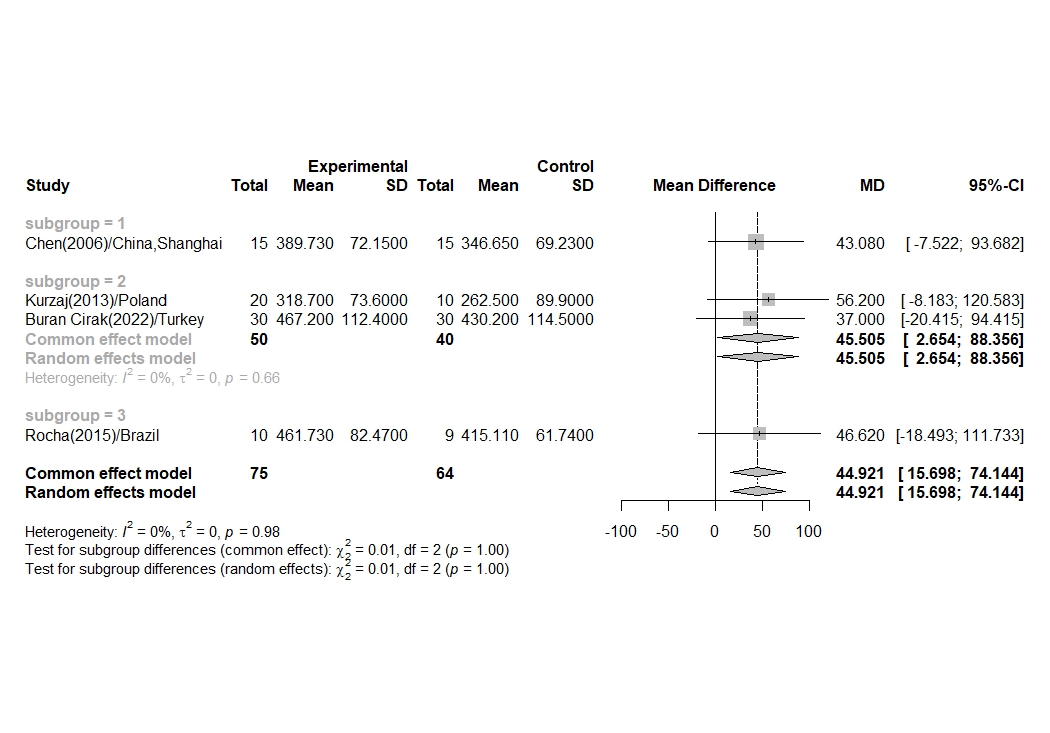


D1. Forest plot of acupuncture in the experimental group compared with the controls


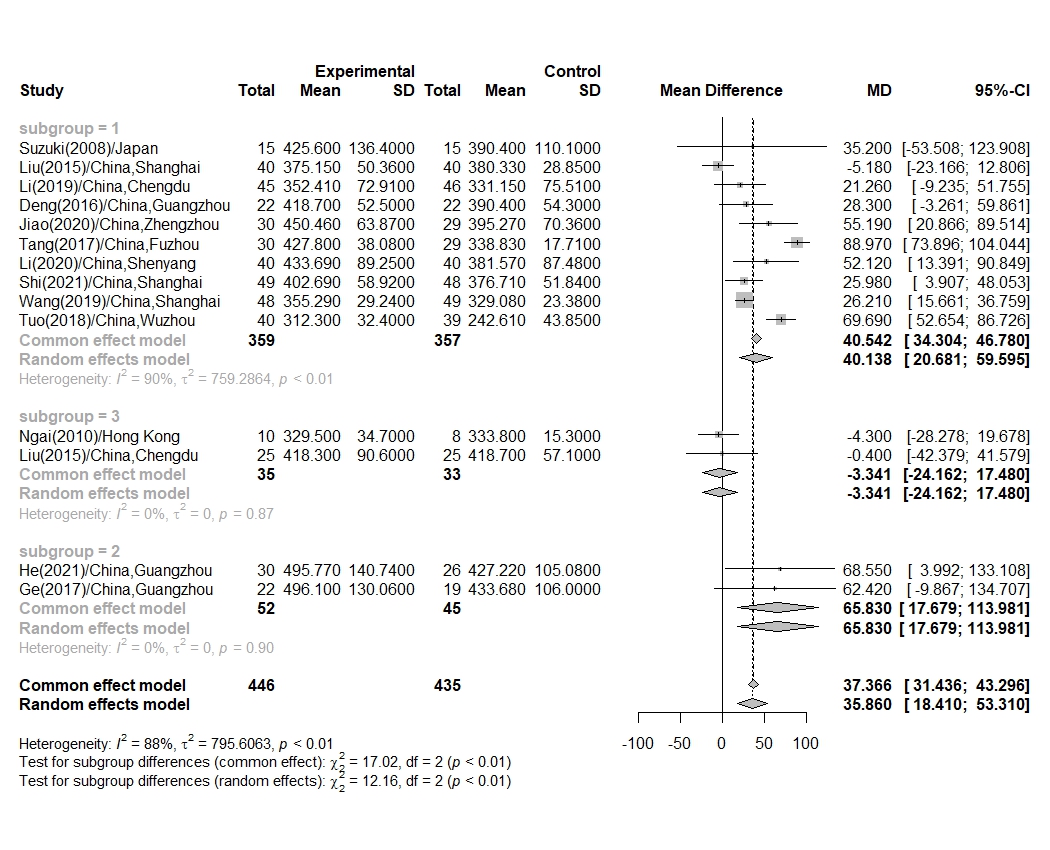


D2. Funnel plot of acupuncture


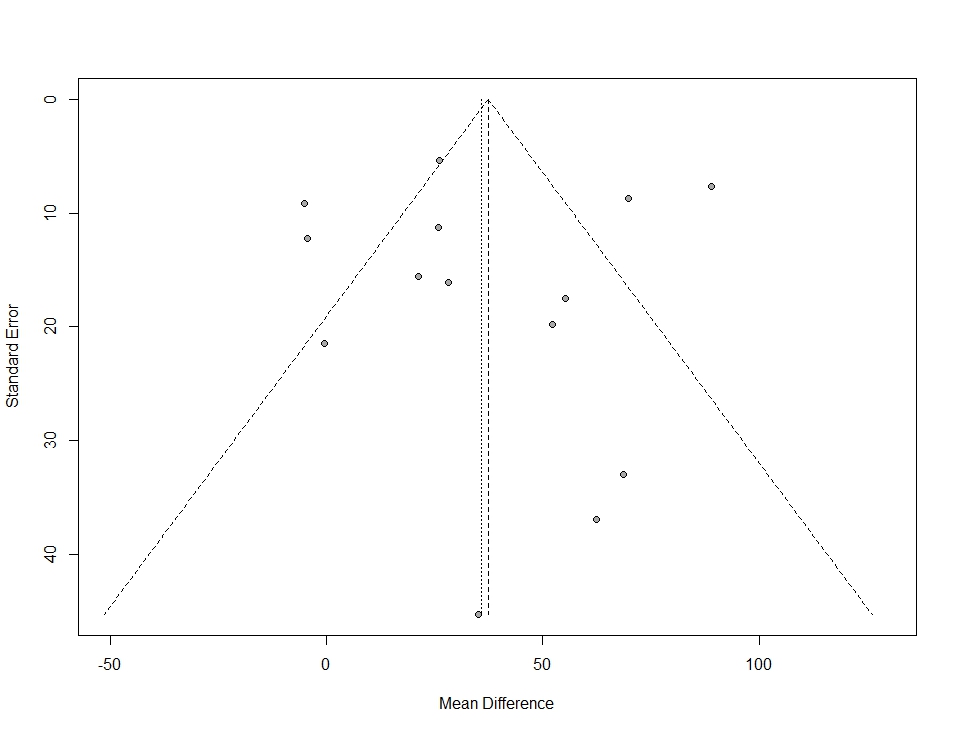


**Supplement 5. Figures**

Figure S1. Network summary and summary of inconsistency test results (global approach)

**Abbreviations:** FEV_1_, forced expiratory volume in a 1 second; 6MWD, 6-minute walking distance

1. Network Summary of FEV_1_ (L)


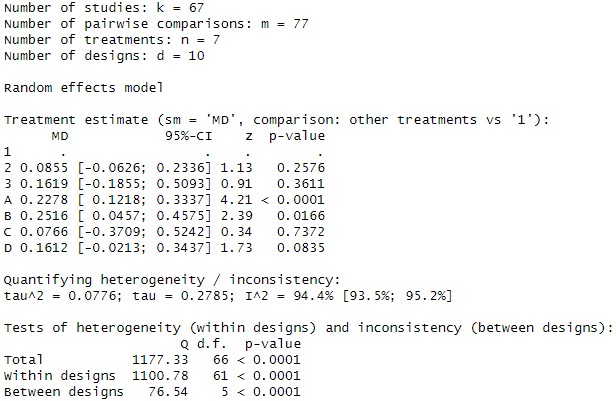


1. Global approach of FEV_1_ (L)


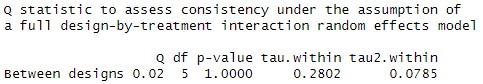


1. Network summary of FEV_1_ (%)


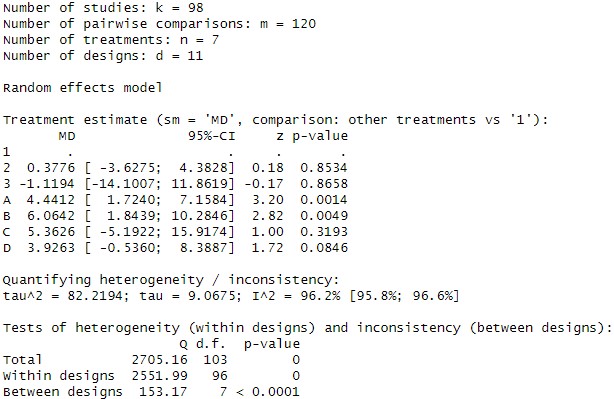


1. Global approach of FEV_1_ (%)


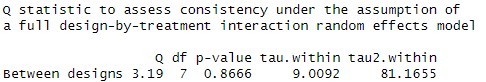


1. Network summary of 6MWD (m)


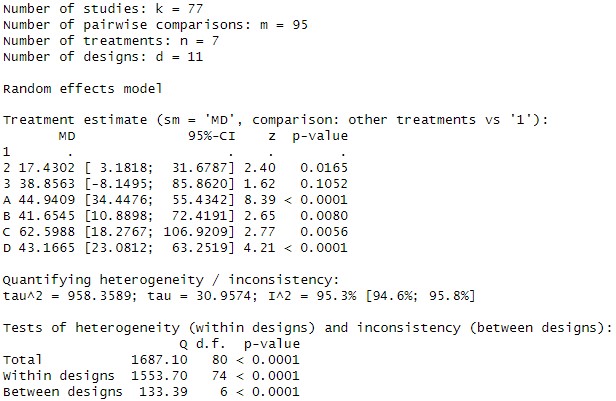


1. Global approach of 6MWD (m)


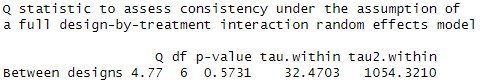


Figure S2. Summary of inconsistency test results (local approach)

**Abbreviations:** FEV_1_, forced expiratory volume in a 1 second; 6MWD, 6-minute walking distance

1. FEV_1_ (L)


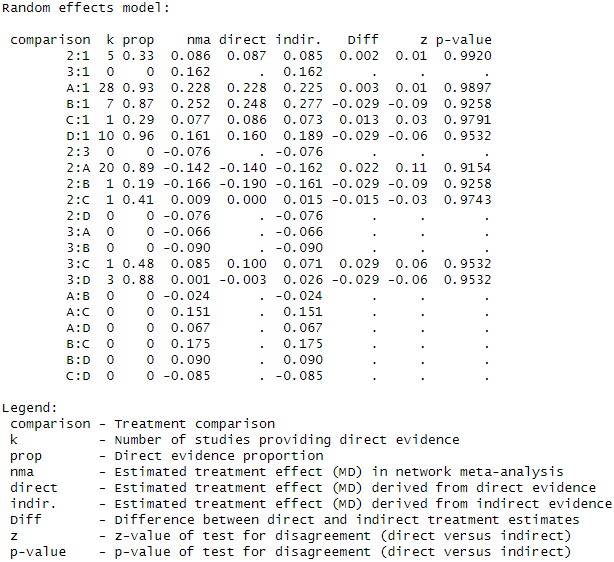


1. FEV_1_ (%)


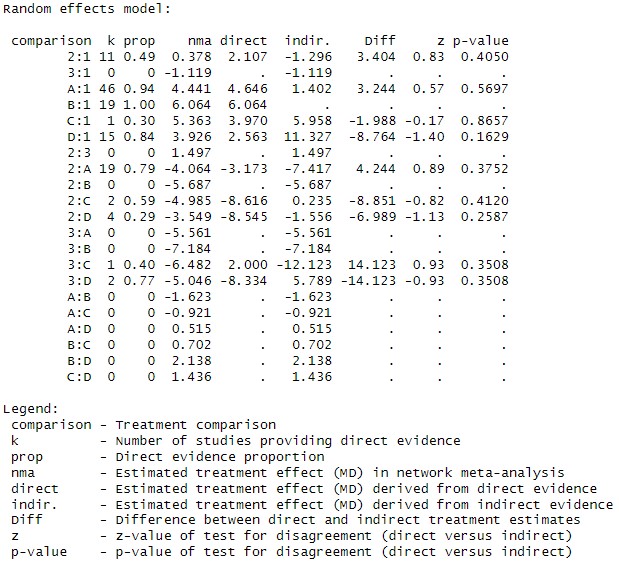


1. 6MWD (m)


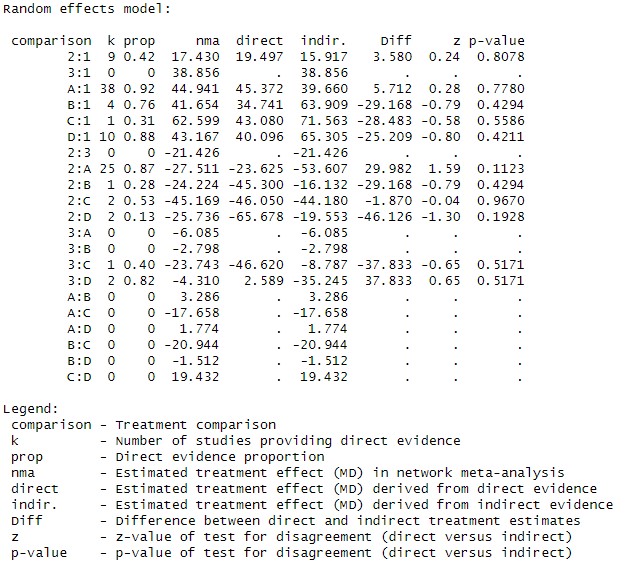


Figure S3. Plot of inconsistency test results (local approach)

**Abbreviations:** FEV_1_, forced expiratory volume in a 1 second; 6MWD, 6-minute walking distance

1. FEV_1_ (L)


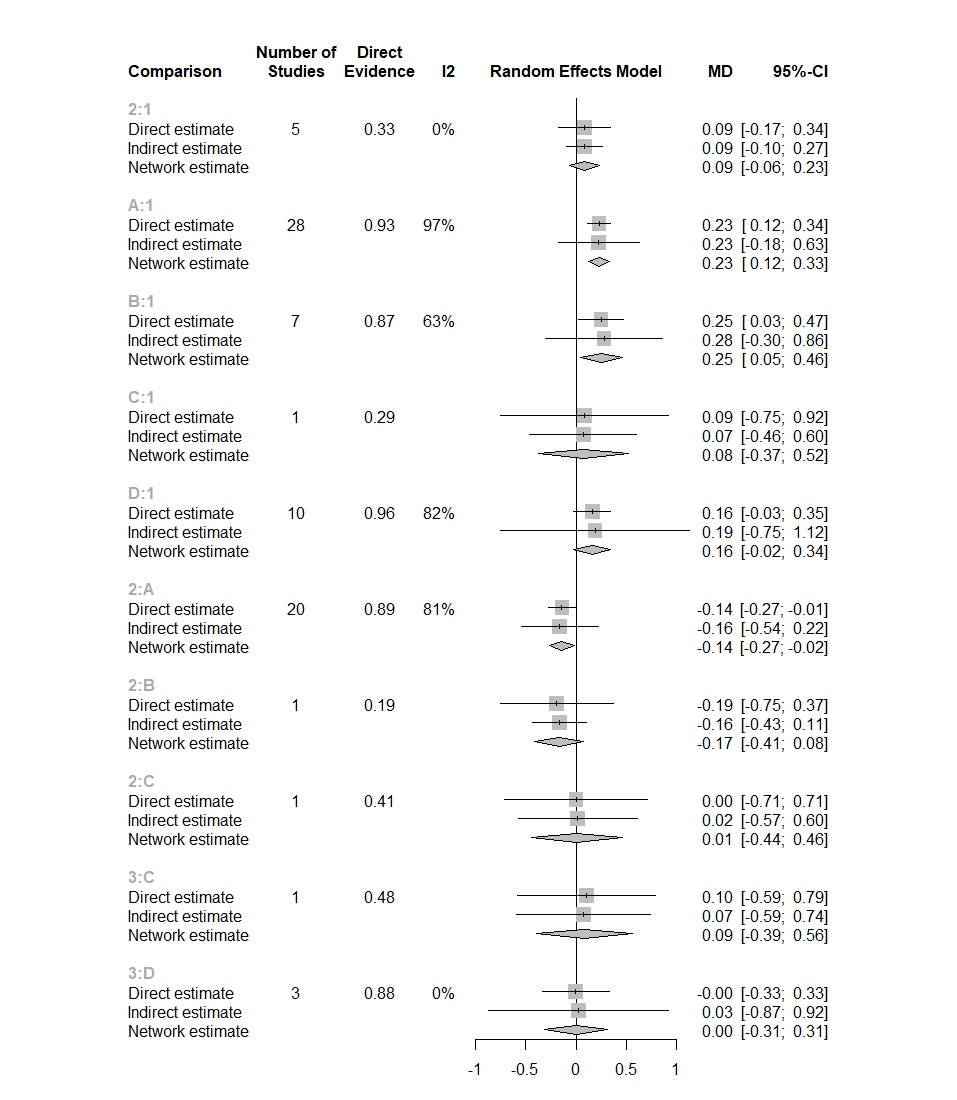


1. FEV_1_ (%)


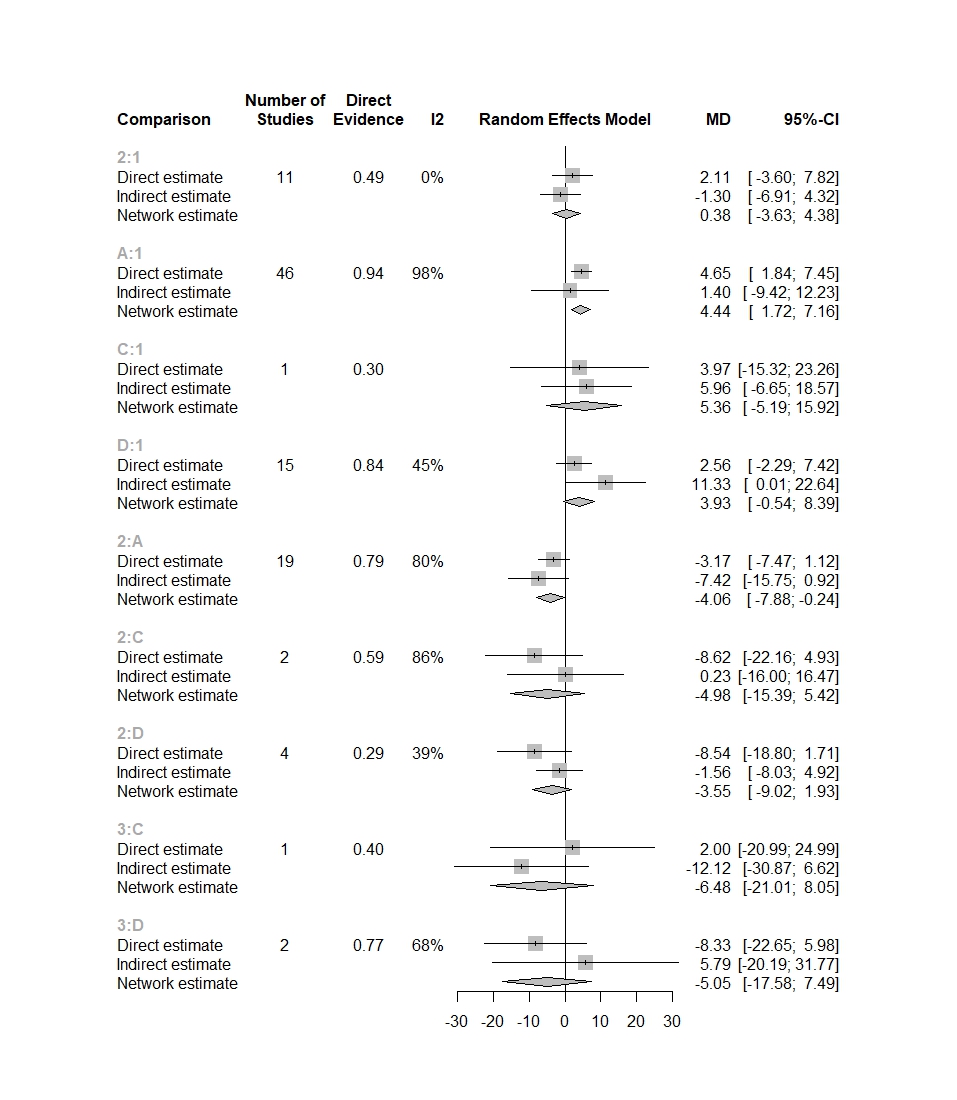


1. 6MWD (m)


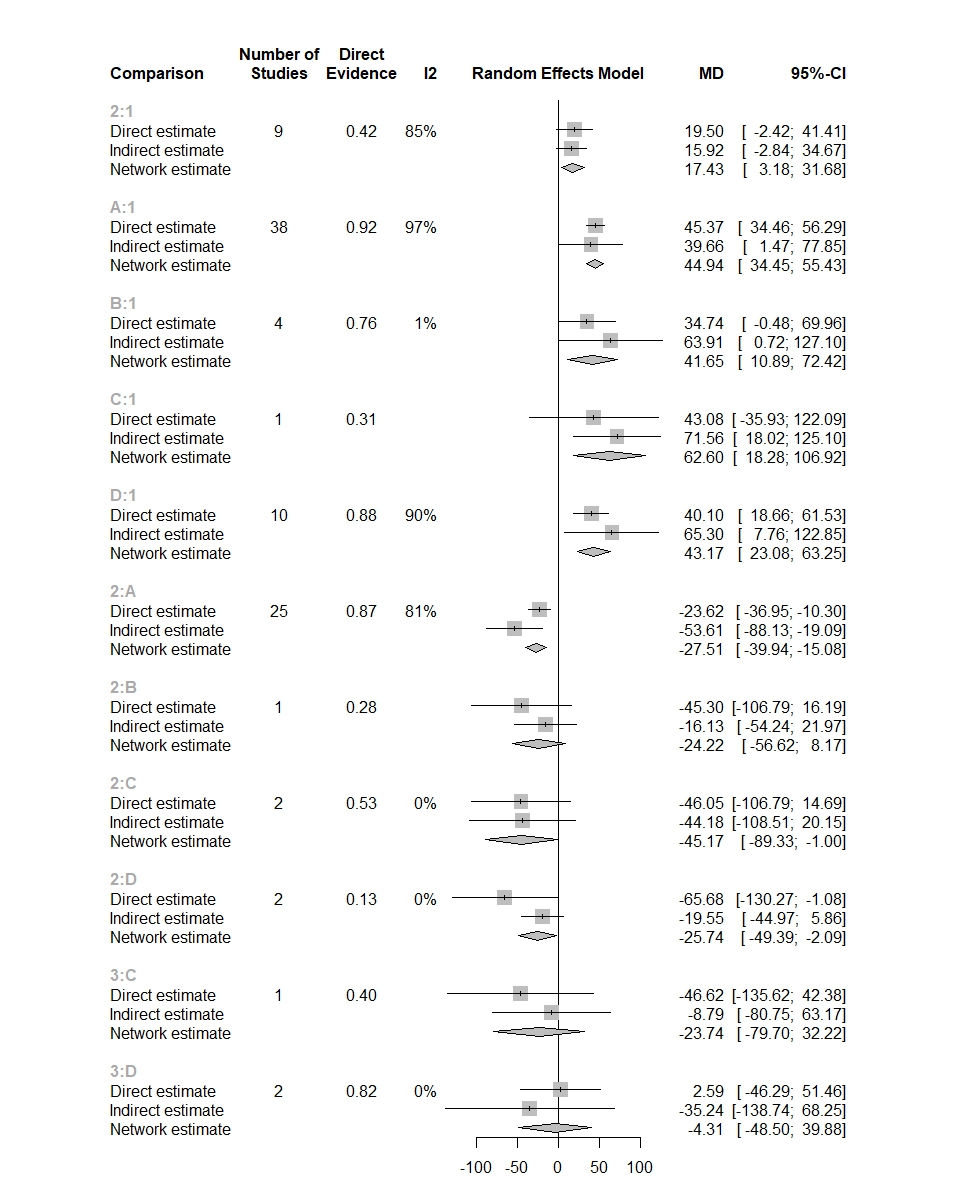


Figure S4. Funnel plot with Egger test values

**Abbreviations:** FEV_1_, forced expiratory volume in a 1 second; 6MWD, 6-minute walking distance

1. FEV_1_ (L)


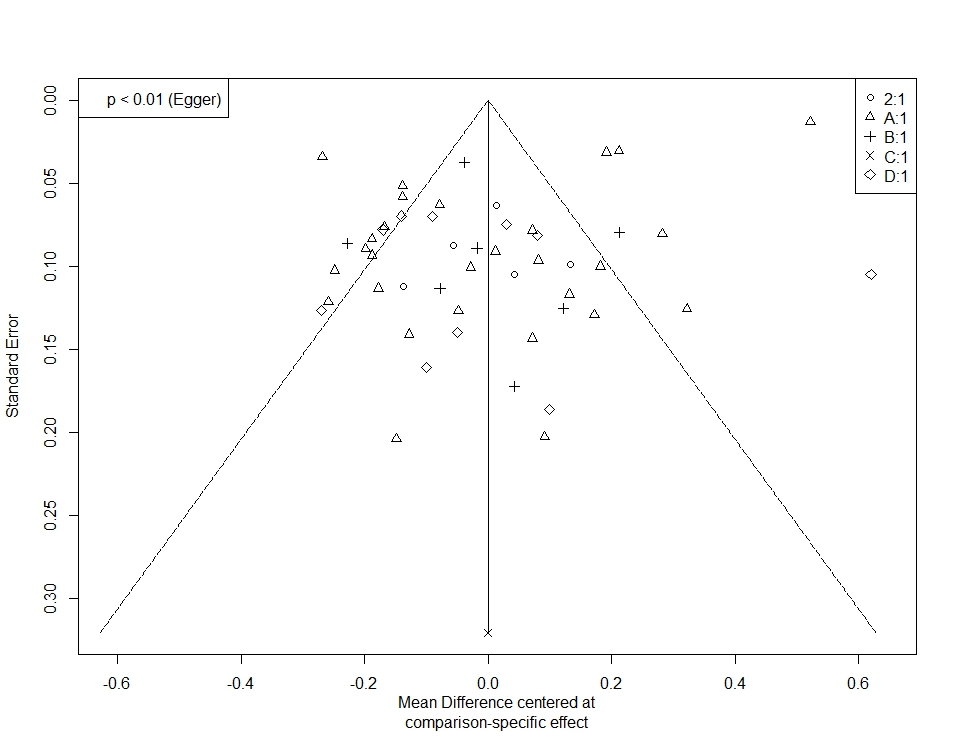


1. FEV_1_ (%)


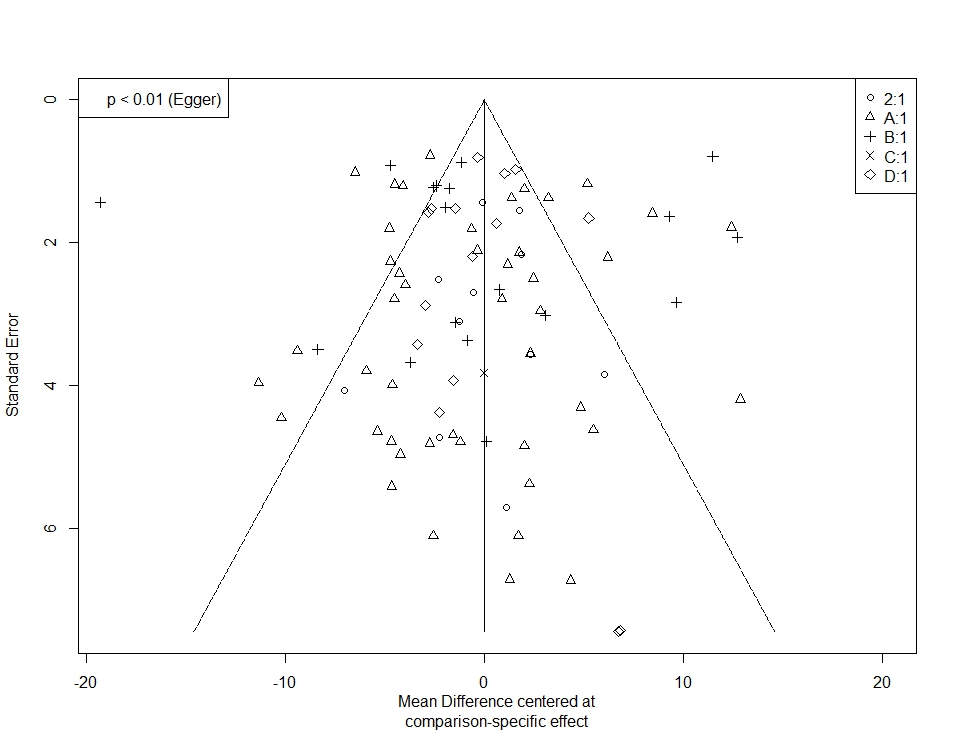


1. 6MWD (m)


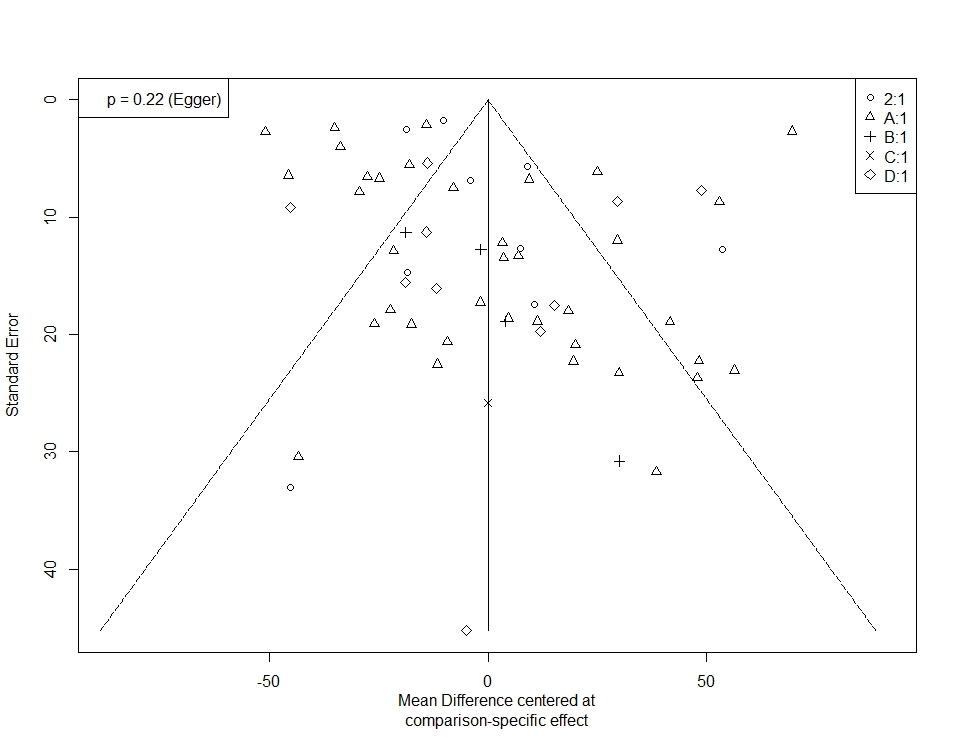

Supplement: Supplementary file 1 [file Supplementary_file_1.docx]
